# Scaffolding protein CcmM directs multiprotein phase separation in β-carboxysome biogenesis

**DOI:** 10.1038/s41594-021-00676-5

**Published:** 2021-11-10

**Authors:** Kun Zang, Huping Wang, F. Ulrich Hartl, Manajit Hayer-Hartl

**Affiliations:** Department of Cellular Biochemistry, Max Planck Institute of Biochemistry, Martinsried, Germany

**Keywords:** Structural biology, Microbiology

## Abstract

Carboxysomes in cyanobacteria enclose the enzymes Rubisco and carbonic anhydrase to optimize photosynthetic carbon fixation. Understanding carboxysome assembly has implications in agricultural biotechnology. Here we analyzed the role of the scaffolding protein CcmM of the β-cyanobacterium *Synechococcus elongatus* PCC 7942 in sequestrating the hexadecameric Rubisco and the tetrameric carbonic anhydrase, CcaA. We find that the trimeric CcmM, consisting of γCAL oligomerization domains and linked small subunit-like (SSUL) modules, plays a central role in mediation of pre-carboxysome condensate formation through multivalent, cooperative interactions. The γCAL domains interact with the C-terminal tails of the CcaA subunits and additionally mediate a head-to-head association of CcmM trimers. Interestingly, SSUL modules, besides their known function in recruiting Rubisco, also participate in intermolecular interactions with the γCAL domains, providing further valency for network formation. Our findings reveal the mechanism by which CcmM functions as a central organizer of the pre-carboxysome multiprotein matrix, concentrating the core components Rubisco and CcaA before β-carboxysome shell formation.

## Abstract (web summary)

Biochemical, biophysical and structural analysis reveals how the scaffolding protein CcmM recruits the enzymes Rubisco and carbonic anhydrase into a condensate for encapsulation into carboxysomes—microcompartments in cyanobacteria that serve to optimize CO_2_ assimilation.

## Main

Carboxysomes in cyanobacteria are proteinaceous microcompartments that enclose the photosynthetic key enzyme Rubisco (ribulose-1,5-bisphosphate carboxylase/oxygenase), together with carbonic anhydrase (CA), to generate high CO_2_ levels for carbon fixation^1–4^. Implementation of a carboxysome-like CO_2_-concentrating mechanism (CCM) in chloroplasts as a strategy for improving photosynthetic efficiency in crop plants requires a detailed understanding of carboxysome biogenesis^5–15^. Recent advances have shown that, early in this process, specialized scaffolding proteins initiate phase separation of Rubisco into a condensate for subsequent encapsulation^16–19^. However, the mechanisms underlying the sequestration of CA together with Rubisco are not yet understood.

**Fig. 1:**
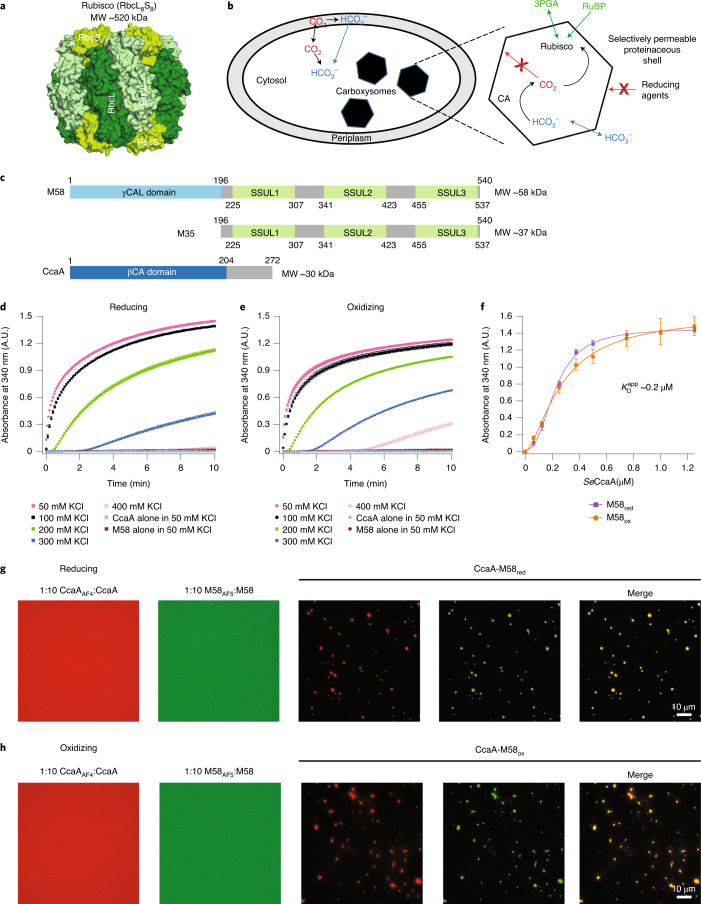
Condensate formation of M58 and CcaA. **a**, Structure of hexadecameric Rubisco in surface representation (PDB: 4RUB). The RbcL_8_ core consists of a tetramer of antiparallel RbcL subunits, shown in two shades of green. The RbcS subunits are shown in light green, four bound on top and four at the bottom of the RbcL_8_ core. **b**, CCM of β-cyanobacteria (see above for details). 3PGA, 3-phosphoglycerate; RuBP, ribulose-1,5-bisphosphate; CA, carbonic anhydrase. **c**, Domain structure of M58, M35 and CcaA from ß-cyanobacterial species *Se*7942. Amino acid numbers and molecular weights are indicated. The SSUL modules of M58 and M35 are numbered SSUL1–3 from the N to the C terminus. **d**,**e**, Salt dependence of condensate formation of CcaA and M58. Purified CcaA (0.5 μM) and M58 (0.5 μM) were incubated in buffer containing 50–400 mM KCl under either reducing (**d**) or oxidizing conditions (**e**) at 25 °C. Condensate formation was monitored by turbidity assay at 340 nm. Proteins alone were analyzed as control. Data are mean ± s.d. of triplicate measurements. **f**, Apparent binding affinity $(K_{\text D}^{\text{app}})$ of CcaA to M58. Turbidity was measured, as above at 0.5 μM, of either reduced M58 (M58_red_) or oxidized M58 (M58_ox_) with 0–1.25 μM CcaA. Absorbance values reached after 10 min are plotted. Data are mean ± s.d. of triplicate measurements. **g**,**h**, Condensate formation analyzed by fluorescence microscopy under reducing (**g**) and oxidizing (**h**) conditions. CcaA (0.25 μM) was mixed with 0.25 μM M58_red_ or M58_ox_. M58 and CcaA were N-terminally labeled with Alexa532 and Alexa405, respectively (M58_AF5_; CcaA_AF4_), and used as 1:10 mixtures with unlabeled protein. Representative data of triplicate measurements are shown. **d**–**f**, Data on which the graphs are based are available as source data. A.U., arbitrary units. Source data

Form I Rubisco, a complex of eight large (RbcL) and eight small (RbcS) subunits (Fig. 1a), evolved in an atmosphere rich in CO_2_. The drop in CO_2_ levels 500 million years ago generated the evolutionary pressure for the development of a CCM in photosynthetic bacteria^20^. There are two forms of carboxysome, α and β, which differ in their components and probably evolved independently^2,21,22^. Their proteinaceous shell allows the entry of dissolved CO_2_ in the form of HCO_3_^−^, which is converted to CO_2_ by CA inside the carboxysome^23^ (Fig. 1b). The shell prevents CO_2_ from diffusing out^24^, resulting in the generation of high levels of CO_2_ in the vicinity of Rubisco^20,22^ and avoiding the competing side-reaction of Rubisco with oxygen (photorespiration)^25^.

β-Carboxysome biogenesis is reliant on the protein CcmM as a central organizing scaffold^26–28^. The full-length CcmM protein in *Synechococcus elongatus* PCC 7942 (*Se*7942) is a homotrimer of ~58-kDa subunits (*Se*M58). The N terminus of each M58 protomer consists of a γ-class carbonic anhydrase-like (γCAL) domain that has lost the functional motifs for CA activity^27,29,30^, followed by three Rubisco small subunit-like (SSUL) modules connected by flexible linkers (Fig. 1c). The γCAL domains mediate M58 trimer formation. A smaller isoform, *Se*M35 (~37 kDa), comprising only SSUL modules (Fig. 1c) is generated from an internal ribosome binding site^31^. In *Se*7942, CA activity is provided by a separate β-class CA protein, CcaA (~30 kDa)^23,32^ (Fig. 1c). Its deletion results in loss of CCM function, with cyanobacterial growth being dependent on high CO_2_ (5%)^33,34^. CcaA is redox regulated and active only in the oxidizing environment of the carboxysome^35^. It is recruited to carboxysomes by the γCAL domains of CcmM^26,27^ by an unknown mechanism.

We recently reported that the SSUL modules of *Se*M35 function to sequester Rubisco into a condensate^16^. This mode of condensate formation, through multivalent interactions of folded domains, differs from the use of intrinsically disordered, linear motifs as phase-separation scaffolds in α-carboxysomes and eukaryotic membraneless compartments^17,36–40^. Here we used a combined structural and biochemical approach to understand the interactions of *Se*M58 with CcaA and Rubisco. Our results reveal multiple, interwoven demixing and coassembly reactions with M58 functioning as a central organizer of the pre-carboxysome matrix. The γCAL domains of trimeric M58 recruit CcaA by binding the C-terminal peptide sequence of CcaA. Moreover, the high local concentration of SSUL modules in trimeric M58 provides enhanced avidity for Rubisco compared to M35. Additionally, SSUL modules engage in dynamic electrostatic interactions with γCAL domains. A head-to-head association of M58 trimers via their γCAL domains further increases local SSUL concentration. These interactions cooperate to facilitate the efficient multiprotein coassembly of CcmM (M58 and M35), CcaA and Rubisco for encapsulation into β-carboxysomes.

## Results

### CcmM–CcaA condensate formation

**Extended Data Fig. 1:**
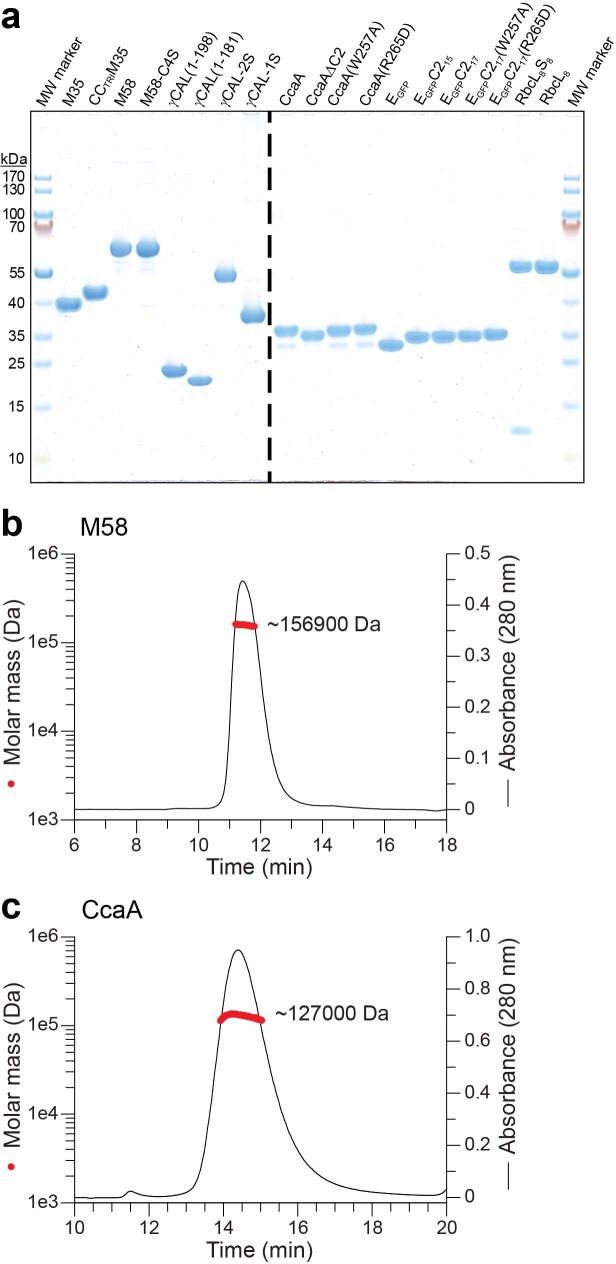
Analysis of purified proteins. **a**, Purified proteins used in this study were analyzed by SDS-PAGE and Coomassie staining. Representative data of two independent experiments are shown. **b-c**, SEC-MALS analysis of purified M58_red_ (b) and CcaA (c). The expected molecular mass of the M58 trimer is 173498.52 Da and of the CcaA tetramer 120741.44 Da. The observed mass values are indicated. M58 was analyzed once, while representative data of three independent measurements is shown for CcaA. Data behind the plots in b and c are available as source data. Source data

**Extended Data Fig. 2:**
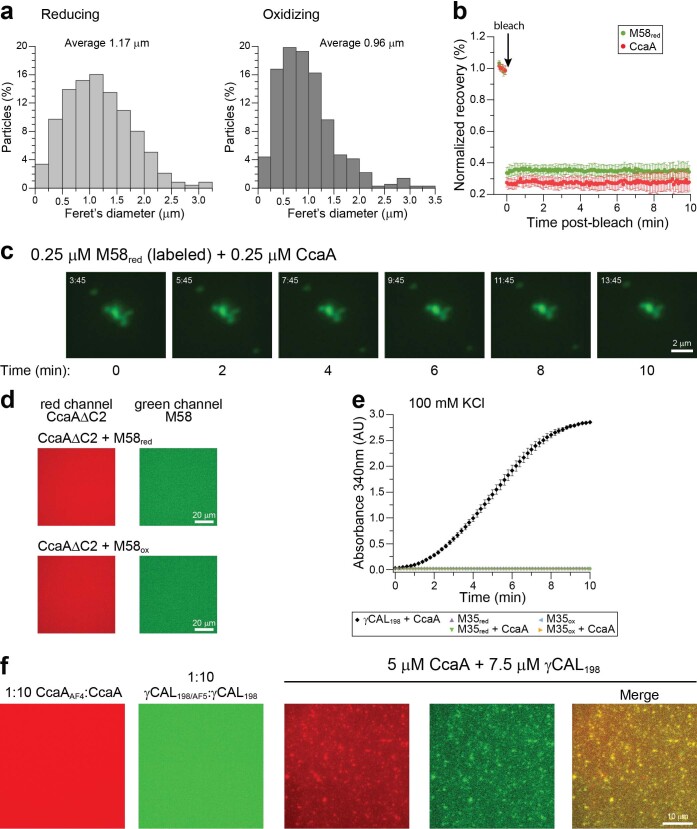
M58-CcaA condensate formation. **a**, Size distribution (Ferret’s diameter) of M58-CcaA condensates formed under reducing (left) and oxidizing conditions (right). 237 and 363 droplet-shaped condensates were analyzed under reducing or oxidizing conditions, respectively. Condensates were generated as in Fig. 1g. **b**, M58 and CcaA in condensates are immobile. Fluorescence recovery after photobleaching (FRAP) experiments were performed on condensates formed by fluorescence labeled M58_red_ (M58_AF5_) with unlabeled CcaA or by unlabeled M58_red_ with labeled CcaA (CcaA_AF4_). Condensates were generated as in Fig. 1g. Pre-bleach fluorescence is set to 1. Mean ± s.d. from n = 20 droplets. **c**, Time-lapse images of droplet fusion. Representative section of Supplementary Video 1 showing absence of fusion of droplets in close proximity from condensates of M58_red_ (0.25 μM) and CcaA (0.25 μM) in the presence of 100 mM KCl. M58_red/AF5_ fluorescence is detected. Scale bar, 2 μm. **d**, The C-terminal C2 sequence of CcaA is required for condensate formation with M58. M58 and CcaAΔC2, fluorescence labeled as in Fig. 1g. (M58_AF5_; CcaAΔC2_AF4_), were used as 1:10 mixtures with unlabeled protein (see Methods). Conditions were as in Fig. 1g,h. Images shown are from a single experiment. **e**, CcaA interacts with the γCAL domain of M58, not with the SSUL modules. Condensate formation was analyzed by turbidity assay as in Fig. 1d,e with 5.0 μM CcaA, 7.5 μM γCAL, 22.5 μM M35_red_/M35_ox_ (100 mM KCl). Data are mean ± s.d. of triplicate measurements. **f**, Condensate formation of CcaA and γCAL analyzed by fluorescence microscopy. N-terminally labeled CcaA (CcaA_AF4_) and γCAL (γCAL_AF5_) were used as 1:10 mixtures with the respective unlabeled proteins and analyzed either alone or in combination at the concentrations indicated. Images shown are from a single experiment. Data behind the graphs in a, b and e are available as source data. Source data

To investigate the interaction of CcmM and CcaA, we recombinantly expressed and purified CcmM (M58 and M35) and the CcaA of *Se*7942 (Extended Data Fig. 1a). Size-exclusion chromatography coupled to multiangle light scattering (SEC–MALS) confirmed that M58 is a trimer in solution while CcaA behaved as a tetramer (Extended Data Fig. 1b,c and Supplementary Table 1), consistent with βCAs functioning as dimers or higher-order oligomers^26,41–45^. We performed turbidity assays to monitor the interaction between M58 and CcaA. While no turbidity was detected for either protein alone (Fig. 1d,e), a strong turbidity signal was observed when M58 and CcaA were combined (Fig. 1d–f), consistent with condensate formation. Turbidity developed at a similar rate independent of the redox state of the M58 SSUL modules (*t*_1/2_ = ~0.6 min at 100 mM KCl) (Fig. 1d,e) with an apparent affinity $(K_{\text{D}}^{\text{app}})$ of CcaA for M58 of ~0.2 μM (Fig. 1f). The M58-CcaA interaction was impaired by high salt (Fig. 1d,e), indicating the involvement of electrostatic forces. Light microscopy of M58 or CcaA labeled N-terminally with fluorophores Alexa532 (M58_AF5_) and Alexa405 (CcaA_AF4_), and mixed 1:10 with the respective unlabeled protein, showed a diffuse distribution (Fig. 1g,h). When the two proteins were combined at equimolar concentration (0.25 μM), coassembly into fluorescent condensates with an average Feret’s diameter of ~1.0 μm was observed (Fig. 1g,h and Extended Data Fig. 2a). Fluorescence recovery after photobleaching (FRAP) experiments showed no recovery of fluorescence signal in either channel, indicating a strong association between M58 and CcaA (Extended Data Fig. 2b). No fusion of droplets was observed over a period of 20 min (Extended Data Fig. 2c and Supplementary Video 1), consistent with their low liquidity^46^ as indicated by FRAP.

In summary, condensate formation mediated by M58 provides a plausible mechanism for CcaA sequestration and recruitment into carboxysomes.

### CcaA engages the γCAL domain of M58

**Fig. 2:**
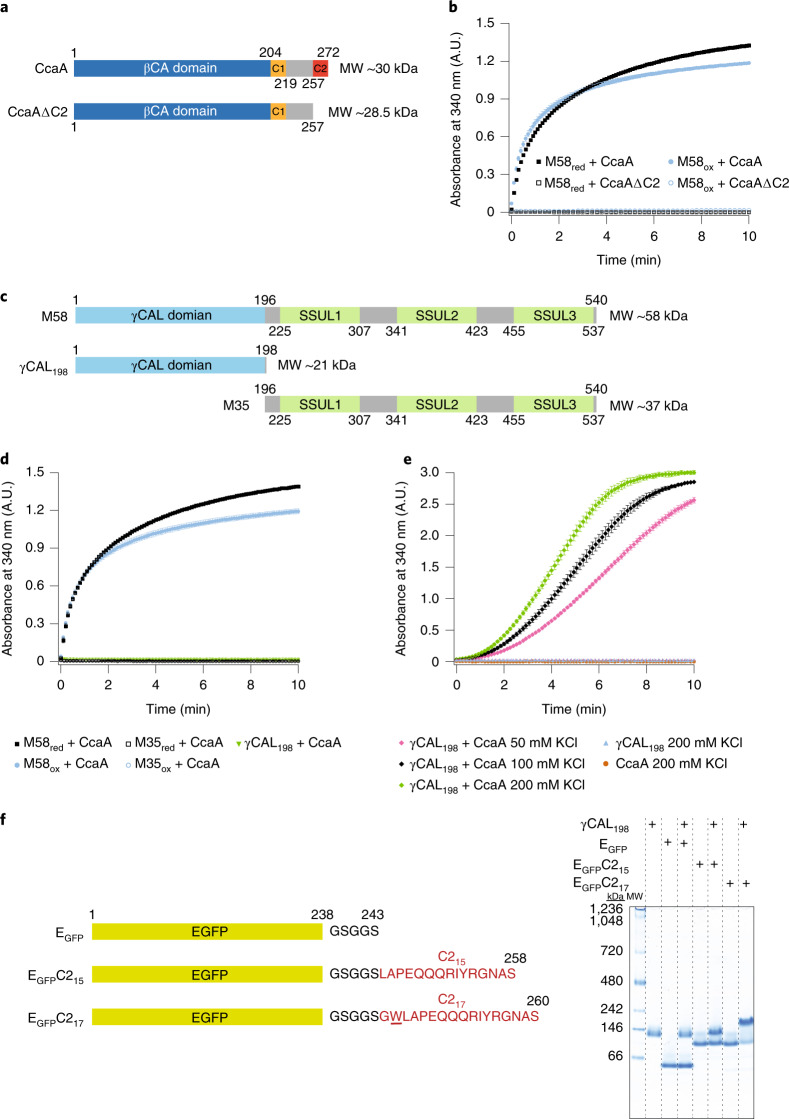
Structural requirements for M58–CcaA condensate formation. **a**,**b**, Condensate formation is dependent on the C-terminal C2 peptide sequence of CcaA. **a**, Domain structure of CcaA and CcaAΔC2, lacking the C-terminal 15 residues of CcaA. Amino acid numbers are indicated. **b**, Turbidity assays as in Fig. 1d,e at 100 mM KCl with 0.5 μM CcaA or CcaAΔC2 and 0.5 μM M58_red_ or M58_ox_. Data are mean ± s.d. of triplicate measurements. **c**, Domain structure of M58, γCAL_198_ and M35 constructs. Amino acid numbers are indicated. The SSUL modules of M58 and M35 are numbered SSUL1–3 from the N to the C terminus. **d**, Condensate formation requires the γCAL domains and SSUL modules of M58. Turbidity assays with the combinations of proteins indicated were performed at 100 mM KCl using 0.75 μM γCAL_198_, 0.5 μM CcaA, 0.5 μM M58_red_/M58_ox_ and 2.25 μM M35. Note that the relative concentrations of M58 and M35 were adjusted to maintain SSUL modules at a similar concentration. Data are mean ± s.d. of triplicate measurements. **e**, Condensate formation of γCAL_198_ and CcaA requires high protein concentrations and the presence of salt. Turbidity assays with the combinations of proteins indicated were performed at 50–200 mM KCl using 5.0 μM CcaA and 7.5 μM γCAL_198_. Data are mean ± s.d. of triplicate measurements. **f**, The C2_17_ sequence of CcaA is sufficient for CcaA binding to γCAL_198_. Left: E_GFP_C2 constructs containing either the C-terminal 15 or 17 residues of CcaA fused to eGFP. Right: purified proteins (22.5 μM) were incubated with γCAL_198_ (7.5 μM) (100 mM KCl) for 15 min at 25 °C, followed by analysis of complex formation by native-PAGE and Coomassie staining. Representative data of triplicate experiments are shown. **b**,**d**,**e**, Data are available as Source data. Source data

The CcaA protein consists of an N-terminal βCA domain followed by a hydrophilic and intrinsically disordered C-terminal sequence of 60–70 residues^2,23^. This sequence contains two functionally important regions of ~15 residues, C1 and C2, separated by an unstructured, hydrophilic sequence of ~40–50 amino acids^41,47^ (Fig. 2a). C1 has been shown to be required for oligomerization and CcaA activity^47^, while the function of C2 remains unclear^28,41^. To understand the function of C2, we generated a CcaA mutant lacking the last 15 residues (CcaAΔC2) (Fig. 2a, Extended Data Fig. 1a and Supplementary Table 1). Notably, no turbidity was observed upon mixing CcaAΔC2 and M58 (Fig. 2b) and no fluorescent condensates formed (Extended Data Fig. 2d), suggesting that the C2 sequence mediates the interaction of CcaA with the γCAL domains of M58 (ref. ^27^).

To confirm this interaction, we recombinantly expressed and purified the γCAL domain of M58 (residues 1–198) (γCAL_198_) (Fig. 2c, Extended Data Fig. 1a and Supplementary Table 1). As expected, CcaA did not interact with M35, which lacks the γCAL domain, as monitored by turbidity assay (Fig. 2c,d). CcaA at a concentration of 0.5 μM also failed to interact detectably with the trimeric γCAL_198_ (0.75 μM) (Fig. 2d). However, increasing the concentration of CcaA and γCAL_198_ by tenfold resulted in the development of turbidity with slow kinetics (*t*_1/2_ = ~5 min) (Fig. 2e), while no interaction was observed with M35 (Extended Data Fig. 2e). Interestingly, unlike the salt-sensitive M58-CcaA interaction (Fig. 1d,e), complex formation of CcaA with γCAL_198_ was somewhat enhanced at higher salt concentration (Fig. 2e), suggesting a contribution of hydrophobic forces. Fluorescence microscopy revealed small CcaA–γCAL condensates on a background of diffusely distributed proteins (Extended Data Fig. 2f).

To test whether the C2 region of CcaA was sufficient to mediate binding to γCAL_198_, we attached the 15-residue C2 sequence (LAPEQQQRIYRGNAS) to enhanced green fluorescent protein (E_GFP_) via a short flexible linker (GSGGS) (E_GFP_C2_15_) (Fig. 2f). No complex formation of E_GFP_C2_15_ with γCAL_198_ was detected following analysis by native polyacrylamide gel electrophoresis (PAGE) (Fig. 2f). However, we noticed the presence of a tryptophan residue just N-terminal of the C2 sequence, which would increase the hydrophobicity of the sequence. Indeed, a GFP construct containing the last 17 residues of CcaA (GWLAPEQQQRIYRGNAS) (E_GFP_C2_17_) readily bound to γCAL_198_ (Fig. 2f).

In summary, the C-terminal C2 sequence of CcaA interacts with the γCAL domains of M58, an interaction critical for the recruitment of CcaA into the pre-carboxysome.

### Structural basis of the CcaA C2-γCAL interaction

**Fig. 3:**
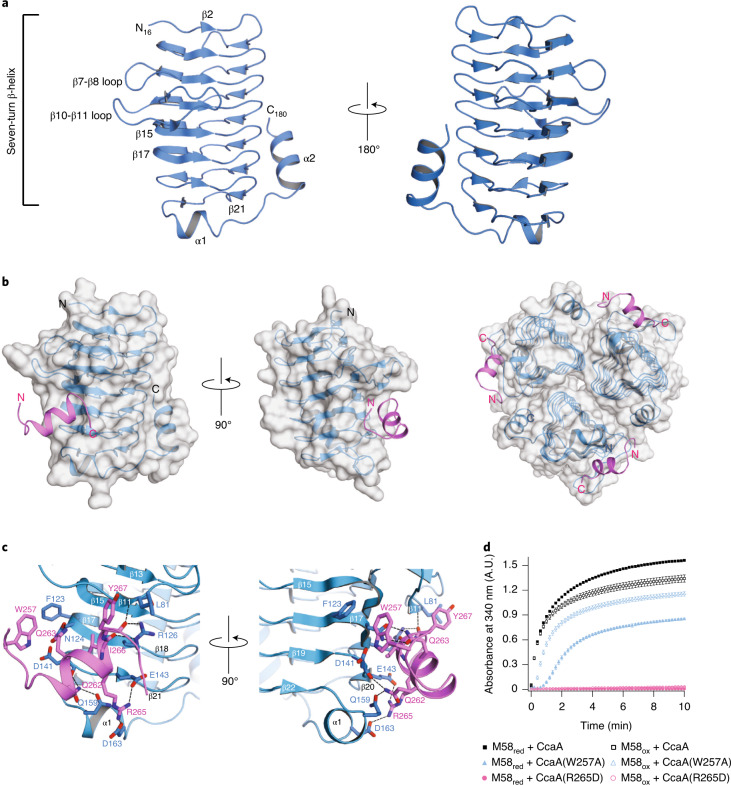
Structure of the γCAL–C2 complex. **a**, Structural model of the *Se*7942 γCAL domain (residues 1–181) at 1.67-Å resolution, in ribbon representation. Secondary structural elements are indicated. **b**, Structural model of the γCAL_181_–C2_17_ complex at 1.63-Å resolution. Left: γCAL domain (blue) is shown in transparent surface representation and the bound helical C2_17_ peptide (pink) in ribbon representation. Right: model of the trimer of the γCAL_181_–C2_17_ complex in transparent surface representation. **c**, Details of the interaction of C2 peptide with γCAL. Proteins are colored as in **b** and shown in ribbon representation, with critical amino acid residues in stick representation. Secondary structural elements and residue numbers are indicated. **d**, Point mutations of C2_17_ peptide residues forming the interface with γCAL reduced or abolished the interaction between CcaA and M58. Turbidity assays with the combinations of proteins indicated were performed at 100 mM KCl using either 0.5 μM M58_red_/M58_ox_ and 0.5 μM CcaA or the mutant proteins CcaA(W257A) or CcaA(R265D). Data are mean ± s.d. of triplicate measurements. **d**, Data are available as Source data. Source data

**Extended Data Fig. 3:**
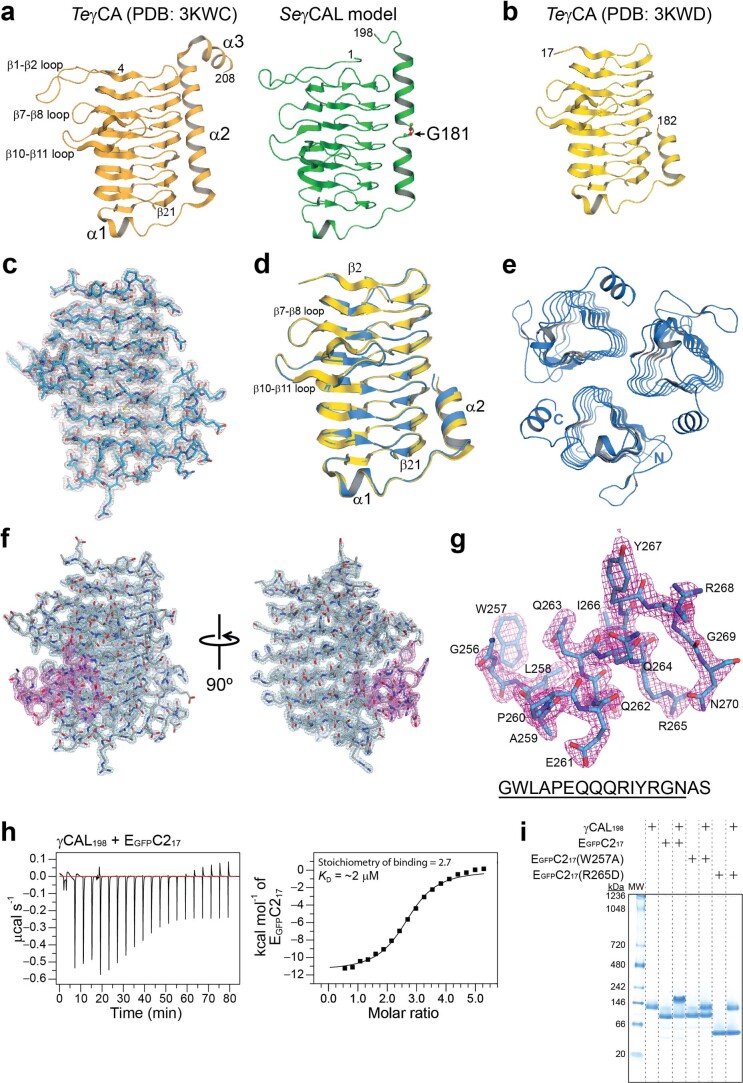
Structure of the γCAL domain with and without bound C2 peptide. **a**, Left: Crystal structure of the γCA domain of CcmM from *Thermosynechococcus elongatus* BP-1 (residues 1-209, PDB: 3KWC)^29^ in ribbon representation. Right: Model of the structure of the *Se*γCAL (1-198) generated with Phyre2^84^. The putative break in the α2 helix is indicated. **b**, Structure of the C-terminally truncated form of *Te*γCA (residues 1-193, PDB: 3KWD) with residues after the break in α2 unresolved. **c**, Experimental electron density for the *Se*γCAL_181_ protomer (Table 1). The weighted 2Fo–Fc density map at 1.5 σ is shown as gray meshwork with final model in stick representation. **d**, Overlay of the structures of the γCAL protomer (blue) and of *Te*γCA (PDB: 3KWD) (yellow). **e**, Structural model of the *Se*γCAL_181_ trimer viewed along the long axis of the protomer β-helices. **f**, Experimental electron density for the *Se*γCAL_181_-C2_17_ complex (Table 1). The weighted 2Fo–Fc density map at 1.5 σ is shown for γCAL (gray), the bound C2_17_ peptide (magenta) as meshwork, and final model in stick representation. **g**, The weighted 2Fo–Fc electron density map at 1.5 σ for the 15 residues of the C2_17_ peptide (underlined in the sequence) resolved in the structure of the complex. **h**, Isothermal titration calorimetry (ITC) of the interaction of E_GFP_C2_17_ with γCAL_198_. Left: Change in enthalpy between sample and reference buffer upon titration of E_GFP_C2_17_ into buffer containing γCAL_198_. Right: Fitted integrated data generated in Origin. Representative data of two independent experiments are shown. i, Point mutations of C2_17_ residues forming the interface with γCAL. Analysis of complex formation by native-PAGE and Coomassie staining of EGFP-C2_17_ and mutant proteins, E_GFP_-C2_17_(W257A) and E_GFP_-C2_17_(R265D), with γCAL_198_. The purified proteins (22.5 μM) were incubated with γCAL_198_ (7.5 μM) (100 mM KCl) for 15 min at 25 ^o^C. Representative data of three independent experiments are shown.

To identify the C2 binding site on γCAL, we analyzed the γCAL_198_–C2_17_ complex by X-ray crystallography. However, crystals of the complex diffracted to only ~3.5-Å resolution, presumably due to the presence of a break in helix α2 of γCAL at residue 181, as suggested by crystal structures of the γCA domain of *Thermosynechococcus elongatus* BP-1 (PDB: 3KWC; PDB: 3KWD)^29^ (Extended Data Fig. 3a,b). Indeed, *Se*γCAL 1–181 (γCAL_181_) (Extended Data Fig. 1a) produced well-diffracting crystals, allowing structure solution by molecular replacement (PDB: 3KWC) at 1.67-Å resolution (Fig. 3a, Table 1 and Extended Data Fig. 3c). The overall structure of the γCAL_181_ protomer is highly similar to that of *Te*γCA (PDB: 3KWD) (Cα root mean square deviation (RMSD), 0.48 Å) (Extended Data Fig. 3d): it consists of an N-terminal, seven-turn, left-handed β-helix followed by a short helix α1, a long linker and part of helix α2, which packs along one face of the β-helix (Fig. 3a). The asymmetric unit of the crystal contained the protomer of γCAL_181_, while γCAL exists as a trimer in solution (Supplementary Table 1). Indeed, analysis using proteins, interfaces, structures and assemblies (PISA)^48^ indicated an extensive interface between subunits (4,180 Å^2^ buried at the interface from a total accessible surface of 18,860 Å^2^) (Extended Data Fig. 3e).

**Table 1:** Data collection and refinement statistics

	*Se*γCAL_181_*	*Se*γCAL_181_–C2_17_ complex*
**Data collection**		
Space group	*H*32	*H*32
Cell dimensions		
*a*, *b*, *c* (Å)	89.74, 89.74, 130.79	89.36, 89.36, 129.84
α, β, γ (°)	90.0, 90.0, 120.0	90.0, 90.0, 120.0
Resolution (Å)	66.81–1.67 (1.70–1.67)**	37.08–1.63 (1.66–1.63)
*R*_merge_	0.081 (0.960)	0.030 (0.684)
*I*/σ*I*	13.8 (2.3)	45.4 (2.2)
*CC*_1/2_	0.998 (0.823)	1.000 (0.865)
Completeness (%)	99.9 (100.0)	98.8 (91.3)
Redundancy	9.7 (10.0)	18.4 (9.2)
**Refinement**		
Resolution (Å)	66.81–1.67	37.08–1.63
No. reflections	22,708	23,554
*R*_work_ / *R*_free_	0.164/0.200	0.177/0.200
No. atoms		
Protein	1,248	1,376
Ligand/ion	2	2
Water	127	103
*B* factors		
Protein	30	37
Ligand/ion	32	36
Water	42	44
R.m.s. deviations		
Bond lengths (Å)	0.015	0.017
Bond angles (°)	1.95	2.10

*One crystal per structure.

**Values in parentheses are for highest-resolution shell.

We also solved the structure of γCAL_181_ in complex with the C2_17_ peptide of CcaA at a resolution of 1.63 Å (Fig. 3b, Table 1 and Extended Data Fig. 3f,g). The asymmetric unit of the crystal contained the protomer of γCAL_181_ with one peptide bound (Fig. 3b and Extended Data Fig. 3f). This indicates that all binding sites of the trimeric γCAL_181_ are occupied by peptide, providing the basis for multivalent network formation between M58 and CcaA (Fig. 3b). Analysis by isothermal titration calorimetry (ITC) using the monomeric E_GFP_C2_17_ revealed a binding affinity (*K*_*D*_) of ~2 μM at a stoichiometry of ~2.7 per γCAL_198_ trimer (Extended Data Fig. 3h). The peptide is bound as a two-turn α-helix (residues PEQQQRIY) in a pocket beneath the protruding β10-β11 loop, making extensive interactions with residues in β11, β17, the β19-β20 loop and α1 of the γCAL protomer. The hydrophobic interactions include π-stacking between the indole ring of residue W257 in the C2 peptide with the benzene ring of F123 (β17) in γCAL (Fig. 3c). In addition, the hydrophobic residue Y267 of C2 contacts the γCAL residue L81 (β11) (Fig. 3c). Two electrostatic interactions (salt bridges) are formed by the guanidinium group of the peptide residue R265 with E143 (β20) and D163 (α1) of γCAL (Fig. 3c). The side chains of C2 residues Q262 and Q263 form hydrogen bonds with the backbone of D141 (β19-β20 loop) and the side chain of N124 (β17) in γCAL, respectively. The side chain of C2 residue Q262 also forms a hydrogen bond with the side chain of γCAL residue Q159 (α1). Moreover, the guanidinium group of R126 (β17) in γCAL forms hydrogen bonds with the backbone of I266 and Y267 in C2, and another hydrogen bond is formed between the backbone of C2 residue Y267 and L81 (β11) of γCAL (Fig. 3c). Notably, the binding site of C2 is highly conserved in the γCA/γCAL domains of CcmM proteins.

To validate the contribution of residues W257 and R265 to the C2–γCAL interface, we generated the mutants W257A and R265D in E_GFP_C2_17_ and CcaA. Gel shift assays showed that both mutant proteins failed to form a complex with trimeric γCAL (Extended Data Fig. 3i). M58–CcaA condensate formation monitored by turbidity assay was reduced by ~50% for CcaA(W257A) and abolished for CcaA(R265D) (Fig. 3d). These results underscore the specific contribution of hydrophobic and charge interactions forming the CcaA–M58 network.

In summary, assuming the absence of steric hindrance, the γCAL trimer in the context of M58 may interact with two or three CcaA tetramers via their C2 sequences as the basis for condensate formation.

### Contribution of SSUL modules to M58-CcaA interaction

**Fig. 4:**
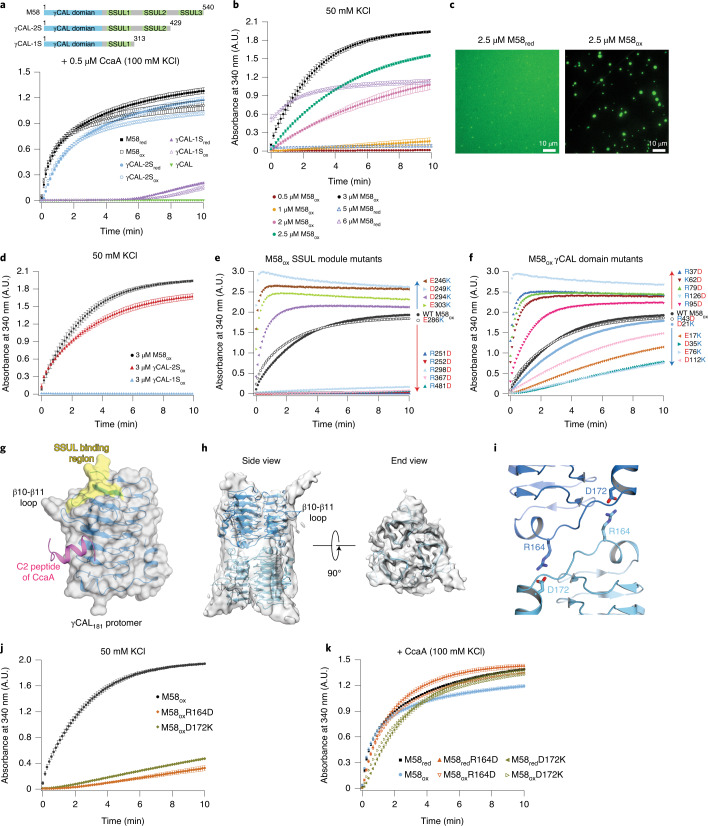
Association between SSUL modules and γCAL domains of M58. **a**, Top: schematic representation of wild-type M58 and truncation mutants containing two or one SSUL modules (γCAL-2S and γCAL-1S, respectively). Residue numbers are indicated. Bottom: contribution of SSUL modules to M58–CcaA condensate formation. Turbidity was monitored over time after mixing CcaA (0.5 μM) with the reduced and oxidized wild-type M58 or truncated M58 constructs (0.5 μM) indicated at 100 mM KCl. Data are mean ± s.d. of triplicate measurements. **b**, Concentration dependence of M58 homocondensate formation. Turbidity assays were performed following dilution of M58_ox_ from high salt (500 mM KCl) to reach final protein concentrations of 0.5–3.0 μM and a salt concentration of 50 mM KCl. M58_red_ (5 and 6 μM) was also analyzed. Data are mean ± s.d. of triplicate measurements. **c**, M58 homocondensate formation is strongly enhanced under oxidizing conditions. M58_ox_ and M58_red_ (2.5 μM) homodemixing was analyzed by fluorescence microscopy as in Fig. 1g,h. M58 was N-terminally labeled with fluorophore Alexa532 (M58_AF5_) and used as a 1:10 mixture with unlabeled protein. Representative data of triplicate experiments are shown. **d**, M58_ox_ homocondensate formation is mediated by the SSUL modules of M58. Turbidity assays were performed for reactions containing M58_ox_, γCAL-2S_ox_ and γCAL-1S_ox_ (3 μM each at 50 mM KCl). Data are mean ± s.d. of triplicate measurements. **e**, Mutation of charged residues in SSUL1, SSUL2 or SSUL3 inhibit or enhance M58_ox_ homocondensate formation, measured by turbidity assay as in **d**. Mutations of negative to positive (D/E to K, blue triangles) enhance the interaction while mutations of positive to negative (R to D, red triangles) are inhibitory. Numbers of mutated residues are indicated. Representative data of two independent measurements are shown. **f**, Point mutations of charged residues in the γCAL domain reduce or enhance M58_ox_ homocondensate formation, measured by turbidity assay as in **e**. Mutations of positive to negative (K/R to D, red triangles) enhanced the interaction and mutations of negative to positive (D/E to K, blue triangles) were inhibitory. Numbers of mutated residues are indicated. Representative data of two independent measurements are shown. **g**, Putative binding region of the SSUL module (yellow) mapped on the γCAL_181_–C2 protomer (PDB: 7O54). **h**, Head-to-head association of M58 trimers. The structural model of the dimer of γCAL trimers from the crystal structure was fitted into the cryo-EM density map from the M58_ox_ condensate. The structure is shown in ribbon representation. Additional densities at the corners probably represent SSUL modules that associate in a dynamic manner. The cryo-EM density map was low-pass filtered to 5 Å to show these additional densities. **i**, Detail of the structural model from the crystal structure of *Se*γCAL_181_ showing the two salt bridges connecting protomers of opposing trimers. The side chains of residues R164 and D172 forming critical salt bridges are shown in stick representation. **j**, Head-to-head association of M58 trimers is required for efficient M58_ox_ homocondensate formation. M58_ox_ and mutants M58_ox_R164D and M58_ox_D172K, disrupting the salt bridges between M58 trimers, were analyzed by turbidity assay as in **d** at 3 μM final protein concentration (50 mM KCl). Data are mean ± s.d. of triplicate measurements. **k**, Head-to-head association of M58 trimers is not required for M58–CcaA condensate formation. M58 and mutants M58(R164D) and M58(D172K) (0.5 μM) were incubated with CcaA (0.5 μM) under reducing and oxidizing conditions, and condensate formation analyzed by turbidity assay at 100 mM KCl. Data are mean ± s.d. of triplicate measurements. **a**,**b**,**d**–**f**,**j**,**k**, Data are available as source data. Source data

So far, our analysis had shown that the interaction between M58 and CcaA involves two components: (1) a salt-sensitive component detected with full-length CcmM (M58) and CcaA, and (2) interaction between the γCAL domain and the C2 peptide of CcaA, which has a notable hydrophobic component. To understand the salt-sensitive component in more detail, we investigated the interaction of CcaA with C-terminal truncation mutants of M58 containing either two (γCAL-2S) or one (γCAL-1S) SSUL modules (Fig. 4a and Supplementary Table 1). Condensate formation with CcaA was only mildly impaired with γCAL-2S but was strongly reduced with γCAL-1S (Fig. 4a), indicating that SSUL modules provide additional, critical valency for condensate formation. Since no binding of CcaA to M35 was observed (Fig. 2d and Extended Data Fig. 2e), this raised the question of how SSUL modules contribute to CcaA–M58 complex formation. Might the SSUL modules mediate homo-oligomeric interactions between M58 trimers?

**Extended Data Fig. 4:**
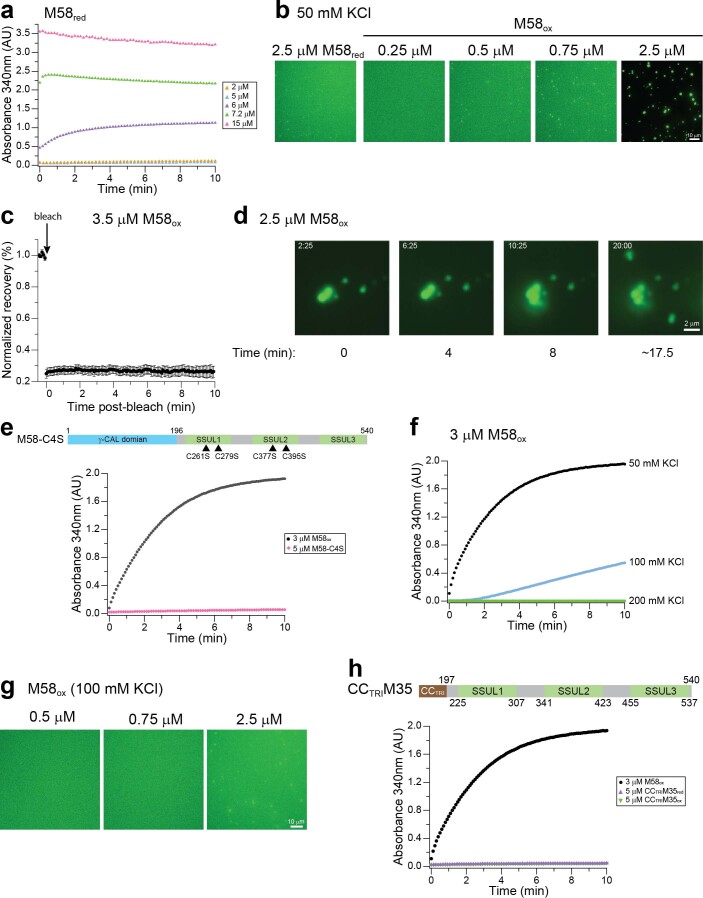
Role of SSUL modules in M58 homo-condensate formation. **a**, Concentration dependence of M58_red_ homo-condensate formation. Turbidity assays were performed with M58_red_ at the concentrations indicated at 50 mM KCl as in Fig. 4b. Representative data of two independent measurements are shown. **b**, Oxidizing conditions enhance M58 homo-dexing. Concentration dependence of condensate formation analyzed by fluorescence microscopy as in Fig. 4c. M58_red_ at 2.5 μM is shown as control. Representative data of two independent experiments are shown. **c**, M58_ox_ condensates are immobile. FRAP experiments on condensates formed using 3.5 μM M58_ox_. Pre-bleach fluorescence is set to 1. Mean ± s.d. from n = 20 droplets. **d**, Time-lapse images of droplet fusion. Representative section of Supplementary Video 2 showing absence of fusion of juxtaposed droplets of M58_ox_ (2.5 μM; 50 mM KCl). **e**, Requirement of disulfide bond formation in SSUL for M58 homo-demixing. Top: Domain structure of M58 indicating the position of cysteine mutations in SSUL1 and SSUL2. Bottom: M58_ox_ (3 μM) and M58-C4S (5 μM) analyzed by turbidity assay (50 mM KCl). Representative data of two independent experiments are shown. **f**, Salt dependence of M58_ox_ homo-demixing. Turbidity assays performed with 3 μM M58_ox_ at 50 to 200 mM KCl. Representative data of two independent experiments are shown. **g**, M58_ox_ homo-demixing analyzed by fluorescence microscopy as in (b) at 100 mM KCl. Representative data of two independent experiments are shown. **h**, M58 homo-demixing requires the γCAL domains of M58. Top: Domain structure of CC_TRI_M35. Bottom: Turbidity assays with 3 μM M58_ox_, 5 μM CCTRIM35_red_ and 5 μM CC_TRI_M35_ox_ at 50 mM KCl. Representative data of two independent experiments are shown. Data behind the graphs in a, c, e, f and h are available as source data. Source data

To address this possibility, we analyzed whether M58 can undergo condensate formation on its own. Reasoning that interactions involving SSUL modules might be more salt sensitive^16^, we conducted this analysis at a reduced salt concentration (50 mM KCl) and at a higher protein concentration compared to the experiments above (0.5 μM M58 in Fig. 1d,e). Interestingly, we observed homodemixing of both reduced and oxidized M58 (M58_red_ and M58_ox_, respectively), with M58_red_ requiring somewhat higher concentrations (Fig. 4b and Extended Data Fig. 4a). Fluorescence microscopy showed that condensate formation by M58_ox_ was enhanced compared to M58_red_ (Fig. 4c and Extended Data Fig. 4b). As in the case of the M58–CcaA condensate, there was no measurable recovery by FRAP (Extended Data Fig. 4c) and no droplet fusion (Extended Data Fig. 4d and Supplementary Video 2). Notably, mutation of the disulfide-forming cysteines in SSUL1 and SSUL2 to serine prohibited homodemixing (Extended Data Fig. 4e), consistent with the redox dependence of the process.

The redox dependence of M58 homodemixing suggested involvement of SSUL modules in mediation of homotypic interactions. Indeed, homodemixing proved to be highly salt sensitive (Extended Data Fig. 4f,g), which might explain the salt-sensitive component of the CcaA-M58 interaction. Indeed, while removal of one SSUL module from M58 (γCAL-2S) had only a minor effect, removal of two SSUL modules (γCAL-1S) completely abolished M58_ox_ homodemixing (Fig. 4d), suggesting that SSUL modules play a role in mediation of the interaction between M58 trimers. Since M35 on its own did not undergo phase separation (Extended Data Fig. 2e), such interactions would have to be specific for trimeric M58. To test this, we engineered a M35 trimer by fusing a trimeric coiled-coil sequence^49^ to the M35 N terminus (CC_TRI_M35) (Extended Data Fig. 4h and Supplementary Table 1). However, no turbidity signal was detectable with CC_TRI_M35, even at high concentrations and low salt (Extended Data Fig. 4h), pointing to an interaction of SSUL modules with the γCAL domains in M58 and not between SSUL modules.

In summary, SSUL modules contribute to M58–CcaA condensate formation, apparently by mediation of salt-sensitive intermolecular interactions with the γCAL domains of neighboring M58 trimers, which allow M58 homodemixing.

### Charge-charge interactions between SSUL and γCAL

**Extended Data Fig. 5:**
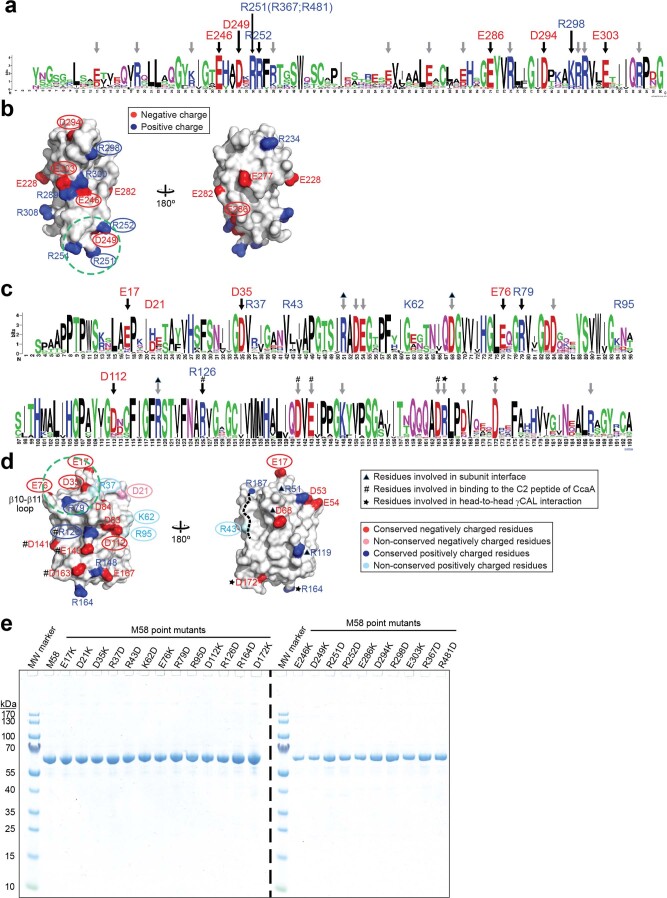
Conservation of charged surface residues of SSUL and γCAL. **a**, Homology analysis of SSUL sequences. 500 different sequences of SSUL modules were analyzed using ConSurf^80^. Conserved charged residues are indicated by arrows. The black arrows indicate residues that were mutated. Note that the conserved residues E271, K298 and R299 are Q271, R298 and S299 in SSUL1, Q387, R414 and S415 in SSUL2 and T501, R528 and R529 in SSUL3 of *Se*7942. **b**, Conserved charged residues mapped on the SSUL structure (PDB: 6HBB) shown in surface representation. Mutated residues are circled. Dashed green circle indicates the putative interacting region of SSUL for the γCAL domains of M58. **c**, Homology analysis of γCA/γCAL sequences of CcmM. 150 different sequences were analyzed using ConSurf^80^. Conserved charged residues are indicated by arrows. The black arrows indicate residues that were mutated. Residues involved in the subunit interface, in binding to the C-terminus of CcaA or involved in the head-to-head association of γCAL trimers are indicated. **d**, Conserved charged residues mapped on the γCAL structure (PDB: 7O4Z) shown in surface representation. Mutated residues are circled. Dashed green circle indicates the putative binding region of SSUL on the γCAL domain of M58. **e**, Purified M58 and mutants used in Fig. 4 were analyzed by SDS-PAGE and Coomassie staining. Representative data of two independent experiments are shown.

Both the SSUL and γCAL domains expose multiple charged residues, which are characterized by a high degree of conservation (Extended Data Fig. 5a–d). Individual mutations of arginine residues 251, 252 or 298 in SSUL1, or R367 in SSUL2 or R481 in SSUL3 to aspartate essentially abolished homodemixing of M58_ox_ (Fig. 4e and Extended Data Fig. 5e), indicating that all three SSUL modules contribute. Moreover, individual mutations of the conserved negatively charged residues E246, D249, D294 and E303 in SSUL1 to lysine resulted in enhanced turbidity, except for the mutant E286K, which behaved like wild type (Fig. 4e). These results suggested that a region of SSUL with several exposed positively charged residues would promote interaction with the γCAL domains of M58. One good candidate was the area containing residues R251, R252 and R254 (Extended Data Fig. 5b). Indeed, point mutation of the spatially close, negatively charged residue D249 to lysine enhanced M58 homodemixing (Fig. 4e and Extended Data Fig. 5b). Interestingly, this region of SSUL is also involved in the interaction of the SSUL modules with Rubisco^16^.

To identify the complementary surface of the γCAL domain that may interact with SSUL, we individually mutated the negatively charged γCAL residues E17, D21, D35, E76 or D112 to lysine, and the positively charged residues R37, R43, K62, R79, R95 or R126 to aspartate (Extended Data Fig. 5e). Among these residues, E17, D35, E76, R79, D112 and R126 are relatively conserved (Extended Data Fig. 5c). As expected, the effect of these charge mutations was reversed (Fig. 4f) compared to the mutations in SSUL (Fig. 4e)—converting positive charges to negative on γCAL enhanced M58_ox_ homodemixing (except for mutant R43D) while changing negative charges to positive reduced condensate formation (except for mutant D21K) (Fig. 4f and Extended Data Fig. 5d). Note that the mutations D21K and R43D, which essentially preserved wild-type binding, are located at the edge of the putative surface for γCAL trimer formation and are also not highly conserved (Fig. 4f and Extended Data Fig. 5c,d). Mutational analysis suggests that negatively charged residues E17, D35 and E76, spatially located above the β10-β11 loop of the γCAL domain, form the intermolecular interaction site for SSUL modules (Extended Data Fig. 5d). Indeed, point mutation of the nearby positively charged residues, R37 and R79, to aspartate strongly promoted M58 homodemixing (Fig. 4f). Interestingly, it appears that the binding region on the γCAL domain for SSUL does not overlap with the site for binding the C2 peptide of CcaA, which is located below the β10-β11 loop (Fig. 4g). This is consistent with the observation that both SSUL modules and CcaA binding via the C2 peptide are required for efficient formation of the M58–CcaA condensate (Fig. 4a). Note that the trimeric state of M58 was maintained for all SSUL and γCAL domain mutants (Supplementary Table 1).

In summary, the intermolecular interaction between SSUL modules and the γCAL domains of M58 trimers involves a complex interplay of attractive and repulsive forces, consistent with single-charge reversal mutations having reducing or enhancing effects (Fig. 4e,f).

### Head-to-head association of γCAL trimers

**Extended Data Fig. 6:**
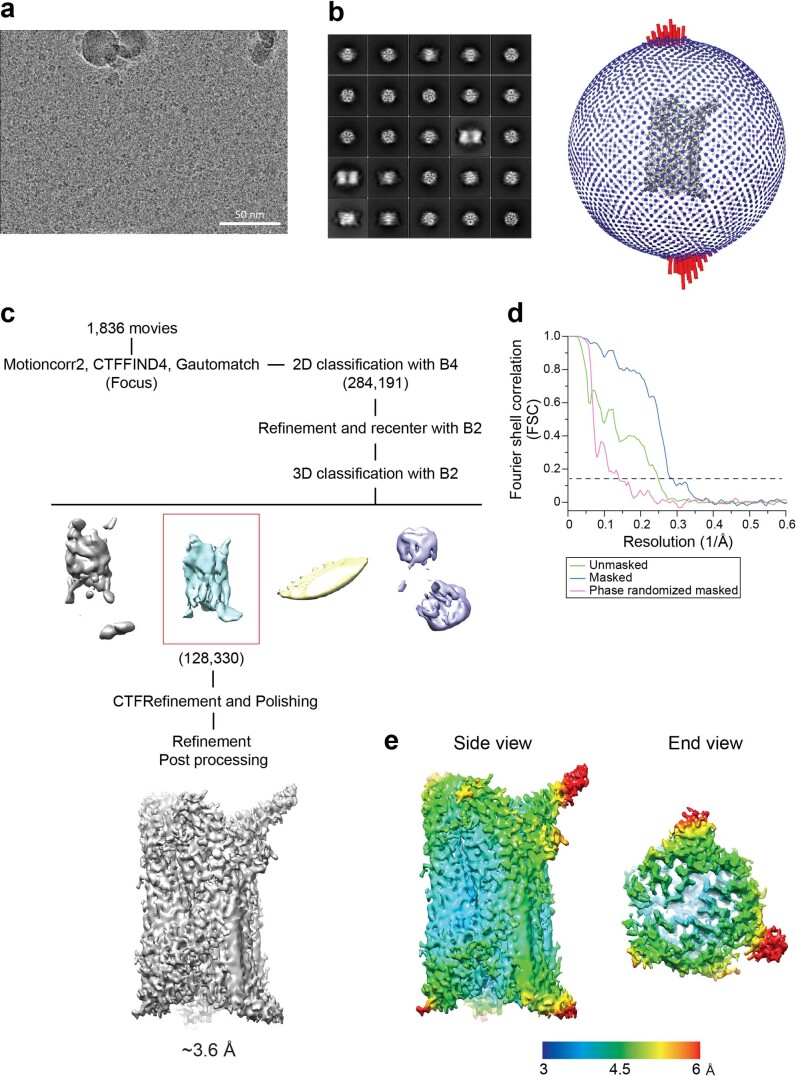
Cryo-EM single-particle reconstruction of M58 in M58 homo-condensates. **a**, A representative micrograph of the M58_ox_ homo-condensate formed as in Fig. 4c. Scale bar, 50 nm. Representative micrograph of a total of 1,836 is shown. **b**, 2D class averages of particles in (a) (left) and the particle angular distribution plot (right) showing a preferred particle orientation in end-view. **c**, The single-particle data processing workflow. Particle numbers are indicated in parentheses. B4, 4 × 4 pixel-binned image. B2, 2 × 2 pixel-binned image. See Methods for details. **d**, Gold-standard FSC curves (EMDB: EMD-12730). e, Local resolution maps of side and end views. The color gradient from blue to red indicates local resolution from 3.0 to 6.0 Å.

To analyze the structural basis of the intermolecular interactions of M58, we performed cryo-EM of M58_ox_. Reference-free, two-dimensional (2D) class averages revealed a class of barrel-shaped complexes with dimensions of ~5 × 8.2 nm^2^, consistent with two-stacked γCAL trimers in side view (Extended Data Fig. 6a,b). Notably, a head-to-head association of γCA trimeric domains is present in the asymmetric unit of the *Te*γCA crystal (PDB: 3KWC)^29^, and such an interaction is also observed in the molecular packing of our γCAL_181_ and γCAL_181_–C2_17_ crystals. A three-dimensional (3D) classification without imposed symmetry resulted in an EM density map of ~3.6-Å resolution (Fig. 4h, Table 2 and Extended Data Fig. 6).

**Table 2:** Cryo-EM data collection and map resolution

	*Se*M58_ox_ (EMDB-12730)	*Se*M58_red_-*Se*Rubisco (EMDB-12731)	*Se*M58_red_-*Se*RbcL_8_ (EMDB-12732)
**Data collection and processing**			
Magnification	215,000	22,000	22,000
Voltage (kV)	300	200	200
Electron exposure (e^–^/Å^2^)	60	45	45
Defocus range (μm)	−0.65 to −2.15	−0.7 to −4.5	−0.7 to −4.5
Pixel size (Å)	0.4114	1.8850	1.8850
Symmetry imposed	C1	C1	C1
Initial particle images (no.)	349,391	620,012	258,285
Final particle images (no.)	128,330	698,820	193,877
Map resolution (Å)	3.57	4.00	8.00
FSC threshold	0.143	0.143	0.143
Map resolution range (Å)	2.40–23.57	3.50–50.00	8.00–50.00

In the cryo-EM density map there was substantial information loss in side views, due to a preferential end view orientation of the particles (Extended Data Fig. 6b). However, the three seven-turn β-helices and many bulky side chains were well resolved in the end view of the density map (Fig. 4h and Extended Data Fig. 6e), thus allowing docking of the stacked γCAL_181_ trimers from the crystallographic model (Extended Data Fig. 3e). Additional densities were seen to protrude from the edges of the complex above the β10-β11 loop (Fig. 4h), probably representing SSUL modules interacting either intra- or intermolecularly with the γCAL domains. Note that while SSUL2 and SSUL3 may function preferentially to form intermolecular contacts, our mutational analysis showed that all three SSUL modules participate in M58 homodemixing (Fig. 4e). The putative SSUL densities are smaller than the size of the SSUL module and are of low resolution, suggesting a dynamic interaction that precluded docking of bound SSUL. This dynamicity would allow SSUL modules to function in both M58 homodemixing and Rubisco sequestration.

Both γCAL_181_ and γCAL_181_–C2_17_ crystals revealed the presence of two protomer–protomer salt bridges across the dimer interface formed by the conserved residues R164 and D172 (Fig. 4i). Thus, a total of six salt bridges stabilizes the dimer of γCAL trimers. Note that γCAL_198_ and γCAL_181_ are nevertheless trimeric in solution (Supplementary Table 1), suggesting that the head-to-head association occurs only at high protein concentrations within the condensate or crystal. To investigate the functional relevance of this interaction, we disrupted the salt bridges by either mutating R164 to aspartate or D172 to lysine. Strikingly, both R164D and D172K mutants strongly reduced homodemixing of M58 (Fig. 4j), indicating that dimer-of-trimer formation via the γCAL domains provides critical valency, presumably by increasing the local concentration of SSUL modules. Dimerization of hub proteins has been reported to increase valency in other condensate systems as well^50^. In contrast, disruption of the M58 head-to-head dimer did not affect the interaction of CcaA with M58 (Fig. 4k). Here, sufficient avidity is presumably maintained by the intermolecular M58 interactions mediated by SSUL binding to γCAL domains.

In summary, a cooperative network of fluctuating interactions ensures recruitment of CcaA into carboxysomes: (1) the extreme C-terminal sequence (C2) of CcaA binds the γCAL domain of M58, driven by a combination of hydrophobic and electrostatic interactions; (2) the SSUL modules of M58 engage the γCAL domains of adjacent M58 trimers via dynamic multivalent electrostatic interactions, with C2 and SSUL binding to distinct regions on γCAL; and (3) M58 undergoes homodemixing mediated by both intermolecular γCAL-SSUL interactions and a head-to-head association via γCAL trimers.

### M58 binds Rubisco with high affinity

**Fig. 5:**
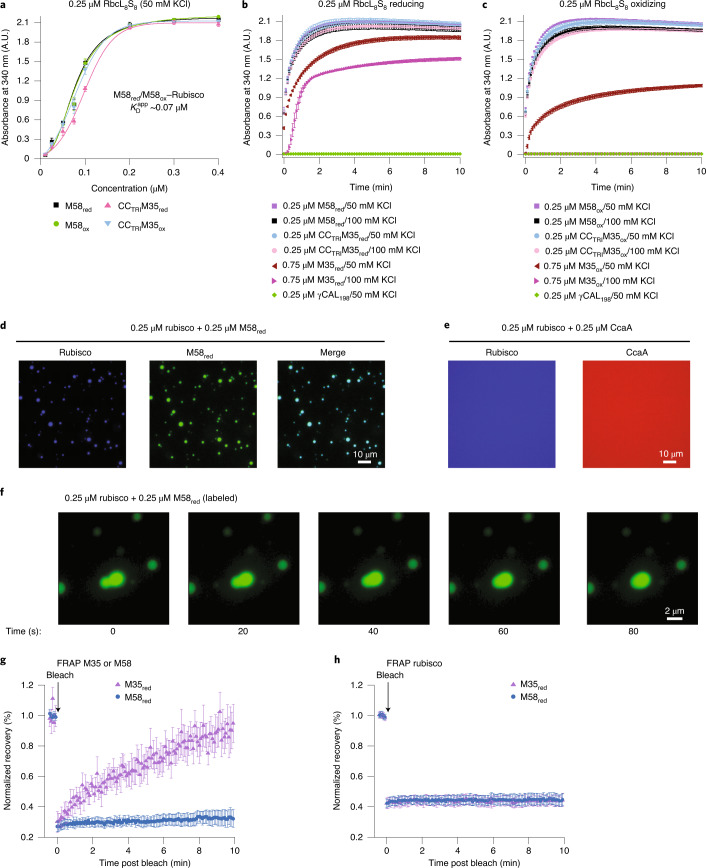
Trimeric M58 binds Rubisco with high affinity. **a**, CC_TRI_M35 mimics the high affinity of M58 for Rubisco, independent of redox state. Condensate formation of 0.25 μM Rubisco (RbcL_8_S_8_) with increasing concentrations of M58_red_, M58_ox_, CC_TRI_M35_red_ and CC_TRI_M35_ox_ was analyzed by turbidity at 50 mM KCl. Data are mean ± s.d. of triplicate measurements. **b**,**c**, M58–Rubisco condensate formation is mediated by the SSUL modules of M58, not by the γCAL domains. Condensate formation of 0.25 μM RbcL_8_S_8_ with M58 (0.25 μM), CC_TRI_M35 (0.25 μM) or M35 (0.75 μM) was analyzed by turbidity assay under reducing (**b**) and oxidizing conditions (**c**) at 50 and 100 mM KCl. Data are mean ± s.d. of triplicate measurements. **d**,**e**, Condensate formation of Rubisco with M58_red_ (**d**) and absence of interaction of Rubisco with CcaA (**e**), as analyzed by fluorescence microscopy. M58, CcaA and Rubisco were N-terminally labeled with Alexa532, Alexa405 and Alexa647, respectively (M58_AF5_; CcaA_AF4_; Rbc_AF6_), and used as 1:10 mixtures with unlabeled protein. Protein concentrations are indicated. Representative data of two independent experiments are shown. **f**, Time-lapse images of droplet fusion. A representative droplet fusion event of the M58_red_–Rubisco condensate (0.25 μM M58_red_/0.25 μM Rubisco) in the presence of 100 mM KCl (from Supplementary Video 3) is shown. M58_red/AF5_ fluorescence was detected. Scale bar, 2 μm. **g**, Mobility of M35 and M58 in condensates with Rubisco. Fluorescence recovery after FRAP experiments is shown for condensates formed by unlabeled Rubisco with either labeled M35_red_ or M58_red_ (0.5 μM Rubisco/2.0 μM M35_red_ or 0.25 μM M58_red_; 100 mM KCl). Prebleach fluorescence is set to 1. Mean ± s.d. from *n* = 20 droplets. **h**, Mobility of Rubisco in condensates with M35 or M58. FRAP experiments are shown for condensates formed by labeled Rubisco with either unlabeled M35_red_ or M58_red_. Concentrations as in **g**. Prebleach fluorescence is set to 1. Mean ± s.d. from *n* = 20 droplets. **a**–**c**,**g**,**h**, Data are available as Source data. Source data

**Extended Data Fig. 7:**
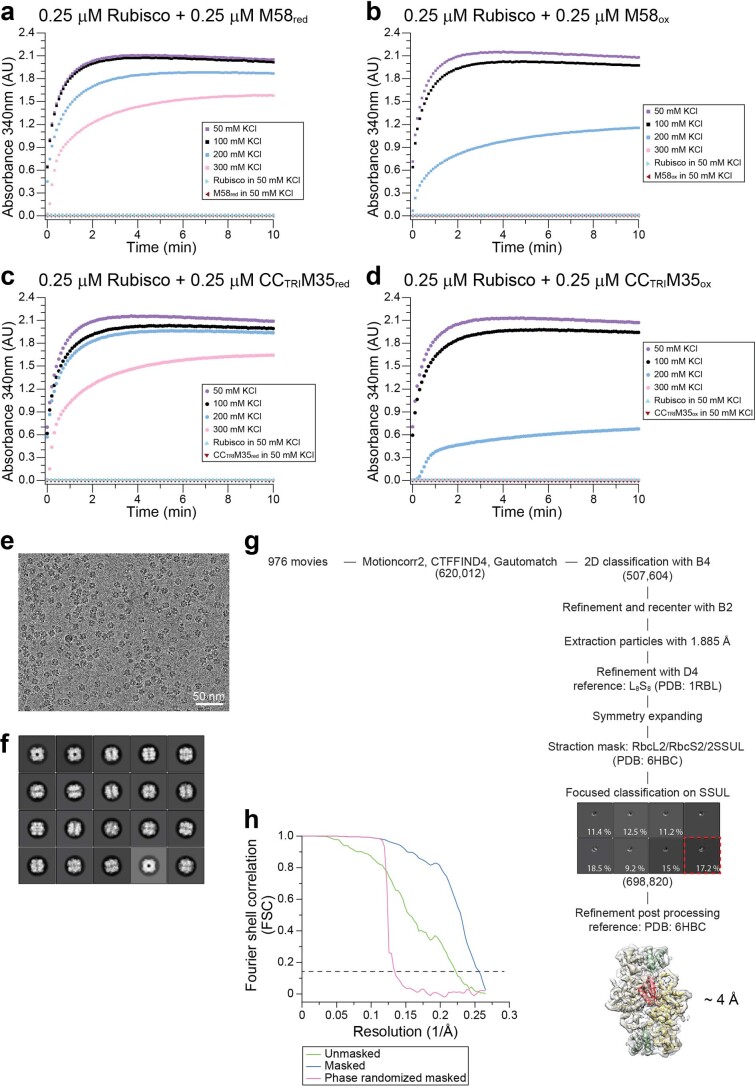
Co-assembly of M58 and Rubisco. **a**, Salt dependence of M58-Rubisco condensate formation under reducing conditions. M58_red_ and Rubisco (0.25 μM each) were incubated in buffer containing 50-300 mM KCl and analyzed by turbidity assay. M58_red_ and Rubisco alone were analyzed as controls at 50 mM KCl. Representative data of two independent experiments are shown. **b**, M58-Rubisco condensate formation is more salt sensitive under oxidizing conditions. M58_ox_ and Rubisco (0.25 μM each) were incubated in buffer containing 50-300 mM KCl and analyzed by turbidity assay as in (a). Representative data of two independent experiments are shown. **c**, Salt dependence of CC_TRI_M35-Rubisco condensate formation under reducing conditions. CC_TRI_M35_red_ and Rubisco (0.25 μM each) were incubated in buffer containing 50-300 mM KCl and analyzed by turbidity assay. CC_TRI_M35_red_ and Rubisco alone were analyzed as controls at 50 mM KCl. Representative data of two independent experiments are shown. **d**, CC_TRI_M35-Rubisco condensate formation is more salt sensitive under oxidizing conditions. CC_TRI_M35_ox_ and Rubisco (0.25 μM each) were incubated in buffer containing 50-300 mM KCl and analyzed by turbidity assay as in (c). Representative data of two independent experiments are shown. **e-h**, Cryo-EM single particle analysis of the M58_red_-Rubisco complex. A representative micrograph (e). Scale bar, 50 nm. Representative micrograph of a total of 976 is shown. 2D class averages of particles (f). The single-particle data processing workflow (g). Particle numbers are in parentheses. B4, 4 × 4 pixel-binned image. B2, 2 × 2 pixel-binned image. D4, D4 symmetry applied. See Methods for details. Gold-standard FSC curves of the RbcL2-RbcS2-SSUL reconstruction from the complex of M58_red_-Rubisco (h) (EMDB: EMD-12731). Data behind the graphs in a-d are available as source data. Source data

The SSUL modules of M35 have recently been shown to link the Rubisco holoenzyme^16^. To investigate how the trimeric M58 interacts with Rubisco, we first determined the apparent affinities of M58_red_ and M58_ox_ for Rubisco. M58 in both redox states displayed essentially identical apparent affinities $(K_{\text{D}}^{\text{app}})$ of ~0.07 μM for Rubisco at 50 mM KCl (Fig. 5a). This interaction was ~three- to tenfold stronger than that of M35 with Rubisco ($K_{\text{D}}^{\text{app}}$ of ~0.2 and ~1 μM under reducing and oxidizing conditions, respectively)^16^, presumably due to the increased local concentration of SSUL modules in the M58 trimer. We confirmed this using the trimeric M35 construct CC_TRI_M35, which also showed high binding affinity for Rubisco (Fig. 5a). The trimeric γCAL_198_ alone, lacking SSUL modules, failed to interact with Rubisco (Fig. 5b,c). Moreover, the interaction of M58 with Rubisco proved to be salt resistant (up to 300 and 200 mM KCl for M58_red_ and M58_ox_, respectively; Extended Data Fig. 7a,b), in contrast to the salt-sensitive interaction of M35 with Rubisco^16^ (Fig. 5b,c). Notably, CC_TRI_M35 mimicked the salt resistance observed with M58 (Extended Data Fig. 7c,d), further demonstrating that this property is due to the high local concentration of SSUL modules in the trimer.

**Extended Data Fig. 8:**
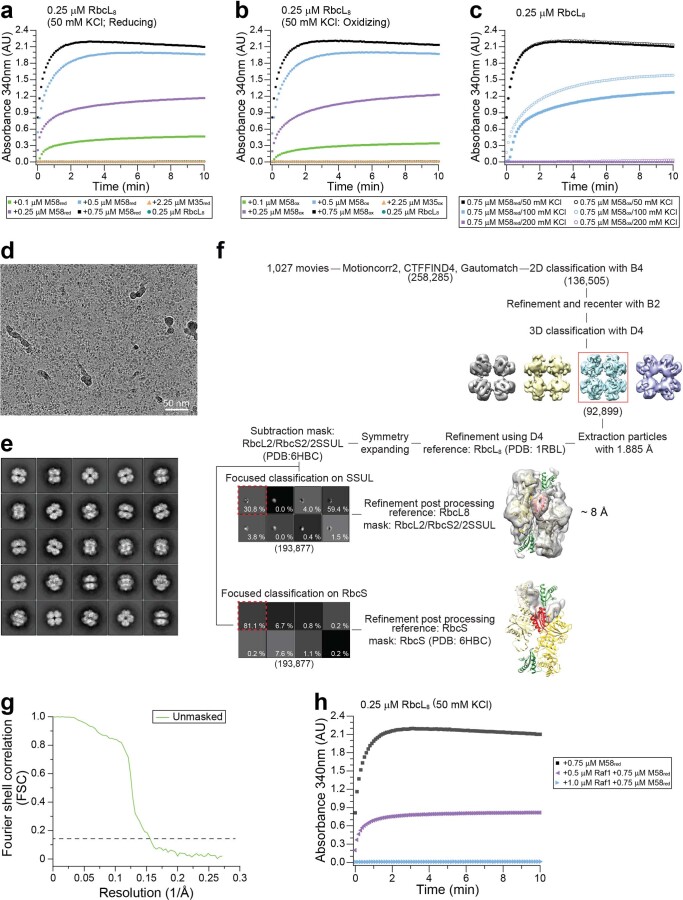
Interaction of SSUL with the RbcL_8_ core complex. **a-c**, The high local concentration of SSUL modules in M58 enables interaction with RbcL_8_. (a) M58-RbcL_8_ condensate formation at different concentrations of M58 under reducing conditions. RbcL_8_ (0.25 μM) was incubated with M58_red_ at the concentrations indicated, and analyzed by turbidity assay. M35_red_-RbcL_8_ and RbcL_8_ were analyzed as controls. Representative data of two independent experiments are shown. (b) M58-RbcL_8_ condensate formation at different concentrations of M58 under oxidizing conditions. RbcL_8_ (0.25 μM) was incubated with M58_ox_ at the concentrations indicated, and analyzed as in (a). M35_ox_-RbcL_8_ and RbcL_8_ were analyzed as controls. Representative data of two independent experiments are shown. (c) Similar salt sensitivity of M58-RbcL_8_ condensate formation under reducing and oxidizing conditions. RbcL_8_ (0.25 μM) was incubated with M58_red_ or M58_ox_ (0.75 μM) in buffer containing 50-200 mM KCl. Reactions analyzed as in (a). Representative data of two independent experiments are shown. **d-g**, Cryo-EM single particle analysis of the M58_red_-RbcL_8_ condensate. (d) A representative micrograph of M58_red_-RbcL_8_ complexes. Scale bar, 50 nm. Representative micrograph of a total of 1,027 is shown. (e) 2D class averages of particles. (f) The single-particle data processing workflow. Particle numbers are indicated in parentheses. B4, 4 × 4 pixel-binned image. B2, 2 × 2 pixel-binned image. D4, D4 symmetry applied. See Methods for details. (g) Gold-standard FSC curve of the unsharpened RbcL2-SSUL reconstruction solved from the complex of M58_red_-RbcL_8_ (EMDB: EMD-12732). **h**, The assembly chaperone Raf1 prevents the interaction of M58 with RbcL_8_. Condensate formation of M58_red_ (0.75 μM) and RbcL_8_ (0.25 μM) was analyzed by turbidity assay at 50 mM KCl in the presence of 0.5 μM and 1.0 μM of Raf1 (concentration of the functional Raf1 dimer). Representative data of two independent experiments are shown. Data behind the graphs in a-c and h are available as source data. Source data

Cryo-EM analysis confirmed that the SSUL modules of M58 bind Rubisco in a groove formed between two RbcL subunits and the adjacent RbcS^16^ (Table 2 and Extended Data Fig. 7e–h). Interestingly, unlike M35, M58 also bound the RbcL_8_ core complex of Rubisco, albeit with ~fourfold lower affinity than the holoenzyme (Extended Data Fig. 8a–c). As revealed by cryo-EM analysis, the SSUL module occupied the same binding site on RbcL_8_ as in the holoenzyme and did not use the RbcS binding region (Table 2 and Extended Data Fig. 8d–g), contrary to a recent suggestion^51^. Thus, the high local SSUL concentration on M58 can compensate for the missing interaction with RbcS^16^. However, the interaction of M58 with RbcL_8_ is unlikely to be important in vivo because Rubisco assembly factors, such as RbcX and/or Raf1 (refs. ^52–54^), bind the RbcL_8_ core and are displaced only after RbcS binding^16^. Indeed, the presence of Raf1 completely suppressed the binding of M58 to RbcL_8_ (Extended Data Fig. 8h), thus ensuring that only the Rubisco holoenzyme is recruited into carboxysomes.

Fluorescence microscopy of Rubisco (N-terminally labeled with Alexa647 and Rubisco_AF6_ and mixed 1:10 with the unlabeled protein) and M58 (M58_AF5_:M58, 1:10) at equimolar concentration resulted in colocalization of both proteins within droplet-shaped condensates (Fig. 5d), while no demixing was observed with Rubisco and CcaA (1:10 of CcaA_AF4_:CcaA) (Fig. 5e). M58–Rubisco droplets underwent fusion at a rate similar to that for M35–Rubisco condensates^16^ (Fig. 5f and Supplementary Videos 3 and 4). Interestingly, FRAP experiments on M58–Rubisco droplets nevertheless showed no recovery of fluorescence signal for either M58 (Fig. 5g) or Rubisco (Fig. 5h). Thus the M58-Rubisco interaction, while sufficiently fluctuating to allow droplet fusion, was rather strong. This contrasts with the M35–Rubisco condensate where M35 was relatively mobile (Fig. 5g) while Rubisco was immobile (Fig. 5h).

In summary, the high local concentration of SSUL modules in trimeric M58 increases the avidity for Rubisco and provides for a higher binding affinity and redox independence compared to monomeric M35. The binding site of SSUL on Rubisco remains the same for M58 and M35.

### Efficient coassembly of M58, M35, CcaA and Rubisco

**Fig. 6:**
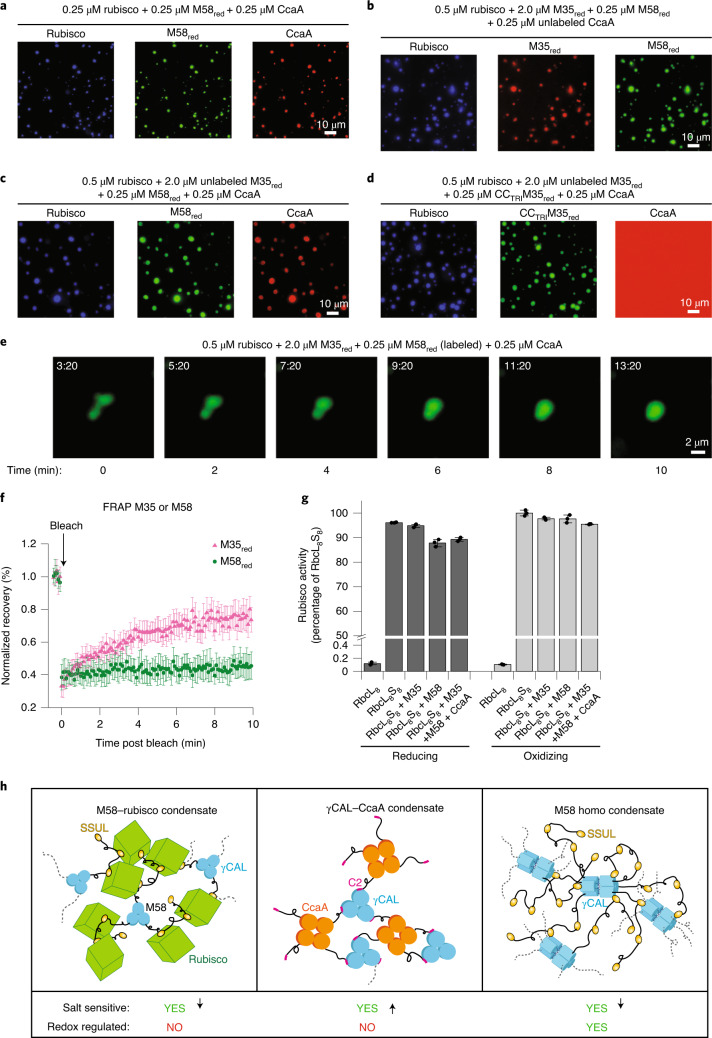
Coassembly of Rubisco, M58, M35 and CcaA. **a**, Coassembly into condensates of Rubisco, M58_red_ and CcaA at the concentrations indicated (100 mM KCl). Proteins were labeled as in Fig. 5d,e. Representative data of two independent experiments are shown. **b**, Coassembly into condensates of fluorescence-labeled Rubisco, M35_red_, and M58_red_ with unlabeled CcaA at the concentrations indicated (100 mM KCl). M35 was N-terminally labeled with Alexa405 (M35_AF4_). Representative data of two independent experiments are shown. **c**, Coassembly of fluorescence-labeled Rubisco, M58_red_ and CcaA with unlabeled M35_red_ at the concentrations indicated (100 mM KCl). Representative data of two independent experiments are shown. **d**, Coassembly of fluorescence-labeled Rubisco, CC_TRI_M35_red_ and CcaA with unlabeled M35_red_. CC_TRI_M35 was N-terminally labeled with Alexa532 (CC_TRI_M35_AF5_). CcaA does not enter the condensate in the absence of M58. Representative data of two independent experiments are shown. **e**, Time-lapse images of droplet fusion. A representative droplet fusion event of four-protein condensates (0.5 μM Rubisco/2 μM M35_red_/0.25 μM M58_red_/0.25 μM), in the presence of 100 mM KCl, from Supplementary Video 5 is shown. M58_red/AF5_ fluorescence is detected. Scale bar, 2 μm. **f**, Mobility of M35 or M58 in condensates of Rubisco, CcaA, M58 and M35. FRAP experiments are shown for condensates formed by the four proteins. Either M35 or M58 was labeled (0.5 μM Rubisco/2 μM M35_red_/0.25 μM M58_red_/0.25 μM CcaA; 100 mM KCl). Prebleach fluorescence is set to 1. Mean ± s.d. from *n* = 20 droplets. **g**, Rubisco (RbcL_8_S_8_) carboxylation activity in condensates with M35, M58 or M35/M58/CcaA. The activity of RbcL_8_S_8_ is set to 100%, with RbcL_8_ as control. Individual data points and error bars represent mean ± s.d. of triplicate measurements. **h**, Versatility of M58-mediated protein interactions in multiprotein phase separation. Left: interaction of trimeric M58 (blue) with Rubisco (green) mediated by SSUL modules of M58 (yellow); middle: interaction of trimeric γCAL domains (blue) of M58 with the C2 sequence of tetrameric CcaA (orange); the C2 peptide is shown in pink; right: M58 homocondensate formation mediated by intermolecular interactions between SSUL modules and γCAL domains and by head-to-head association of γCAL trimers. Dotted lines indicate linker sequences between protein domains interacting with nearby molecules. Effects of increasing salt concentration and redox state of SSUL modules on interactions are indicated. Arrows down and up, reduced or enhanced interaction with increasing salt concentration, respectively. **f**,**g**, Data are available as Source data. Source data

**Extended Data Fig. 9:**
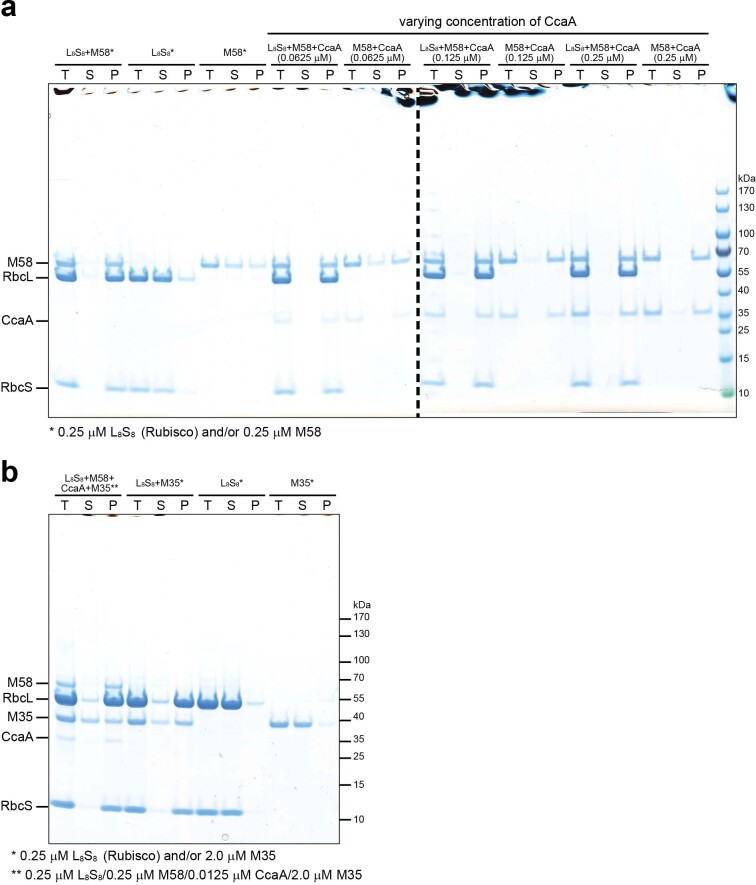
Analysis of condensate formation by sedimentation. **a**, Efficient sequestration of Rubisco (L_8_S_8_) and CcaA by M58_red_. M58_red_-Rubisco and M58_red_-CcaA condensates in the presence of 100 mM KCl were formed as described in Extended Data Fig. 7a and Fig. 1d, respectively, followed by fractionation into total (T), supernatant (S) and pellet (P) by centrifugation (20,817 x g for 20 min at 25 °C), and analysis by SDS-PAGE and Coomassie staining. The concentration of CcaA was varied from 0.0625 to 0.25 μM at a constant concentration of M58_red_ of 0.25 μM. Rubisco (L_8_S_8_) and M58_red_ were analyzed alone as controls. Representative data of two independent experiments are shown. **b**, Condensates of Rubisco (L_8_S_8_), CcaA, M58_red_ and M35_red_ as well as of L_8_S_8_ and M35_red_ were formed at the concentrations indicated and analyzed by sedimentation as in (a). Representative data of two independent experiments are shown.

Carboxysome biogenesis requires the scaffolding proteins M58 and M35 to sequester Rubisco and CcaA in the reducing cytosol. We next analyzed whether the complex interactions described above allow coassembly of these proteins into a distinct condensate. As shown above, M58 undergoes condensate formation with CcaA and Rubisco while M35 forms a condensate only with Rubisco. We first investigated whether Rubisco, M58 and CcaA can coassemble. Assuming that CcaA is substoichiometric to M58 in the carboxysome^26^, we performed a sedimentation assay of the three proteins keeping the concentration of Rubisco and M58 constant (0.25 μM each) and varying the concentration of CcaA. At an equimolar ratio, all three proteins were recovered in the pellet fraction with only a small amount of CcaA remaining in the supernatant (Extended Data Fig. 9a), indicative of highly efficient sequestration. Using this condition, fluorescence microscopy demonstrated the colocalization of all three proteins (differentially labeled) into a phase-separated condensate (Fig. 6a).

M35 is more abundant in carboxysomes than M58 (ref. ^26^). Following the addition of excess M35 (2 μM) to the coassembly reaction of Rubisco/M58/CcaA (0.25, 0.25 and 0.125 μM, respectively), all four proteins were recovered in the pellet fraction following sedimentation, with ~50% of M35 remaining in the supernatant (Extended Data Fig. 9b). Fluorescence microscopy using three fluorophores to label either Rubisco, M35 and M58, or Rubisco, M58 and CcaA showed that all four proteins efficiently colocalized (Fig. 6b,c). When M58 was replaced with CC_TRI_M35, CcaA no longer phase separated and was diffusely distributed (Fig. 6d). The average Feret’s diameter of the condensates varied from ~1.0 to ~2.5 μm depending on total protein concentration (Supplementary Table 2). Droplet fusion of the four-protein condensate occurred at only a very slow rate, indicating low fluidity (Fig. 6e and Supplementary Video 5). M35 mobility by FRAP was somewhat reduced in the condensate of the four proteins compared to the interaction of M35 and Rubisco, while M58 was immobile (Fig. 6f). Notably, the Rubisco enzyme was fully functional in carbon fixation within the condensates (Fig. 6g).

In summary, the scaffolding proteins M58 and M35, differing in binding affinity and dynamics, ensure the efficient sequestration of Rubisco and CcaA for copackaging into carboxysomes. M35 is the only component in the four-protein pre-carboxysome condensate that shows detectable mobility.

## Discussion

β-Carboxysome biogenesis involves the sequestration of Rubisco together with the carbonic anhydrase CcaA, followed by shell formation^55^. Our biochemical and structural analysis elucidated how the scaffolding protein CcmM functions as a central organizer in recruiting Rubisco and CcaA into the pre-carboxysome core. CcmM orchestrates multiple, interwoven coassembly reactions via its γCAL and SSUL domains, resulting in the formation of an essentially immobile protein mesh. Once captured under reducing conditions, constituent carboxysome components cannot escape, facilitating efficient encapsulation. The low dynamics and slow fusion rate of the condensates may be relevant in limiting pre-carboxysome size before shell formation, because aberrantly large carboxysomes are less efficient in the CO_2_-concentrating mechanism^16,56^.

The ~21-kDa β-helical γCAL domain of M58 (CcmM) is remarkably versatile and participates in network formation through multiple cooperative interactions (Fig. 6h). As seen in the crystal structure, each protomer of the trimeric γCAL can bind the 17-residue C2 peptide of one protomer of a CcaA tetramer via specific hydrophobic and charge interactions. However, γCAL-C2 interactions alone are inefficient in mediation of M58–CcaA condensate formation, which requires additional charge interactions of SSUL modules with γCAL at a site distinct from the binding pocket of the C2 peptide of CcaA (Fig. 4g). The multivalency of the network is enhanced further by the ability of γCAL trimers to form head-to-head dimers (Fig. 6h). Similar interactions underlying condensate formation have been reported for other systems to result in relatively low dynamic assemblies^40,50,57^.

**Extended Data Fig. 10:**
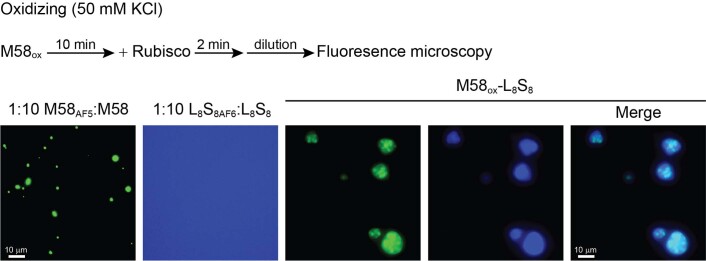
Formation of biphasic M58-Rubisco condensates under oxidizing conditions. M58_ox_-Rubisco condensates were analyzed by fluorescence microscopy using M58_ox_ and Rubisco N-terminally labeled with Alexa532 and Alexa647, respectively (M58_AF5_; L_8_S_8__AF6_), and used as 1:10 mixtures with unlabeled protein. M58_ox_ homo-condensates (3.5 μM) were first allowed to form for 10 min, followed by addition of Rubisco (L_8_S_8_) to reach final concentrations of M58_ox_ and Rubisco of 2.5 μM each. The reaction was diluted 10 times before fluorescence microscopy. Large M58_ox_-Rubisco condensates formed containing dense M58_ox_ foci. The M58_ox_ homo-condensate and Rubisco were also analyzed alone. Images shown are from a single experiment. Note a similar result was observed at 100 mM KCl, however, the overall size of the condensates was smaller.

Recruitment of Rubisco for carboxysome biogenesis is solely mediated by the SSUL modules of M58 and M35, which bind in a groove at the interface between antiparallel RbcL dimers^16^. M58 has a substantially higher affinity for Rubisco than M35, due to the presence of nine SSUL modules per M58 trimer compared to only three in M35. The flexible linkers between SSUL modules apparently do not contribute directly to the interaction, but may play a role in balancing the entropic penalty of SSUL binding. The density, and thus avidity, of SSUL modules would be further increased by the γCAL-mediated head-to-head association of M58 trimers. What then is the role of M35, which is present in excess over M58 (ref. ^26^), and why are both proteins essential for carboxysome biogenesis^31^? We suggest that the differential redox regulation of M35 and M58 is important in converting the immobile pre-carboxysome condensate, required for initial capture of Rubisco and CcaA, into a more dynamic state in the oxidizing interior of the carboxysome. This redox regulation is mediated by disulfide bond formation in SSUL modules, which is critical for carboxysome biogenesis and CCM function in vivo^16^. Oxidation favors the interaction of SSUL modules with γCAL domains, thereby enhancing M58 homodemixing. Indeed, under oxidizing conditions, preformed M58_ox_ condensates were maintained within more dilute and enlarged M58_ox_–Rubisco droplets (Extended Data Fig. 10). The restructuring of the pre-carboxysome condensate following oxidation may promote the formation of channels around the Rubisco lattice that can be navigated by other carboxysomal proteins and metabolites. This would also facilitate the metabolic repair of Rubisco by Rubisco activase, which possesses SSUL modules for recruitment into the pre-carboxysome matrix^19^.

In summary, our findings suggest the following model for pre-carboxysome formation in β-cyanobacteria: in the reducing cytosol, M58 cooperates with M35 to efficiently concentrate Rubisco and CcaA into an immobile matrix for subsequent encapsulation. Redox regulation of SSUL modules in the oxidizing carboxysome then favors homodemixing of M58 and renders the interaction of M35 with Rubisco more dynamic. This transition is required for CCM function.

## Methods

### Strains

*Escherichia coli* DH5α (ThermoFisher) cells were used for the amplification of plasmid DNA. Positive clones were selected and cultivated in lysogeny broth (LB) medium at 37 °C for 8 h. *E. coli* BL21 (DE3) (Agilent) was used for recombinant protein expression (see below).

The cyanobacterium *S. elongatus* PCC 7942 (*Se*7942) (Institut Pasteur Paris) was used to obtain genomic DNA of *ccmM* and *ccaA*. *Se*7942 was cultured in BG-11 medium at 30 °C and 50 r.p.m. under continuous light.

### Plasmids

The oligos used for amplification and generation of plasmids are listed in Supplementary Table 3.

#### Genomic DNA

*Se*7942 was grown to high density and cells pelleted by centrifugation at 10,000*g* for 10 min. The cell pellet was resuspended in 100 ml of buffer (50 mM Tris-HCl pH 8.0/50 mM NaCl) and cells lysed by five cycles of heating (3 min at 95 °C) and snap-freezing in liquid nitrogen. The lysate was centrifuged (20,000*g* for 10 min) and 1 μl of supernatant was used as template in PCR reactions. The full-length *ccmM* gene was amplified using oligo nos. 1/2 and the *ccaA* gene using oligo nos. 67/68 (Supplementary Table 3).

#### Plasmids

The pHUE vector for His6-ubiquitin (H_6_Ub) fusion proteins^58,59^ was used to generate the plasmids used in this study. Plasmids were assembled by PCR and using the Gibson assembly cloning kit (NEB). The plasmids used in this study are listed in Supplementary Table 4 and are available upon request from the corresponding author.

pHUE-*Se*M58 was generated by amplification of the full-length *ccmM* gene from genomic DNA (*Se*7942) and subsequent cloning into the pHUE vector. The shorter constructs containing pHUE-*Se*γCAL-2S(1–429), pHUE-*Se*γCAL-1S(1–313), pHUE-*Se*γCAL(1–198) and pHUE-*Se*γCAL(10–181) were prepared by PCR from pHUE-*Se*M58 and cloned into the pHUE vector. Point mutations in pHUE-*Se*M58 were introduced by QuikChange mutagenesis (Agilent) to generate the following constructs: pHUE-*Se*M58-E17K; pHUE-*Se*M58-D21K; pHUE-*Se*M58-D35K; pHUE-*Se*M58-R37D; pHUE-*Se*M58-R43D; pHUE-*Se*M58-K62D; pHUE-*Se*M58-E76K; pHUE-*Se*M58-R79D; pHUE-*Se*M58-R95D; pHUE-*Se*M58-D112K; pHUE-*Se*M58-R126D; pHUE-*Se*M58-R164D; pHUE-*Se*M58-D172K; pHUE-*Se*M58-E246K; pHUE-*Se*M58-D249K; pHUE-*Se*M58-R251D; pHUE-*Se*M58-R252D; pHUE-*Se*M58-E286K; pHUE-*Se*M58-D294K; pHUE-*Se*M58-R298D; pHUE-*Se*M58-E303K; pHUE-*Se*M58-R367D; and pHUE-*Se*M58-R481D.

pHUE-*Se*M58-C4S (C261S/C279S/C377S/C395S) was generated by replacing the M35 fragment of pHUE-*Se*M58 with M35-C4S from the plasmid pHUE-*Syn*6301*_ccmM_*M35_C261S/ C279S/C377S/C395S^16^, by PCR and subsequent Gibson assembly.

To generate the construct pHUE-CC_TRI_M35, the trimeric coiled-coil sequence GEIAAIKQEIAAIKKEIAAIKQEIAAIKQGS^49^ was inserted into the plasmid pHUE-*Syn*6301_*ccmM*_M35 (ref. ^16^) between the C terminus of ubiquitin and the N terminus of M35, by PCR and subsequent Gibson assembly.

pHUE-*Se*CcaA was generated by amplification of the *ccaA* gene from genomic DNA (*Se*7942) and subsequent cloning into the pHUE vector. pHUE-*Se*CcaAΔC2 and pHUE-C2_17_ were generated by cloning either the first 257 residues (1–257) or the last 17 (256–272) of *Se*CcaA from pHUE-*Se*CcaA into the pHUE vector, by PCR and subsequent Gibson assembly, respectively. Point mutations in pHUE-*Se*CcaA were introduced by QuikChange mutagenesis (Agilent) to generate the constructs pHUE-*Se*CcaA(W257A) and pHUE-*Se*CcaA(R265D).

pHUE-E_GFP_ was generated by amplification of EGFP from pEF-*gfp* (Addgene) with GSGGS at the C terminus and subsequent cloning into pHUE. pHUE-E_GFP_C2_15_ and pHUE-E_GFP_C2_17_ were generated by replacing residues 1–257 or 1–255 of *Se*CcaA, respectively, in pHUE-*Se*CcaA with EGFP-GSGGS by PCR and subsequent Gibson assembly. Point mutations in pHUE-E_GFP_C2_17_ were introduced by QuikChange mutagenesis (Agilent) to generate the constructs pHUE-E_GFP_C2_17_(W257A) and pHUE-E_GFP_C2_17_(R265D).

### Protein expression and purification

The proteins *S*eRubisco^52,60^, *Se*RbcL_8_ (refs. ^52,60^), *Se*M35 (ref. ^16^) and *Se*Raf1 (ref. ^54^) were expressed and purified as previously described. Protein concentrations were determined spectrophotometrically at 280 nm.

#### M58 and mutants

M58 was expressed and purified from *E. coli* BL21 (DE3) cells harboring the pHUE-*Se*M58 plasmid. Briefly, cells were grown in LB medium at 37 °C with shaking at 130 r.p.m. to optical density (OD_600_) 0.4–0.5. Cells were equilibrated to 18 °C (~1 h) and protein expression induced by the addition of 0.2 mM isopropyl β-D-1-thiogalactopyranoside for 14 h/120 r.p.m. Cells were harvested and incubated in buffer A (50 mM Tris-HCl pH 8.0/500 mM NaCl/5% glycerol) containing 20 mM imidazole/1 g l^–1^ lysozyme/2.5 U ml^–1^/*Sm*DNAse/complete protease inhibitor cocktail (Roche) for 1 h before lysis using EmulsiFlex C5 (Avestin, Inc.). After high-speed centrifugation (40,000*g*/40 min/4 °C), the supernatant was loaded on to a gravity Ni-NTA metal affinity column (Qiagen), equilibrated and washed with ten column volumes of buffer A/20 mM imidazole. The bound protein was eluted with buffer A/300 mM imidazole. The H_6_Ub moiety was cleaved using H_6_-Usp2 overnight at 4 °C^59^. The cleaved protein was buffer exchanged on a HiPrep 26/10 desalting column (GE) to buffer A. The protein eluate was then applied to a Ni-NTA column for removal of H_6_-Usp2, the cleaved H_6_Ub moiety and any uncleaved protein. Flowthrough was concentrated to ~3 ml and applied to a size-exclusion column (HiLoad 16/60 Superdex 200, GE) equilibrated in buffer B (50 mM Tris-HCl pH 8.0/500 mM KCl/10% glycerol). Protein-containing fractions were concentrated by ultrafiltration using Vivaspin MWCO 30000 (GE), aliquoted and flash-frozen in liquid N_2_. All M58 point mutant proteins were expressed in *E. coli* BL21 (DE3) cells harboring the respective plasmids and purified as described for wild-type M58.

M58-4C-S, γCAL-2S, γCAL-1S and CC_TRI_M35 were expressed in *E. coli* BL21 (DE3) cells harboring pHUE-*Se*M58-C4S(C261S/C279S/C377S/C395S), pHUE-*Se*γCAL-2S(1-429), pHUE-*Se*γCAL-1S(1-313) or pHUE-CC_TRI_M35, respectively. These proteins were expressed and purified as described for M58.

Reduced M58 proteins were generated by the addition of 5 mM DTT to purified proteins before use. To generate oxidized M58 proteins, purified proteins were incubated before use on ice with 2 mM H_2_O_2_ for 30 min, followed by buffer exchange on a PD MiniTrap G-10 column (GE) to buffer B to remove unreacted H_2_O_2_.

#### γCAL(1–198) and γCAL(1–181)

γCAL(1–198) and γCAL(1–181) were expressed in *E. coli* BL21 (DE3) cells harboring pHUE-*Se*γCAL(1–198) and pHUE-*Se*γCAL(1–181), respectively, essentially as described for M58 except that buffer A contained 150 mM NaCl and buffer B 150 mM KCl. After size-exclusion chromatography (HiLoad 16/60 Superdex 200, GE), purified proteins were concentrated by ultrafiltration using Vivaspin MWCO 10000 (GE), aliquoted and flash-frozen in liquid N_2_.

#### CcaA and CcaAΔC2

CcaA, CcaAΔC2, CcaA(W257A) and CcaA(R265D) were expressed in *E. coli* BL21 (DE3) cells from the plasmids pHUE-*Se*CcaA, pHUE-*Se*CcaAΔC2, pHUE-*Se*CcaA(W257A) or pHUE-*Se*CcaA(R265D), respectively, and purified as described for γCAL. Proteins were concentrated by ultrafiltration using Vivaspin MWCO 30000 (GE), aliquoted and flash-frozen in liquid N_2_.

#### E_GFP_, E_GFP_C2_15_ and E_GFP_C2_17_

*E. coli* BL21 (DE3) cells harboring pHUE-E_GFP_, pHUE-E_GFP_C2_15_, pHUE-E_GFP_C2_17_, pHUE- E_GFP_C2_17_(W257A) or pHUE- E_GFP_C2_17_(R265D) were used to express E_GFP_, E_GFP_C2_15_, E_GFP_C2_17_, E_GFP_C2_17_(W257A) and E_GFP_C2_17_(R265D), respectively, and purified as described for γCAL. After size-exclusion chromatography (HiLoad 16/60 Superdex 75, GE), the purified proteins were concentrated by ultrafiltration using Vivaspin MWCO 10000 (GE), aliquoted and flash-frozen in liquid N_2_.

#### γCAL and C2_17_ peptide for crystallography

For crystallization trials, purified γCAL_198_ and γCAL_181_ were buffer exchanged to buffer C (20 mM Tris-HCl pH 8.0/150 mM NaCl) using a PD MiniTrap G-10 column (GE). The C2_17_ peptide (residues 256–272) of *Se*CcaA was produced by expressing the vector pHUE-C2_17_ containing H_6_Ub-tagged C2_17_, with purification by Ni-NTA column as described for γCAL. The eluate from the Ni-NTA column was concentrated and applied onto a size-exclusion column (HiLoad 16/60 Superdex 75, GE) equilibrated in buffer C. Protein-containing fractions were collected and cleaved by H_6_-Usp2 overnight at 4 °C. The cleaved protein was applied to a Ni-NTA column for removal of H_6_-Usp2, the cleaved H_6_Ub moiety and any uncleaved protein. C2_17_ peptide in the flowthrough was mixed with γCAL(1–198) or γCAL(1–181) at a molar ratio of 6:1, and incubated for 1 h at 4 °C to generate the complex. The respective complexes were purified by size-exclusion chromatography (HiLoad 16/60 Superdex 75 equilibrated in buffer C). Protein-containing fractions were concentrated by ultrafiltration using Vivaspin MWCO 3000 (GE), aliquoted and flash-frozen in liquid N_2_. The presence of the C2_17_ peptide in the complex was confirmed by MS.

### Turbidity assay

Measurements were performed at 25 °C in buffer (50 mM Tris-HCl pH 8.0, 10 mM Mg(OAc)_2_) containing different concentrations of KCl and in the presence or absence of 5 mM DTT, as indicated in figure legends. Reactions (100 μl) containing proteins as stated in figure legends were rapidly mixed by vortexing, and absorbance at 340 nm was monitored over time on a Jasco V-560 spectrophotometer set to 25 °C. Generally, proteins from two independent purification batches were analyzed repeatedly. Data were plotted using Origin 2020.

### Rubisco activity assay

Rubisco activity assay were performed essentially as described previously^54,61^. Reactions (50 μl) were performed at 25 °C in buffer (50 mM Tris-HCl pH 8.0, 100 mM KCl) containing Rubisco (RbcL_8_S_8_, 0.5 μM) and M35 (2 μM) or M58 (0.25 μM), or Rubisco and M35 (2 μM)/M58 (0.25 μM)/CcaA (0.25 μM), and were incubated for 10 min in the presence or absence of 5 mM DTT to allow condensate formation. Rubisco-active sites were activated by the addition of 20 μl of premix (50 mM Tris-HCl pH 8.0, 100 mM KCl, 150 mM MgCl_2_, 250 mM NaHCO_3_, 4.5 mM NaH^14^CO_3_ (specific activity 56.6 mCi mmol^–1^)) and reactions incubated for a further 10 min. The carboxylation reaction was initiated by the addition of 30 μl of ribulose-1,5-bisphosphate (10 mM) and stopped by the addition of 20 μl of formic acid (18 M) after 5 min. The amount of carbon fixed was quantified using a HITACHI AccuFLEX LSC-8000 scintillation counter. The activity of RbcL_8_S_8_ is set to 100% and that of RbcL_8_ was measured as control. Proteins from the same purification batches were analyzed repeatedly. Data were plotted using Origin 2020.

### Condensate formation analysis by fluorescence microscopy

Proteins to be analyzed for phase separation by microscopy were fluorescently labeled at the N terminus. Rubisco holoenzyme was labeled with Alexa Fluor 647 NHS ester (RbcLS_AF6_, ThermoFisher) according to the manufacturer’s instructions (~4.6 dye molecules bound per Rubisco holoenzyme). M58_ox_, CC_TRI_M35_ox_ and γCAL(1–198) were labeled with the fluorophore Alexa Fluor 532 NHS ester (ThermoFisher) (M58_AF5_; CC_TRI_M35_AF5_; γCAL_AF5_: ~2.0, ~2.1 and ~1.4 dye molecules bound per M58_ox_ trimer, CC_TRI_M35_ox_ trimer and γCAL(1–198) trimer, respectively), while M35_ox_, CcaA and CcaAΔC2 were labeled with the fluorophore Alexa Fluor 405 NHS ester (ThermoFisher) (M35_AF4_; CcaA_AF4_; CcaAΔC2_AF4_: ~0.8, ~1.2 and ~3.0 dye molecules bound per M35_ox_, CcaA tetramer and CcaAΔC2 tetramer, respectively). Labeled protein was mixed with unlabeled protein at a ratio of 1:10. Reactions (20 μl in 50 mM Tris pH 8.0/10 mM Mg(OAc)_2_) with 50 or 100 mM KCl performed in the presence or absence of 5 mM DTT, and proteins at the concentrations stated in figure legends, were combined. After incubation for 5 min at 25 °C, reactions were transferred to an uncoated chambered coverslip (μ-Slide angiogenesis, Ibidi) followed by incubation for a further 5 min before analysis. For the analysis of droplet fusion, reactions (20 μl) contained 10% labeled M58_red/AF5_ or M35_red/AF5_, with unlabeled Rubisco and other proteins as indicated in the figure legends. After preparing each reaction in a low-binding microcentrifuge tube with protein concentrations as stated in the figure legends, the reaction was transferred to an uncoated chambered coverslip (μ-Slide angiogenesis; Ibidi) without further incubation and videos were recorded in a single focal plane at 5-s time intervals for 20 min. Samples were illuminated with a Lumencor SPECTRA X Light Engine at 398, 558 and 640 nm for fluorescence imaging. Images were recorded by focusing on the bottom of the plate using a Leica Thunder Widefield 2 microscope with a Leica DFC9000 GTC camera and a HC PL APO ×63/1.47 numerical aperture oil objective, using Leica Application Suite X software. Generally, proteins from two independent purification batches were analyzed repeatedly.

The software Fiji^62^ was used for analysis of size distribution of droplets. In brief, after preprocessing of images with background subtraction and Gaussian blur, the MaxEntropy method was applied to determine the threshold of segmentation.

### Fluorescence recovery following photobleaching

FRAP experiments were carried out with a Leica TCS SP8 AOBS confocal laser scanning microscope (HCX PL APO 63×/1.2 water objective, PMT detector). Rubisco holoenzyme was labeled with Alexa Fluor 532 NHS ester (Rubisco_AF5_, ThermoFisher) (~3.6 dye molecules bound per Rubisco holoenzyme), and other proteins were labeled as described above. Reactions (20 μl) in buffer D (50 mM Tris pH 8.0/100 mM KCl/10 mM Mg(OAc)_2_) in the presence or absence of 5 mM DTT, and proteins at the concentrations stated in figure legends, were combined. After incubation for 5 min at 25 °C, reactions were transferred to an uncoated chambered coverslip (μ-Slide angiogenesis, Ibidi) followed by incubation for a further 15 min before analysis. Images before and 10 min after photobleaching were recorded in a single focal plane at 5-s time intervals. Bleaching was performed with a bleach point model using either a 405-nm diode laser at 2% intensity or a 532-nm argon laser at 100% intensity in one repeat, with a dwell time of 100 ms. The software Fiji was used for image analysis^62^. Proteins from the same purification batches were analyzed repeatedly.

### Size-exclusion chromatography coupled to SEC–MALS

Purified proteins (2 mg ml^–1^) were analyzed using static and dynamic light scattering by autoinjection of the sample onto a SEC column (5 μm, 4.6 × 300 mm^2^ column; Wyatt Technology, no. WTC-030N5) at a flow rate of 0.20 or 0.25 ml min^–1^ in buffer (50 mM Tris-HCl pH 8.0/150 mM KCl or 50 mM Tris-HCl pH 8.0/500 mM KCl/5 mM DTT) at 25 °C. The column was in line with the following detectors: a variable ultraviolet absorbance detector set at 280 nm (Agilent 1100 series), a DAWN EOS MALS detector (Wyatt Technology, 690-nm laser) and an Optilab rEX refractive index detector (Wyatt Technology, 690-nm laser)^63^. Molecular masses were calculated using ASTRA 5 software (Wyatt Technology) with the dn/dc value set to 0.185 ml g^–1^. Bovine serum albumin (ThermoFisher) was used as the calibration standard. The graphs shown in Extended Data Fig. 1b,c were generated using SigmaPlot 14.

### ITC

ITC measurements were carried out on a ITC200 calorimeter (Microcal, GE) at 20 °C. After dialysis into buffer D, E_GFP_C2_17_ (365 μM) was loaded into the syringe and titrated into the sample cell containing γCAL(1–198) (14 μM). The reference cell contained buffer D. For each titration point, 10 μl of E_GFP_C2_17_ was injected at time intervals of 3 min. Titration data were analyzed using the software Origin 2020 and fitted with a one-site binding model. Proteins from the same purification batches were analyzed twice.

### Crystallization and data collection

Crystals of γCAL(1–181) and γCAL(1–181)-C2_17_ were grown by the sitting-drop vapor diffusion method at 20 °C. Drops containing 0.6 μl of a 1:1 mixture of 10 mg ml^–1^ protein in buffer C and precipitant (0.1 M HEPES pH 7.5/25% PEG-3350) were equilibrated against 100 μl of precipitant. For cryomounting, crystals were transferred into cryo-buffer (0.1 M HEPES pH 7.5/25% PEG-3350/10% glycerol) and subsequently cryocooled by dipping into liquid N_2_.

#### Crystallographic data collection, structure solution and refinement

The diffraction data for γCAL(1–181) and γCAL(1–181)-C2_17_ crystals were collected at beamline ID23-2 using MXCuBE3 and a wavelength of 0.87313 Å at the European Synchrotron Radiation Facility (ESRF) in Grenoble, France, and beamline X06SA using the SSX suite and a wavelength of 0.99989 Å at the Swiss Synchrotron Light Source (SLS) in Villigen, Switzerland, respectively, with crystals maintained at 100 K and processed with autoPROC (Global Phasing)^64^ using XDS^65^, POINTLESS^66^ and AIMLESS^67^.

The structure of γCAL(1–181) was solved to 1.67-Å resolution by molecular replacement using the program MOLREP^68^ with the γCAL(1–209) domain of *T. elongatus* BP-1 (PDB: 3KWC) as a search template. The asymmetric unit contained one γCAL(1–181) protomer with residues 1–15 disordered. The model was edited manually using Coot^69^ as implemented in the CCP4i graphical user interface^70^. REFMAC5 was used for model refinement^71^. The model of γCAL(1–181) contains 127 ordered water molecules, a presumably ordered Cl^−^ ion and a Ni^2+^ atom from the Ni-NTA metal affinity column. The bound Ni^2+^ atom was identified by X-ray fluorescence scanning.

The structure of γCAL(1–181)-C2_17_ was solved to 1.63-Å resolution by molecular replacement using the γCAL(1–181) model and refined as described above. The asymmetric unit contained one γCAL(1–181) protomer with residues 1–15 disordered, one bound C2_17_ peptide with residues 256–270 resolved (Extended Data Fig. 3g), one presumably ordered Cl^−^ ion, one Ni^2+^ ion and 103 ordered water molecules.

### Structure analysis

The quality of the structural models was analyzed with the program Molprobity^72^. The final models of γCAL(1–181) and γCAL(1–181)-C2_17_ exhibited reasonable stereochemistry with 98.2 and 98.3% of residues, respectively, in the favored regions of the Ramachandran plot and no residues in outlier regions. Coordinates were aligned with Lsqkab and Lsqman^73^. Molecular interfaces were analyzed with PISA^48^ and figures were created with PyMol (http://www.pymol.org/).

### Cryoelectron microscopy and reconstruction

#### Sample preparation and data collection

All cryogrids were prepared with a Vitrobot Mark 4 (FEI). A sample volume of 3 μl was applied to a glow-discharged grid (Quantifoil R2/1 300-mesh) at 25 °C and 90% humidity, then semiautomatically blotted and plunge-frozen into liquid ethane.

For the analysis of M58 head-to-head complexes, 6 μM M58_ox_ was incubated in buffer (50 mM Tris-HCl pH 8.0, 10 mM Mg(OAc)_2_, 50 mM KCl) at 25 °C for 10 min and cryogrids prepared as described above. Eight cryogrids were initially screened on a Glacios transmission electron microscope (ThermoFisher) equipped with a K2 summit direct electron detector (Gatan), operated at 200 keV. Selected grids were transferred to a Titan Krios 300-kV TEM (FEI) equipped with GIF Quantum Energy Filters (Gatan) and a K3 direct detector (Gatan). A total of 1,836 videos were automatically collected by SerialEM^74^ using a pixel size of 0.4114 Å. The total exposure time of 1.2 s was divided into 20 frames with an accumulated dose of 60 electrons Å^–2^ and a defocus range of –0.65 to –2.15 μm.

The complexes M58_red_–Rubisco and M58_red_–RbcL_8_ were prepared by mixing Rubisco (6 μM) and M58_red_ (8 μM), or RbcL_8_ (6.25 μM) and M58_red_ (16.7 μM) in buffer (50 mM Tris-HCl pH 8.0, 10 mM Mg(OAc)_2_, 50 mM KCl, 5 mM DTT), respectively, for 10 min at 25 °C and cryogrids prepared as described above. The cryogrids were screened on a Glacios transmission electron microscope (ThermoFisher) equipped with a K2 summit direct electron detector (Gatan), operated at 200 keV. The selected grid on stage was used for data collection directly with K2 summit. Exposure times of 12 s were divided into 40 frames with an accumulated dose of 45 electrons Å^–2^. In total, 976 and 1,027 videos were automatically collected for M58_red_–Rubisco and M58_red_–RbcL_8_, respectively, by SerialEM^74^ with a pixel size of 1.885 Å and a defocus range of –0.7 to –4.5 μm.

#### Image processing

##### M58

On-the-fly processing during data collection was performed with MotionCor2 (ref. ^75^) and CTFFIND-4.1 (ref. ^76^), as implemented in Focus software^77^. Only micrographs with good signal quality and with an estimated maximum resolution <5 Å were kept for further data processing with RELION 3.1 (ref. ^78^) (Extended Data Fig. 6). A total of 349,391 particles were autopicked by Gautomatch (https://www2.mrc-lmb.cam.ac.uk/research/locally-developed-software/zhang-software/) and extracted at a pixel size of 1.65 Å (fourfold binned). The first round of 2D classification was performed to exclude ice contaminants and classes with no structural features. Because the side-view classes suggested a head-to-head stack of two γCAL domains, the crystallographic model of the γCAL dimer-of-trimers (PDB: 7O4Z) was converted to an EM density map in mrc format with a low-pass filter to 15 Å, which was used as a reference for 3D classification and refinement. The selected particles were subjected to one round of refinement, and new particles were extracted with refined coordinates (recenter) at a pixel size of 0.82 Å. Three-dimensional classification resulted in one major class containing 128,330 particles (Extended Data Fig. 6c), which were subjected to contrast transfer function (CTF) refinement and polishing to generate the final map at 3.57-Å resolution, determined by gold-standard Fourier shell correlation (FSC) with a cutoff at 0.143 (Extended Data Fig. 6d). The particle distribution plot suggested a lack of information in side view (Extended Data Fig. 6b) and, as a result, the side view of the reconstruction was stretched. Based on the information from the well-resolved end view (Extended Data Fig. 6e), we docked the crystallographic model of γCAL trimers into the EM density map using Chimera^79^. The EM density map is deposited with EMDB under the accession code EMD-12730.

##### M58_red_–Rubisco

The raw videos of the dataset for the M58_red_–Rubisco complex were first processed with MotionCor2 (ref. ^75^) with dose-weighting. CTFFIND-4.1 (ref. ^76^) estimated the CTF parameters for each micrograph. A total of 620,012 particles were picked by Gautomatch (https://www2.mrc-lmb.cam.ac.uk/research/locally-developed-software/zhang-software/) (Extended Data Fig. 7e–h). Particles were first extracted at a pixel size of 7.54 Å (fourfold binned). One round of 2D classification resulted in 507,604 clean particles, with ice contaminants and classes with no structural features excluded (Extended Data Fig. 7f,g). These particles were refined with a low-resolution reference converted from the crystal structure coordinates of the Rubisco holoenzyme (PDB: 1RBL), and extracted at a pixel size of 3.77 Å. A single round of 3D classification with *D4* symmetry resulted in four classes with no major differences. Thus, particles from all four classes were subjected to further analysis. These particles were again extracted with full resolution at a pixel size of 1.885 Å. We next followed the same symmetry-expansion procedure previously published^16^—that is, particles were first aligned with *D4* symmetry to account for multiple SSUL modules bound per Rubisco. Each asymmetric unit was processed as an individual particle, which is achieved by the symmetry-expanding command, relion_particle_symmetry_expand, and particle subtraction was done based on a mask covering two RbcL, two RbcS and two SSUL. A focused classification with a SSUL mask resulted in one class of particles with detailed SSUL features. A total of 698,820 particles from this class were selected and subjected to final local refinement, and the postprocessing job pushed the resolution of the EM density map to ~4 Å as determined by gold-standard FSC curve at 0.143 cutoff (Extended Data Fig. 7g,h). Two EM density maps were deposited with EMDB under the accession code EMD-12731, one without sharpening applied and the other sharpened with DeepEMhancer (10.1101/2020.06.12.148296).

##### M58_red_–RbcL_8_

The raw videos of the dataset for the M58_red_–RbcL_8_ complex were first processed with MotionCor2 (ref. ^75^) with dose-weighting. CTFFIND-4.1 (ref. ^76^) and the CTF parameters for each micrograph were estimated. In total, 258,285 particles were picked by Gautomatch (http://www.mrc-lmb.cam.ac.uk/kzhang/Gautomatch). Particles were first extracted at a pixel size of 7.54 Å (fourfold binned). One round of 2D classification excluded ice contaminations and classes with no structural features, resulting in 136,505 clean particles (Extended Data Fig. 8d–f). These particles were refined with a low-resolution reference converted from the RbcL_8_ crystal structure coordinates (PDB: 1RBL with RbcS subunits deleted), and extracted at a pixel size of 3.77 Å. A single round of 3D classification identified a major class with detailed RbcL_8_ features (92,899 particles) (Extended Data Fig. 8f). These particles were again extracted with full resolution at a pixel size of 1.885 Å. We next followed the same symmetry-expansion used for image processing of M58_red_–Rubisco (see above). Focused classification with a SSUL mask resulted in a single class (193,877 particles) with detailed SSUL features (Extended Data Fig. 8f). This class was subjected to final local refinement yielding a map at ~8-Å resolution without postprocessing (Extended Data Fig. 8f). To exclude the bias due to focused classification on SSUL, we performed another round of focused classification with a mask covering one RbcS subunit. Classification resulted in one dominant class containing no EM density in the region where RbcS is bound. The previously published model of RbcL2-RbcS2-SSUL (PDB: 6HBC)^16^ was fitted into the experimental EM density map using Chimera^79^. Two maps were deposited with EMDB under accession code EMD-12732, one without sharpening applied and the other sharpened with DeepEMhancer (10.1101/2020.06.12.148296). The resolution was determined by a gold-standard FSC curve at 0.143 cutoff (Extended Data Fig. 8g).

### Sequence alignment

Conservation of protein sequences was analyzed using the ConSurf web server^80^. Searching of sequences homologous to *Se*SSUL (219–311) or *Se*CcmM (1–539) was performed against the UniProt database. HMMER^81^, three and 0.0001 were set for homolog search algorithm, number of iterations and E-value cutoff, respectively. Multiple sequence alignment containing 500 SSUL or 150 γCAL homologous sequences was built using MAFFT^82^ and submitted to the WebLogo server^83^ to create the sequence logos.

### Statistics

All relevant biochemical experiments were replicated two or three times. No statistical methods were used to predetermine sample size, but our sample sizes are similar to those reported in previous publications^16,19^. For cryo-EM, data were screened on eight independently prepared samples.

### Reporting Summary

Further information on research design is available in the Nature Research Reporting Summary linked to this article.

## Online content

Any methods, additional references, Nature Research reporting summaries, source data, extended data, supplementary information, acknowledgements, peer review information; details of author contributions and competing interests; and statements of data and code availability are available at 10.1038/s41594-021-00676-5.

## Supplementary information

Supplementary Information Supplementary Tables 1, 2 and 4 and legend for Table 3 (Excel), named Zang_Wang et al_Supplementary Info_NSMB-A44989-R2.xlsx (see below).

Reporting Summary

Peer Review Information

Supplementary Table 1 Oligo sequences used in this study.

Supplementary Video 1 Time-lapse video of condensates of M58_red_ and CcaA. Condensates of M58_red_ (0.25 μM) and CcaA (0.25 μM) in the presence of 100 mM KCl. M58_red__/AF5_ fluorescence was detected. Scale bar, 5 μm.

Supplementary Video 2 Time-lapse video of condensates of M58_ox_. Condensate of M58_ox_ (2.5 μM) in the presence of 50 mM KCl. M58_ox__/AF5_ fluorescence was detected. Scale bar, 5 μm.

Supplementary Video 3 Time-lapse video of condensates of M58_red_ and Rubisco. Condensates of M58_red_ (0.25 μM) and Rubisco (0.25 μM) in the presence of 100 mM KCl. M58_red__/AF5_ fluorescence was detected. Scale bar, 5 μm.

Supplementary Video 4 Time-lapse video of condensates of M35_red_ and Rubisco. Condensates of M35_red_ (2.0 μM) and Rubisco (0.25 μM) in the presence of 50 mM KCl. M35_red__/AF5_ fluorescence was detected. Scale bar, 5 μm.

Supplementary Video 5 Time-lapse video of four-protein condensate. Four-protein condensates (0.5 μM Rubisco/2 μM M35_red_/0.25 μM M58_red_/0.25 μM CcaA) in the presence of 100 mM KCl. M58_red__/AF5_ fluorescence was detected. Scale bar, 5 μm.

## Data Availability

The crystallographic structure factors and models for complexes *Se*γCAL_181_ and *Se*γCAL_181_–C2_17_ have been deposited with the PDB database under accession code nos. 7O4Z and 7O54, respectively. Local electron density maps for *Se*M58_ox_, *Se*M58_red_-*Se*Rubisco and *Se*M58_red_-*Se*RbcL_8_ have been deposited under EMDB accession code nos. EMD-12730, EMD-12731 and EMD-12732, respectively. Source data are provided with this paper. Other data are available from the corresponding author upon reasonable request.

## Source data

Source Data Fig. 1 Raw values for graphs shown in Fig. 1d–f.
Source Data Fig. 2 Raw values for graphs shown in Fig. 2b,d,e.
Source Data Fig. 3 Raw values for graph shown in Fig. 3d.
Source Data Fig. 4 Raw values for graphs shown in Fig. 4a,b,d–f,j,k.
Source Data Fig. 5 Raw values for graphs shown in Fig. 5a–c,g,h.
Source Data Fig. 6 Raw values for graphs shown in Fig. 6f,g.
Source Data Extended Data Fig. 1 Raw values for graphs shown in Extended Data Fig. 1b,c.
Source Data Extended Data Fig. 2 Raw values for graphs shown in Extended Data Fig. 2a,b,e.
Source Data Extended Data Fig. 4 Raw values for graphs shown in Extended Data Fig. 4a,c,e,f,h.
Source Data Extended Data Fig. 7 Raw values for graphs shown in Extended Data Fig. 7a–d.
Source Data Extended Data Fig. 8 Raw values for graphs shown in Extended Data Fig. 8a–c,h.

## References

[1] Kerfeld CA, Aussignargues C, Zarzycki J, Cai F, Sutter M (2018). Bacterial microcompartments. Nat. Rev. Microbiol..

[2] Espie GS, Kimber MS (2011). Carboxysomes: cyanobacterial Rubisco comes in small packages. Photosynth. Res..

[3] Turmo A, Gonzalez-Esquer CR, Kerfeld CA (2017). Carboxysomes: metabolic modules for CO_2_ fixation. FEMS Microbiol. Lett..

[4] Greening C, Lithgow T (2020). Formation and function of bacterial organelles. Nat. Rev. Microbiol..

[5] Gonzalez-Esquer CR, Shubitowski TB, Kerfeld CA (2015). Streamlined construction of the cyanobacterial CO_2_-fixing organelle via protein domain fusions for use in plant synthetic biology. Plant Cell.

[6] Hanson MR, Lin MT, Carmo-Silva AE, Parry MA (2016). Towards engineering carboxysomes into C3 plants. Plant J..

[7] Long BM, Rae BD, Rolland V, Forster B, Price GD (2016). Cyanobacterial CO_2_-concentrating mechanism components: function and prospects for plant metabolic engineering. Curr. Opin. Plant Biol..

[8] Rae BD (2017). Progress and challenges of engineering a biophysical CO_2_-concentrating mechanism into higher plants. J. Exp. Bot..

[9] Giessen TW, Silver PA (2017). Engineering carbon fixation with artificial protein organelles. Curr. Opin. Biotechnol..

[10] Kubis A, Bar-Even A (2019). Synthetic biology approaches for improving photosynthesis. J. Exp. Bot..

[11] Kirst H, Kerfeld CA (2019). Bacterial microcompartments: catalysis-enhancing metabolic modules for next generation metabolic and biomedical engineering. BMC Biol..

[12] Wunder T, Mueller-Cajar O (2020). Biomolecular condensates in photosynthesis and metabolism. Curr. Opin. Plant Biol..

[13] Hennacy JH, Jonikas MC (2020). Prospects for engineering biophysical CO(_2_) concentrating mechanisms into land plants to enhance yields. Annu. Rev. Plant Biol..

[14] Zhu XG, Ort DR, Parry MAJ, von Caemmerer S (2020). A wish list for synthetic biology in photosynthesis research. J. Exp. Bot..

[15] Borden JS, Savage DF (2021). New discoveries expand possibilities for carboxysome engineering. Curr. Opin. Microbiol..

[16] Wang H (2019). Rubisco condensate formation by CcmM in beta-carboxysome biogenesis. Nature.

[17] Oltrogge LM (2020). Multivalent interactions between CsoS2 and Rubisco mediate α-carboxysome formation. Nat. Struct. Mol. Biol..

[18] Lechno-Yossef S (2020). Cyanobacterial carboxysomes contain an unique Rubisco-activase-like protein. New Phytol..

[19] Flecken M (2020). Dual functions of a Rubisco activase in metabolic repair and recruitment to carboxysomes. Cell.

[20] Kaplan A (2017). On the cradle of CCM research: discovery, development, and challenges ahead. J. Exp. Bot..

[21] Rae BD, Long BM, Badger MR, Price GD (2013). Functions, compositions, and evolution of the two types of carboxysomes: polyhedral microcompartments that facilitate CO_2_ fixation in cyanobacteria and some proteobacteria. Microbiol. Mol. Biol. Rev..

[22] Kerfeld CA, Melnicki MR (2016). Assembly, function and evolution of cyanobacterial carboxysomes. Curr. Opin. Plant Biol..

[23] Kimber, M. S. in *Carbonic Anhydrase: Mechanism, Regulation, Links to Disease, and Industrial Applications* (eds Frost, S. C. & McKenna, R.) 89–103 (Springer, 2014).

[24] Dou Z (2008). CO_2_ fixation kinetics of *Halothiobacillus neapolitanus* mutant carboxysomes lacking carbonic anhydrase suggest the shell acts as a diffusional barrier for CO_2_. J. Biol. Chem..

[25] Hayer-Hartl M, Hartl FU (2020). Chaperone machineries of Rubisco – the most abundant enzyme. Trends Biochem. Sci..

[26] Long BM, Badger MR, Whitney SM, Price GD (2007). Analysis of carboxysomes from *Synechococcus* PCC7942 reveals multiple Rubisco complexes with carboxysomal proteins CcmM and CcaA. J. Biol. Chem..

[27] Cot SS, So AK, Espie GS (2008). A multiprotein bicarbonate dehydration complex essential to carboxysome function in cyanobacteria. J. Bacteriol..

[28] Kinney JN, Salmeen A, Cai F, Kerfeld CA (2012). Elucidating essential role of conserved carboxysomal protein CcmN reveals common feature of bacterial microcompartment assembly. J. Biol. Chem..

[29] Peña KL, Castel SE, de Araujo C, Espie GS, Kimber MS (2010). Structural basis of the oxidative activation of the carboxysomal gamma-carbonic anhydrase, CcmM. Proc. Natl Acad. Sci. USA.

[30] So AK, Espie GS (1998). Cloning, characterization and expression of carbonic anhydrase from the cyanobacterium *Synechocystis* PCC6803. Plant Mol. Biol..

[31] Long BM, Tucker L, Badger MR, Price GD (2010). Functional cyanobacterial beta-carboxysomes have an absolute requirement for both long and short forms of the CcmM protein. Plant Physiol..

[32] DiMario RJ, Clayton H, Mukherjee A, Ludwig M, Moroney JV (2017). Plant carbonic anhydrases: structures, locations, evolution, and physiological roles. Mol. Plant.

[33] So AK, John-McKay M, Espie GS (2002). Characterization of a mutant lacking carboxysomal carbonic anhydrase from the cyanobacterium *Synechocystis* PCC6803. Planta.

[34] Nishimura T, Yamaguchi O, Takatani N, Maeda S, Omata T (2014). In vitro and in vivo analyses of the role of the carboxysomal β-type carbonic anhydrase of the cyanobacterium *Synechococcus elongatus* in carboxylation of ribulose-1,5-bisphosphate. Photosynth. Res..

[35] Price GD, Coleman JR, Badger MR (1992). Association of carbonic anhydrase activity with carboxysomes isolated from the cyanobacterium *Synechococcus* PCC7942. Plant Physiol..

[36] Li P (2012). Phase transitions in the assembly of multivalent signalling proteins. Nature.

[37] Mackinder LC (2016). A repeat protein links Rubisco to form the eukaryotic carbon-concentrating organelle. Proc. Natl Acad. Sci. USA.

[38] Banani SF, Lee HO, Hyman AA, Rosen MK (2017). Biomolecular condensates: organizers of cellular biochemistry. Nat. Rev. Mol. Cell Biol..

[39] Boeynaems S (2018). Protein phase separation: a new phase in cell biology. Trends Cell Biol..

[40] Choi JM, Holehouse AS, Pappu RV (2020). Physical principles underlying the complex biology of intracellular phase transitions. Annu. Rev. Biophys..

[41] McGurn LD (2016). The structure, kinetics and interactions of the beta-carboxysomal beta-carbonic anhydrase, CcaA. Biochem. J..

[42] Kimber MS, Coleman JR, Pai EF (2000). Beta-carbonic anhydrase from *Pisum sativum*: crystallization and preliminary X-ray analysis. Acta Crystallogr. D. Biol. Crystallogr..

[43] Aggarwal M, Chua TK, Pinard MA, Szebenyi DM, McKenna R (2015). Carbon dioxide “trapped” in a β-carbonic anhydrase. Biochemistry.

[44] Schlicker C (2009). Structure and inhibition of the CO_2_-sensing carbonic anhydrase Can2 from the pathogenic fungus *Cryptococcus neoformans*. J. Mol. Biol..

[45] Cronk JD, Endrizzi JA, Cronk MR, O’neill JW, Zhang KYJ (2001). Crystal structure of *E. coli* β-carbonic anhydrase, an enzyme with an unusual pH–dependent activity. Protein Sci..

[46] Ghosh A, Zhou H-X (2020). Determinants for fusion speed of biomolecular droplets. Angew. Chem. Int. Ed. Engl..

[47] So AKC, Cot SSW, Espie GS (2002). Characterization of the C-terminal extension of carboxysomal carbonic anhydrase from *Synechocystis* sp. PCC6803. Funct. Plant Biol..

[48] Krissinel E, Henrick K (2007). Inference of macromolecular assemblies from crystalline state. J. Mol. Biol..

[49] Fletcher JM (2012). A basis set of de novo coiled-coil peptide oligomers for rational protein design and synthetic biology. ACS Synth. Biol..

[50] Bienz M (2020). Head-to-tail polymerization in the assembly of biomolecular condensates. Cell.

[51] Rohnke BA, Kerfeld CA, Montgomery BL (2020). Binding options for the small subunit-like domain of cyanobacteria to Rubisco. Front. Microbiol..

[52] Saschenbrecker S (2007). Structure and function of RbcX, an assembly chaperone for hexadecameric Rubisco. Cell.

[53] Bracher A, Starling-Windhof A, Hartl FU, Hayer-Hartl M (2011). Crystal structure of a chaperone-bound assembly intermediate of form I Rubisco. Nat. Struct. Mol. Biol..

[54] Hauser T (2015). Structure and mechanism of the Rubisco-assembly chaperone Raf1. Nat. Struct. Mol. Biol..

[55] Cameron JC, Wilson SC, Bernstein SL, Kerfeld CA (2013). Biogenesis of a bacterial organelle: the carboxysome assembly pathway. Cell.

[56] Sun Y, Wollman AJM, Huang F, Leake MC, Liu LN (2019). Single-organelle quantification reveals stoichiometric and structural variability of carboxysomes dependent on the environment. Plant Cell.

[57] Wu H, Fuxreiter M (2016). The structure and dynamics of higher-order assemblies: amyloids, signalosomes, and granules. Cell.

[58] Catanzariti AM, Soboleva TA, Jans DA, Board PG, Baker RT (2004). An efficient system for high-level expression and easy purification of authentic recombinant proteins. Protein Sci..

[59] Baker, R. T. et al. in *Methods in Enzymology* Vol. 398 (ed. Deshaies, R. J.) 540–554 (Academic Press, 2005).

[60] Liu C (2010). Coupled chaperone action in folding and assembly of hexadecameric Rubisco. Nature.

[61] Brinker A (2001). Dual function of protein confinement in chaperonin-assisted protein folding. Cell.

[62] Schindelin J (2012). Fiji: an open-source platform for biological-image analysis. Nat. Methods.

[63] Wyatt PJ (1993). Light scattering and the absolute characterization of macromolecules. Anal. Chim. Acta.

[64] Vonrhein C (2011). Data processing and analysis with the autoPROC toolbox. Acta Crystallogr. D. Biol. Crystallogr..

[65] Kabsch W (2010). XDS. Acta Crystallogr. D. Biol. Crystallogr..

[66] Evans P (2006). Scaling and assessment of data quality. Acta Crystallogr. D. Biol. Crystallogr..

[67] Evans PR, Murshudov GN (2013). How good are my data and what is the resolution?. Acta Crystallogr. D. Biol. Crystallogr..

[68] Vagin A, Teplyakov A (2010). Molecular replacement with MOLREP. Acta Crystallogr. D. Biol. Crystallogr..

[69] Emsley P, Cowtan K (2004). Coot: model-building tools for molecular graphics. Acta Crystallogr. D. Biol. Crystallogr..

[70] Potterton E, Briggs P, Turkenburg M, Dodson E (2003). A graphical user interface to the CCP4 program suite. Acta Crystallogr. D. Biol. Crystallogr..

[71] Murshudov GN (2011). REFMAC5 for the refinement of macromolecular crystal structures. Acta Crystallogr. D. Biol. Crystallogr..

[72] Chen VB (2010). MolProbity: all-atom structure validation for macromolecular crystallography. Acta Crystallogr. D. Biol. Crystallogr..

[73] Kleywegt GT, Jones TA (1994). A super position. CCP4/ESF-EACBM Newslett. Protein Crystallogr..

[74] Mastronarde DN (2005). Automated electron microscope tomography using robust prediction of specimen movements. J. Struct. Biol..

[75] Zheng SQ (2017). MotionCor2: anisotropic correction of beam-induced motion for improved cryo-electron microscopy. Nat. Methods.

[76] Rohou A, Grigorieff N (2015). CTFFIND4: fast and accurate defocus estimation from electron micrographs. J. Struct. Biol..

[77] Biyani N (2017). Focus: the interface between data collection and data processing in cryo-EM. J. Struct. Biol..

[78] Scheres SH (2012). RELION: implementation of a Bayesian approach to cryo-EM structure determination. J. Struct. Biol..

[79] Pettersen EF (2004). UCSF Chimera—a visualization system for research and analysis. J. Comput. Chem..

[80] Ashkenazy H (2016). ConSurf 2016: an improved methodology to estimate and visualize evolutionary conservation in macromolecules. Nucleic Acids Res..

[81] Eddy SR (2009). A new generation of homology search tools based on probabilistic inference. Genome Inform..

[82] Katoh K, Standley DM (2013). MAFFT multiple sequence alignment software version 7: improvements in performance and usability. Mol. Biol. Evol..

[83] Crooks GE, Hon G, Chandonia JM, Brenner SE (2004). WebLogo: a sequence logo generator. Genome Res..

