# Supplementary material for: Scaffolding protein CcmM directs multiprotein phase separation in β-carboxysome biogenesis
Source: Nat Struct Mol Biol. 2021 Nov 10;28(11):909–22. doi: 10.1038/s41594-021-00676-5 (PMC8580825; doi:10.1038/s41594-021-00676-5)
Supplement: Supplementary file 1 — Supplementary Tables 1, 2 and 4 and legend for Table 3 (Excel), named Zang_Wang et al_Supplementary Info_NSMB-A44989-R2.xlsx (see below). [file 41594_2021_676_MOESM1_ESM.pdf]

nature portfolio

https://doi.org/10.1038/s41594-021-00676-5

Supplementary information

Scaffolding protein CcmM directs multiprotein phase separation in β-carboxysome biogenesis

In the format provided by the authors and unedited

Supplementary Tables

Supplementary Table 1 | Molar mass and hydrodynamic radius of proteins determined by SEC-MALS.

Protein	Measured Molar mass (Da)	Theoretical MW (Da)		Hydrodynamic radius (nm)
		monomer	oligomer	
M35	~33840	37158.29	-----	~3.1
$CC_{TRI}M35$	~101600	40375.11	121125.33	~5.1
CcaA	~127000*	30185.36	120741.44	~5.6
CcaAΔC2	~114000*	28472.47	113889.88	~5.2
CcaA(W257A)	~130800*	30070.23	120280.92	~5.7
CcaA(R265D)	~129400*	30144.26	120577.04	~5.7
E_{GFP}	~23990*	27286.79	-----	~2.1
E_{GFP}-$C2_{15}$	~30960*	28999.68	-----	~2.0
E_{GFP}-$C2_{17}$	~29540*	29242.94	-----	~2.7
E_{GFP}-$C2_{17}$(W257A)	~29360*	29127.81	-----	~2.7
E_{GFP}-$C2_{17}$(R265D)	~29120*	29201.85	-----	~2.7
M58	~156900	57832.84	173498.52	~6.3
M58-C4S	~153600	57768.60	173305.80	~6.1
γCAL-2S	~125600	46303.89	138911.67	~5.1
γCAL-1S	~95190	33764.01	101292.03	~4.6
γCAL_{198}	~67050*	20976.88	62930.62	~3.7
γCAL_{181}	~61160*	19228.91	57686.73	~3.4
M58(E17K)	~155800	57831.90	173495.70	~6.2
M58(D21K)	~154800	57845.93	173537.79	~6.1
M58(D35K)	~152100	57845.93	173537.79	~6.2
M58(R37D)	~153700	57791.75	173375.25	~6.2
M58(R43D)	~152400	57791.75	173375.25	~6.2
M58(K62D)	~154300	57819.76	173459.28	~6.2
M58(E76K)	~152900	57831.90	173495.70	~6.2
M58(R79D)	~155800	57791.75	173375.25	~6.3
M58(R95D)	~154100	57791.75	173375.25	~6.2
M58(D112K)	~154900	57845.93	173537.79	~6.1
M58(R126D)	~159500	57791.75	173375.25	~6.3
M58(R164D)	~150500	57791.75	173375.25	~6.2
M58(D172K)	~151600	57845.93	173537.79	~6.2
M58(E246K)	~149200	57831.90	173495.70	~6.1
M58(D249K)	~155800	57845.93	173537.79	~6.1
M58(R251D)	~149000	57791.75	173375.25	~6.1
M58(R252D)	~147700	57791.75	173375.25	~6.1
M58(E286K)	~158000	57831.90	173495.7	~6.2
M58(D294K)	~157400	57845.93	173537.79	~6.1
M58(R298D)	~156300	57791.75	173375.25	~6.3
M58(E303K)	~158300	57831.90	173495.7	~6.2
M58(R367D)	~154400	57791.75	173375.25	~6.3
M58(R481D)	~153600	57791.75	173375.25	~6.3

*Measured in buffer 50 mM Tris-HCl pH 8.0/150 mM KCl.
All other proteins measured in buffer 50 mM Tris-HCl pH 8.0/500 mM KCl/5 mM DTT.

Supplementary Table 2 | Size distribution of condensates.

Sample	Average Feret's diameter (μm)
0.25 μM $M58_{red}$/0.25 μM CcaA	~1.2
0.25 μM $M58_{ox}$/0.25 μM CcaA	~1.0
0.25 μM Rubisco/0.25 μM $M58_{red}$	~1.6
0.25 μM Rubisco/0.25 μM $M58_{red}$/0.25 μM CcaA	~1.5
0.5 μM Rubisco/2.0 μM $M35_{red}$/0.25 μM $M58_{red}$/0.25 μM CcaA	~2.25
0.5 μM Rubisco/2.0 μM $M35_{red}$/0.25 μM $CC_{TRI}M35_{red}$/0.25 μM CcaA	~2.6

Supplementary Table 3 | Oligo sequences used in this study.

See separate Excel spreadsheet.

Supplementary Table 4 | Plasmids generated or used in this study.

Plasmid/Strain	Protein/Strain	Source
pHUE-*Se*M58	*Se*M58	This study
pHUE-*Se*M58(E17K)	M58(E17K)	This study
pHUE-*Se*M58(D21K)	M58(D21K)	This study
pHUE-*Se*M58(D35K)	M58(D35K)	This study
pHUE-*Se*M58(R37D)	M58(R37D)	This study
pHUE-*Se*M58(R43D)	M58(R43D)	This study
pHUE-*Se*M58(K62D)	M58(K62D)	This study
pHUE-*Se*M58(E76K)	M58(E76K)	This study
pHUE-*Se*M58(R79D)	M58(R79D)	This study
pHUE-*Se*M58(R95D)	M58(R95D)	This study
pHUE-*Se*M58(D112K)	M58(D112K)	This study
pHUE-*Se*M58(R126D)	M58(R126D)	This study
pHUE-*Se*M58(R164D)	M58(R164D)	This study
pHUE-*Se*M58(D172K)	M58(D172K)	This study
pHUE-*Se*M58(E246K)	M58(E246K)	This study
pHUE-*Se*M58(D249K)	M58(D249K)	This study
pHUE-*Se*M58(R251D)	M58(R251D)	This study
pHUE-*Se*M58(R252D)	M58(R252D)	This study
pHUE-*Se*M58(E286K)	M58(E286K)	This study
pHUE-*Se*M58(D294K)	M58(D294K)	This study
pHUE-*Se*M58(R298D)	M58(R298D)	This study
pHUE-*Se*M58(E303K)	M58(E303K)	This study
pHUE-*Se*M58(R367D)	M58(R367D)	This study
pHUE-*Se*M58(R481D)	M58(R481D)	This study
pHUE-*Se*M58-C4S (C261S/C279S/C377S/C395S)	M58-C4S	This study
pHUE-CC_{TRI}M35	CC_{TRI}M35	This study
pHUE-*Se*γCAL-2S(1-429)	γCAL-2S	This study
pHUE-*Se*γCAL-1S(1-313)	γCAL-1S	This study
pHUE-*Se*γCAL(1-198)	*Se*γCAL_{198}	This study
pHUE-*Se*γCAL(1-181)	*Se*γCAL_{181}	This study
pHUE-*Se*CcaA	*Se*CcaA	This study
pHUE-*Se*CcaAΔC2	*Se*CcaAΔC2	This study
pHUE-*Se*CcaA(W257A)	*Se*CcaA(W257A)	This study
pHUE-*Se*CcaA(R265D)	*Se*CcaA(R265D)	This study
pHUE-$C2_{17}$	$C2_{17}$	This study
pHUE-E_{GFP}	E_{GFP}	This study
pHUE-$E_{GFP}C2_{15}$	$E_{GFP}C2_{15}$	This study
pHUE-$E_{GFP}C2_{17}$	$E_{GFP}C2_{17}$	This study
pHUE-$E_{GFP}C2_{17}$(W257A)	$E_{GFP}C2_{17}$(W257A)	This study
pHUE-$E_{GFP}C2_{17}$(R265D)	$E_{GFP}C2_{17}$(R265D)	This study
pHUE_ *Syn*6301_*ccmM*_M35	M35	Ref. 16
pET11a_ *Syn*6301_*rbcL*	$RbcL_8$	Ref. 52
pET11a_*Syn*6301_*rbcS*	RbcS	Ref. 52
pHUE-Se7942_*raf1*	SeRaf1	Ref. 52
pEF-*gfp*	E_{GFP}	Addgene Cat#11154
