# Supplementary material for: Scaffolding protein CcmM directs multiprotein phase separation in β-carboxysome biogenesis
Source: Nat Struct Mol Biol. 2021 Nov 10;28(11):909–22. doi: 10.1038/s41594-021-00676-5 (PMC8580825; doi:10.1038/s41594-021-00676-5)
Supplement: Supplementary file 2 — Reporting Summary [file 41594_2021_676_MOESM2_ESM.pdf]

nature research

Corresponding author(s): Manajit Hayer-Hartl

Last updated by author(s): Sep 20, 2021

Reporting Summary

Nature Research wishes to improve the reproducibility of the work that we publish. This form provides structure for consistency and transparency in reporting. For further information on Nature Research policies, see our Editorial Policies and the Editorial Policy Checklist.

Statistics

For all statistical analyses, confirm that the following items are present in the figure legend, table legend, main text, or Methods section.

n/a	Confirmed	
☐	☒	The exact sample size (*n*) for each experimental group/condition, given as a discrete number and unit of measurement
☐	☒	A statement on whether measurements were taken from distinct samples or whether the same sample was measured repeatedly
☒	☐	The statistical test(s) used AND whether they are one- or two-sided<br>*Only common tests should be described solely by name; describe more complex techniques in the Methods section.*
☒	☐	A description of all covariates tested
☒	☐	A description of any assumptions or corrections, such as tests of normality and adjustment for multiple comparisons
☐	☒	A full description of the statistical parameters including central tendency (e.g. means) or other basic estimates (e.g. regression coefficient) AND variation (e.g. standard deviation) or associated estimates of uncertainty (e.g. confidence intervals)
☒	☐	For null hypothesis testing, the test statistic (e.g. *F*, *t*, *r*) with confidence intervals, effect sizes, degrees of freedom and *P* value noted<br>*Give P values as exact values whenever suitable.*
☒	☐	For Bayesian analysis, information on the choice of priors and Markov chain Monte Carlo settings
☒	☐	For hierarchical and complex designs, identification of the appropriate level for tests and full reporting of outcomes
☒	☐	Estimates of effect sizes (e.g. Cohen's *d*, Pearson's *r*), indicating how they were calculated

Our web collection on statistics for biologists contains articles on many of the points above.

Software and code

Policy information about availability of computer code

Data collection	MXCuBE3; SSX suite; SerialEM version 3.5; Focus v.1.0.0 (https://github.com/C-CINA/focus); Leica Application Suite X.
Data analysis	HMMER version 3.1b2; MAFFT version 7; autoPROC version 1.0.5 (XDS,POINTLESS, AIMLESS); CCP4 version 7.1 (MOLREP, Lsqkab, Lsqman); Coot version 0.9.3; REFMAC5 version 5.8.0267; PyMol (Schrödinger LLC version 2.3); MolProbity version 4.5.1; PISA version1.52; RELION 3.1.1; MotionCor2 version Relion implemented; Gautomatch version 0.56; CTFFIND version 4.1; Chimera version 1.12; DeepEMhancer version sep.2020; Fiji version Madison; ConSurf 2016; WebLogo version 2.8.2; Origin 2020; SigmaPlot 14.; ASTRA 5.

For manuscripts utilizing custom algorithms or software that are central to the research but not yet described in published literature, software must be made available to editors and reviewers. We strongly encourage code deposition in a community repository (e.g. GitHub). See the Nature Research guidelines for submitting code & software for further information.

Data

Policy information about availability of data

All manuscripts must include a data availability statement. This statement should provide the following information, where applicable:

- Accession codes, unique identifiers, or web links for publicly available datasets
- A list of figures that have associated raw data
- A description of any restrictions on data availability

The crystallographic structure factors and models for SegCAL(1-181) and SegCAL(1-181)-C217 complex have been deposited to the PDB database under accession codes 7O4Z and 7O54, respectively. The local electron density maps for SeM58ox, SeM58red-SeRubisco and SeM58red-SeRbcL8 are deposited under EMDB accession codes EMD-12730, EMD-12731 and EMD-12732, respectively. Source data for the following figures are provided with this paper: Figures 1d-f; 2b,d,e; 3d; 4a,b,d-f,j,k; 5a-c,g,h; 6f,g; Extended Data Figures 1b,c; 2a,b,e; 4a,c,e,f,h; 7a-d; 8a-c,h. Other data are available from corresponding author upon reasonable request.

Field-specific reporting

Please select the one below that is the best fit for your research. If you are not sure, read the appropriate sections before making your selection.

☒ Life sciences ☐ Behavioural & social sciences ☐ Ecological, evolutionary & environmental sciences

For a reference copy of the document with all sections, see nature.com/documents/nr-reporting-summary-flat.pdf

Life sciences study design

All studies must disclose on these points even when the disclosure is negative.

Sample size	All relevant biochemical experiments were replicated two or three times. No statistical methods were used to predetermine sample size, but our sample sizes are similar to those reported in previous publications (ref. 16, 19). For cryo-EM, data was screened on 8 independently prepared samples.
Data exclusions	No data were excluded.
Replication	All attempts at replication were successful. Crystals could be grow from at least two batches of protein. Diffraction resolution from crystals was variable. Cryo-EM single particle analysis inherently relies on averaging a large number of independent observations. All critical biochemical experiments were performed in independent duplicates or triplicates. Results shown in Extended Data Fig. 2d,f and Extended Data Fig. 10 were performed only once under the same conditions as for the repeat experiments which are highly reproducible.
Randomization	Samples were not allocated to groups. All cryo-EM particles for structure determination adopt random orientaions in the ice on the grid. Division of particles into random halves was automatically performed during 3D reconstruction by Relion 3.1. Other experiments did not involve randomization.
Blinding	Blinding is not relevant in this study as outcomes of biochemical experiments we performed are not affected by knowledge of the sample.

Reporting for specific materials, systems and methods

We require information from authors about some types of materials, experimental systems and methods used in many studies. Here, indicate whether each material, system or method listed is relevant to your study. If you are not sure if a list item applies to your research, read the appropriate section before selecting a response.

Materials & experimental systems

n/a	Involved in the study	
☒	☐	Antibodies
☒	☐	Eukaryotic cell lines
☒	☐	Palaeontology and archaeology
☒	☐	Animals and other organisms
☒	☐	Human research participants
☒	☐	Clinical data
☒	☐	Dual use research of concern

Methods

n/a	Involved in the study	
☒	☐	ChIP-seq
☒	☐	Flow cytometry
☒	☐	MRI-based neuroimaging
