# Supplementary figures and images for: Scaffolding protein CcmM directs multiprotein phase separation in β-carboxysome biogenesis
Source: Nat Struct Mol Biol. 2021 Nov 10;28(11):909–22. doi: 10.1038/s41594-021-00676-5 (PMC8580825; doi:10.1038/s41594-021-00676-5)

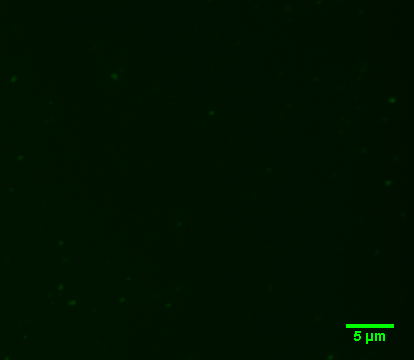

Supplement: Supplementary file 5 — Time-lapse video of condensates of M58red and CcaA. Condensates of M58red (0.25 μM) and CcaA (0.25 μM) in the presence of 100 mM KCl. M58red/AF5 fluorescence was detected. Scale bar, 5 μm. [file 41594_2021_676_MOESM5_ESM.gif]

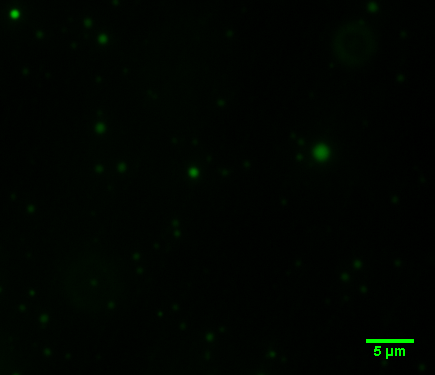

Supplement: Supplementary file 6 — Time-lapse video of condensates of M58ox. Condensate of M58ox (2.5 μM) in the presence of 50 mM KCl. M58ox/AF5 fluorescence was detected. Scale bar, 5 μm. [file 41594_2021_676_MOESM6_ESM.gif]

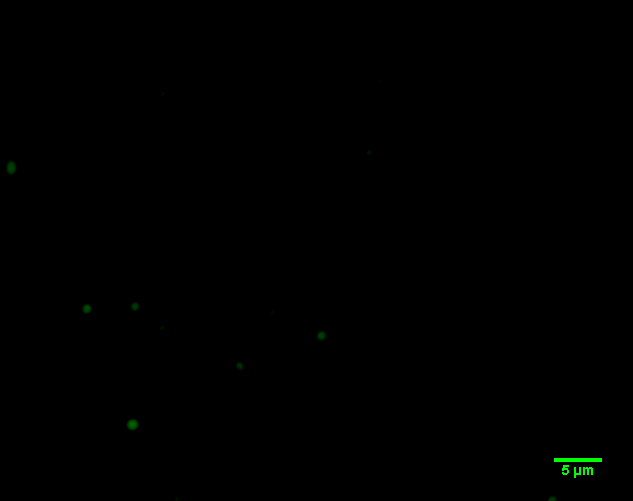

Supplement: Supplementary file 7 — Time-lapse video of condensates of M58red and Rubisco. Condensates of M58red (0.25 μM) and Rubisco (0.25 μM) in the presence of 100 mM KCl. M58red/AF5 fluorescence was detected. Scale bar, 5 μm. [file 41594_2021_676_MOESM7_ESM.gif]

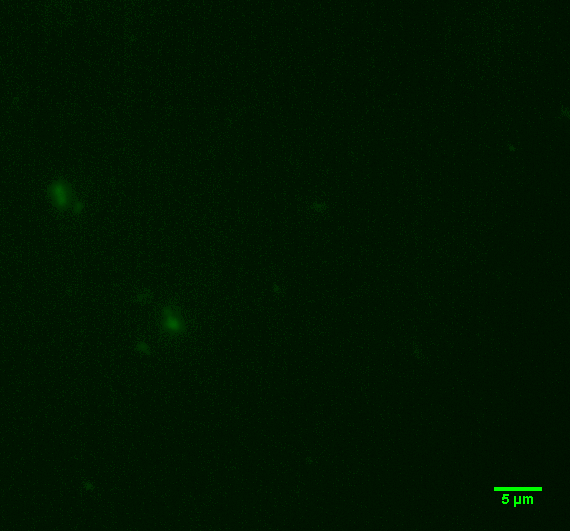

Supplement: Supplementary file 8 — Time-lapse video of condensates of M35red and Rubisco. Condensates of M35red (2.0 μM) and Rubisco (0.25 μM) in the presence of 50 mM KCl. M35red/AF5 fluorescence was detected. Scale bar, 5 μm. [file 41594_2021_676_MOESM8_ESM.gif]

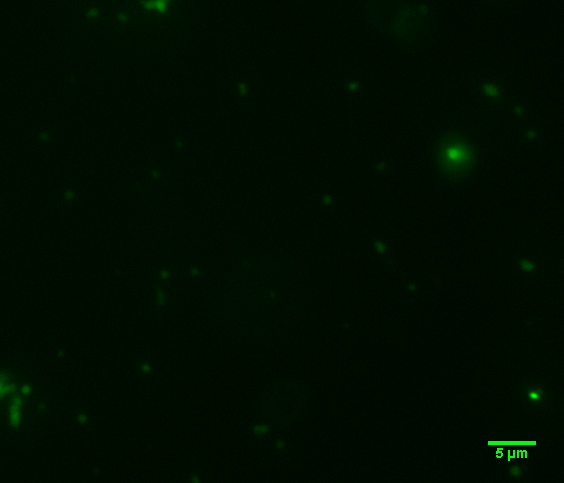

Supplement: Supplementary file 9 — Time-lapse video of four-protein condensate. Four-protein condensates (0.5 μM Rubisco/2 μM M35red/0.25 μM M58red/0.25 μM CcaA) in the presence of 100 mM KCl. M58red/AF5 fluorescence was detected. Scale bar, 5 μm. [file 41594_2021_676_MOESM9_ESM.gif]
