# Supplementary material for: Scaffolding protein CcmM directs multiprotein phase separation in β-carboxysome biogenesis
Source: Nat Struct Mol Biol. 2021 Nov 10;28(11):909–22. doi: 10.1038/s41594-021-00676-5 (PMC8580825; doi:10.1038/s41594-021-00676-5)
Supplement: Supplementary file 3 — Peer Review Information [file 41594_2021_676_MOESM3_ESM.pdf]

natureresearch

Peer Review Information

Journal: Nature Structural and Molecular Biology
Manuscript Title: Scaffolding Protein CcmM Directs Multiprotein Phase Separation in β-Carboxysome Biogenesis
Corresponding author name(s): Dr Manajit Hayer-Hartl

Reviewer Comments & Decisions:

Decision Letter, initial version:

30th Jun 2021

Dear Manajit,

Thank you again for submitting your manuscript "Scaffolding Protein CcmM Directs Multiprotein Phase Separation in β-Carboxysome Biogenesis". I apologize again for the delay in responding, which resulted from the difficulty in obtaining suitable referee reports and the other issue I had mentioned to you. Nevertheless, we now have comments (below) from the 3 reviewers who evaluated your paper. In light of those reports, we remain interested in your study and would like to see your response to the comments of the referees, in the form of a revised manuscript.

You will see that overall, the reviewers are positive about the quality and interest of the work, with reviewer #2 being particularly supportive. However, additional experiments were requested. Reviewer #1, an expert in protein liquid-liquid phase separation, makes suggestions to improve this aspect of the study. Reviewer #3 requests confirmation of several conclusions with new data. In addition, all reviewers have useful suggestions to improve the presentation and discussion of the findings. Please be sure to address/respond to all concerns of the referees in full in a point-by-point response and highlight all changes in the revised manuscript text file.

We appreciate the requested revisions are extensive and that addressing the referee requests in full might be challenging. Please contact me if you would like to discuss a plan for the revision. We are committed to providing a fair and constructive peer-review process. Do not hesitate to get in touch with us if there are specific requests from the reviewers that you believe are technically impossible or unlikely to yield a meaningful outcome.

<u>We expect to see your revised manuscript within 6 months.</u> If you cannot send it within this time, please let us know. We will be happy to consider your revision as long as nothing similar has been accepted for publication at NSMB or published elsewhere. Should your manuscript be substantially delayed without notifying us in advance and your article is eventually published, the received date would be that of the revised, not the original, version.

***

As you already know, we put great emphasis on ensuring that the methods and statistics reported in our papers are correct and accurate. As such, if there are any changes that should be reported, please submit an updated version of the Reporting Summary along with your revision.

Reporting Summary:
https://www.nature.com/documents/nr-reporting-summary.pdf

Please note that the form is a dynamic 'smart pdf' and must therefore be downloaded and completed in Adobe Reader.

When submitting the revised version of your manuscript, please pay close attention to our href="https://www.nature.com/nature-research/editorial-policies/image-integrity">Digital Image Integrity Guidelines.

Finally, please ensure that you retain unprocessed data and metadata files after publication, ideally archiving data in perpetuity, as these may be requested during the peer review and production process or after publication if any issues arise.

If there are additional or modified structures presented in the final revision, please submit the corresponding PDB validation reports.

SOURCE DATA: we urge authors to provide, in tabular form, the data underlying the graphical representations used in figures. This is to further increase transparency in data reporting, as detailed in this editorial (http://www.nature.com/nsmb/journal/v22/n10/full/nsmb.3110.html). Spreadsheets can be submitted in excel format. Only one (1) file per figure is permitted; thus, for multi-paneled figures, the source data for each panel should be clearly labeled in the Excel file; alternately the data can be provided as multiple, clearly labeled sheets in an Excel file. When submitting files, the title field should indicate which figure the source data pertains to. We encourage our authors to provide source data at the revision stage, so that they are part of the peer-review process.

While we encourage the use of color in preparing figures, please note that this will incur a charge to partially defray the cost of printing. Information about color charges can be found at http://www.nature.com/nsmb/authors/submit/index.html#costs

We require deposition of coordinates (and, in the case of crystal structures, structure factors) into the Protein Data Bank with the designation of immediate release upon publication (HPUB). Electron microscopy-derived density maps and coordinate data must be deposited in EMDB and released upon publication. Deposition and immediate release of NMR chemical shift assignments are highly encouraged. Deposition of deep sequencing and microarray data is mandatory, and the datasets must be released prior to or upon publication. To avoid delays in publication, dataset accession numbers must be supplied with the final accepted manuscript and appropriate release dates must be indicated at the galley proof stage. Please find the complete NRG policies on data availability at http://www.nature.com/authors/policies/availability.html.

Nature Structural & Molecular Biology is committed to improving transparency in authorship. As part of our efforts in this direction, we are now requesting that all authors identified as 'corresponding author' on published papers create and link their Open Researcher and Contributor Identifier (ORCID) with their account on the Manuscript Tracking System (MTS), prior to acceptance. This applies to primary research papers only. ORCID helps the scientific community achieve unambiguous attribution of all scholarly contributions. You can create and link your ORCID from the home page of the MTS by clicking on 'Modify my Springer Nature account'. For more information please visit please visit www.springernature.com/orcid.

Please use the link below to submit your revised manuscript and related files:

[REDACTED]

Note: This URL links to your confidential home page and associated information about manuscripts you may have submitted, or that you are reviewing for us. If you wish to forward this email to co-authors, please delete the link to your homepage.

We look forward to seeing the revised manuscript and thank you for the opportunity to review your work.

Kind regards,
Florian

Florian Ullrich, Ph.D.
Associate Editor

Nature Structural & Molecular Biology
ORCID 0000-0002-1153-2040

Referee expertise:

Referee #1: protein phase separation

Referee #2: photosynthesis, carboxysomes

Referee #3: plant structural biology

Reviewers' Comments:

Reviewer #1:
Remarks to the Author:
The paper provides insights into the molecular interactions underlying of the complex network of Rubisco, leading to condensate formation. While some of the details are highly elaborated, several key issues remained to be elusive regarding the material state of the assembly, the connection between dynamics and condensate formation, the role of motif-flanking regions in mediating multivalent interactions. The paper would also benefit from relating the findings to other relevant papers in the field, regarding Rubisco multivalency, condensate formation via head-to-tail mechanisms, thermodynamics and material states with multivalent higher-order systems, as listed below.

- In the abstract, please point out the key feature of gCAL(CcmM), which enables network formation
- Please provide schematic representation of the different domains
- Please introduce condensates and multivalent interactions in intro (PMID 22398450, 29602697)
- What is the role of condensate formation in Rubisco?

- Results: provide more details on the material state of condensates (gel, amyloid -like etc.) based on experimental data
- Results: it would be useful to support the analysis on C2 motif by bioinformatics predictions to see the interplay between hydrophobic interactions and protein dynamics (PMID 33318217)
in particular, that a disordered motif drives formation of a non-mobile association
- Here the critical concentration is ~micromol, and the interaction affinity is also in the same order of magnitude. In most multivalent systems, the individual motifs are ~mM (see PMID 27203110). Can you comment on this?
- How the multivalent interactions seen here relate to those in ref 16?

- Results: can you provide the KD for the full protein to see how much the flanking regions contribute to interactions?
- Results: in the structure part the direct link between the observed interactions and multivalency is not clear
- What is the role of disordered flanking regions and protein dynamics? Cryo-EM indicates protruding densities, which might be critical for the network assembly .
- The role of dynamics is not clear in the discussion it is mentioned as more dynamical state, in the results as a not dynamic association. Is it possible that salt concentration also affects the dynamics of protein chains?

References
- cite head-to-tail polymerisation as a mechanism for phase separation (PMID 32822572) or for less dynamic assemblies (PMID 27203110)

Reviewer #2:
Remarks to the Author:
Summary of the key results:
The proteinaceous microcompartments known as carboxysomes found in cyanobacteria have been a great interest to the photosynthesis research community because these bacterial organelles are highly effective in concentrating CO_2 around Rubisco. In β-carboxysomes, the main scaffolding protein, CcmM, is known to function as two isoforms: M58, which is the full-length version, and M35, which is the shorter version with only the C-terminal part of M58. Previous studies showed that both isoforms are essential components of β-carboxysomes. M35 is made up of three repeats of Rubisco small subunit-like (SSUL) modules, and previous work (Wang et al, Nature, 566, 131-135, 2019) detailed how it forms condensate with Rubisco through structural and biochemical methods. The N-terminal part of M58 contains a γ carbonic anhydrase-like domain (γ-CAL) and has been known to interact with at least two other proteins of β-carboxysomes, CcmN and βCA. However, the detailed mechanism of the interecations between M58 and other proteins are not known. In this manuscript, Zang et al describe their findings from biochemical and structural studies of M58, βCA, Rubisco and M35. They found that M58 is able to form three different types of condensates: with βCA, Rubisco or by itself. The biochemical and structural experiments are well designed and clearly presented in the manuscript. Not only did they address the critical interaction between M58 and βCA at the atomic levels, they also revealed two unexpected interactions: one between the SSUL modules and γ-CAL domain of M58 and the head-to-head interaction between γ-CAL domains from two M58 trimers. All these interactions lead to intricate network of proteins influenced by redox conditions. Hence, this manuscript is an important step towards understanding the detailed interactions among proteins inside β-carboxysomes and should be of great

interest to the researchers studying β-carboxysomes as well as implementing them into plants for bioengineering purposes. Despite the intricate nature of the materials, the manuscript is well organized, and the findings are clearly described.

Minor suggestions for the authors:
1. In line 202, when homo-demixing of M58 in Fig. 4c,d was described, it would be helpful to point out the difference betweeen the experiments in Fig. 4c,d and those in Fig. 1b,c where M58 alone was shown not to form condensate.
2. In Fig 8a ii, shouldn't the γCAL-CcaA condensate be salt "insensitive" based on Fig 2e?
3. The paper from Gonzalez-Esquer et al (Streamlined construction of the cyanobacterial CO2-fixing organelle via protein domain fusions for use in plant synthetic biology, The Plant Cell, 27, 2637-2644, 2015) previously showed that a single chimeric protein was able to structurally and functionally replace four proteins: M58, M35, βCA and CcmN. The chimeric protein was derived by replacing γCAL in M58 with a shorter version of βCA. It was not understood how the chimeric protein was able to replace both M58 and M35. Would the new findings in the current manuscript somehow provide some 'speculative' explanation regarding the unusual behavior of that chimeric protein? For example, the current manuscript suggests there is differential redox-regulation of the interactions between Rubisco and M58 versus M35. More specifically, the interaction between SSUL modules and γCAL in M58, which appeared enhanced under oxidizing conditions, is no longer present with the chimeric protein. Could the redox regulation of the chimeric protein be more similar to M35, while at the same time, it is more structurally and physically similar to M58? Can the authors provide some insights into the chimeric protein in Gonzalez-Esquer et al paper in the discussion section?

Reviewer #3:
Remarks to the Author:
Carboxysome is a special microcompartment formed in cyanobacteria and algae that enclose the enzymes Rubisco and carbonic anhydrase to optimize photosynthetic carbon fixation. Understanding the biogenesis of carboxysome assembly and implementing an optimized carboxysome-like CO2 concentrating mechanism (CCM) into plants is a desirable strategy to increase the crop yield. In this study, the authors revealed a central role of the scaffolding protein CcmM M58 via initiating phase separation in the biogenesis of β-carboxysomes in cyanobacteria. Specially, the N-terminal γCAL domain of M58 mediate interactions with CcaA (support the CA activity), intermolecular interactions with the SSUL domain, and also head-to-head association of CcmM trimers. Overall, this study provides new sights into the biogenesis of β-carboxysome. I have a few concerns on the manuscript.
1. In figure 2f and 3, it seems that W257 in the C2 peptide play crucial roles in mediating the interactions with the γCAL domain of M58. Further biochemical assays should be carried out to validate the structural model as well as the key involved residues.

2. CcaA exhibits as a tetramer in solution (Fig S1). It seems the preceding C1 peptide mediates the oligmerization. In the complex structure of γCAL-C2, each protomer of γCAL binds a C2 peptide. The conclusion “each protomer of the γCAL trimer interacts with one protomer of a CcaA tetramer” should be biochemically validated using full length proteins.
3. In Supplemental Fig 6, for the 3.6 Å resolution map, the color gradient of the local resolution maps of side and end views from blue to red indicates local resolution from 2.0 to 6.0 Å. I suggest the authors show the values in a shorter range (3.0 to 6.0 Å?).
4. In Supplemental Fig 8, the authors showed the structural model of M58-RbcL8 to further validate the interaction between M58 and Rubisco complex. As the resolution of M58-RbcL8 is only 8 Å, information provided from the model is very limited and not reliable. I think this part is not necessary in the current manuscript.
5. In supplementary Table 3, the authors should provide more parameters to show the model validations.
6. In figures 1, 4, 7 and supplementary figs 2, 4, 10, the authors show the condensates of different proteins. In some figures, the authors exhibited the FRAP assays. It will be better to additionally show the results of droplet fusion.
7. The authors demonstrated that Rubisco, M58, M35 and CcaA could co-assembly in vitro. How about in vivo? It will be better to confirm the co-assembly in vivo.
8. In figure 6, the authors demonstrated that trimeric M58 binds rubisco with high affinity in a turbidity assay. Maybe some additional assays such as ITC will be better.
9. CcmM directs multiprotein phase separation in β-carboxysome biogenesis. How about the effect of this phase separation on carbon fixation? Whether the ability of β-carboxysome carbon fixation depends on this phase separation?

Author Rebuttal to Initial comments

Reviewer #1 (phase separation)
Remarks to the Author:

The paper provides insights into the molecular interactions underlying of the complex network of Rubisco, leading to condensate formation. While some of the details are highly elaborated, several key issues remained to be elusive regarding the material state of the assembly, the connection between dynamics and condensate formation, the role of motif-flanking regions in mediating multivalent interactions. The paper would also benefit from relating the findings to other relevant papers in the field, regarding Rubisco multivalency, condensate formation via head-to-tail mechanisms, thermodynamics and material states with multivalent higher-order systems, as listed below.

Thank you for focusing our attention on these points. We agree that a more extensive discussion of our

findings regarding Rubisco in light of the known biophysical principles of condensate formation will make our study more attractive for a broad readership. We have amended the Introduction and Discussion sections accordingly.

- In the abstract, please point out the key feature of gCAL(CcmM), which enables network formation
We have rewritten the abstract to emphasize the role of the γCAL domains of M58 in sequestering Rubisco and CcaA via multivalent interactions.

- Please provide schematic representation of the different domains. "When you read this paper being not from the RUbisco field, it is difficult to capture the jargon of names. I had difficulties synchronizing the names with the text, in particular in this format. I think some names appear before their scheme.

Following the reviewer's suggestion, we have added the structure of Rubisco and schematics of M58. M35 and CcaA in Fig. 1.

- Please introduce condensates and multivalent interactions in intro (PMID 22398450, 29602697)

We have done as suggested, and have cited these and other papers.

- What is the role of condensate formation in Rubisco?

Condensate formation of Rubisco, CcaA and other proteins, involved in carbon fixation, is the first step in the biogenesis of carboxysomes. This is mediated by the scaffolding protein CcmM (M58 and M35 in Synechococcus elongatus PCC 7942). Once the carboxysome proteinaneous shell has formed, the material property of the condensate is no longer critical to maintain the compartment. Thus, metabolic compartments, including carboxysomes, differ from membraneless condensates, such as stress granules or the nucleolus which do not have a shell and are dissociable. In fact, some of the multivalent interactions in the pre-carboxysome condensate are weakened (Wang H et al., Nature 2019) after shell formation, as the interior of the carboxysome is oxidizing (Price GD et al., Plant Physiol. 1992; Peña KL et al., PNAS 2010; Chen AH et al., PLoS One 2013). These aspects are now discussed in more detail.

- Results: provide more details on the material state of condensates (gel, amyloid -like etc.) based on experimental data

By microscopic observation the condensate is viscous, but it is not a "hydro-gel" or amyloid-like aggregate. Consistent with a state of low liquidity, the droplets of the 4-protein condensate (Rubisco, M58, M35 and CcaA) undergo slow fusion. This is now shown in new Fig. 7e and Supplementary Movie 5. Moreover, Rubisco in the condensate remains fully enzymatically active (new Fig. 7g), excluding an amyloid/fibrillar state.

- Results: it would be useful to support the analysis on C2 motif by bioinformatics predictions to see the interplay between hydrophobic interactions and protein dynamics (PMID 33318217) in particular, that a disordered motif drives formation of a non-mobile association

The FuzDrop method developed by the group of Vendruscolo (Hardenberg M et al., PNAS 2020) is based on the assumption that a droplet state is stabilized by non-specific or low-specific side-chain interactions. This does not apply to the C2-γCAL interaction, which is based on specific interactions with a welldefined interface, as seen in the crystal structure. The C2 motif binds as a two-turn helix. We now show that mutating Trp257 to Ala and Arg265 to Asp in the C2 sequence of CcaA reduced condensate formation with M58 by ~50%, or completely abolished condensate formation, respectively, as analyzed in the turbidity assay (see new Fig. 3d). Furthermore, both mutations in EGFPC17 abolished complex formation with γCAL by gel-shift assay (see new Supplementary Fig. 3i). These results underscore the specific contribution of hydrophobic and charge interactions to binding as indicated by the crystal structure.

- Here the critical concentration is ~micromol, and the interaction affinity is also in the same order of magnitude. In most multivalent systems, the individual motifs are ~mM (see PMID 27203110). Can you comment on this?

Thank you for pointing this out. As explained above, the interactions involved in Rubisco/CcaA condensation by CcmM rely on specific interactions and occur with relatively high affinity compared to the more fluctuating interactions underlying the formation of membraneless condensates. This makes sense in order to ensure that Rubisco and CcaA are concentrated with high efficiency, limiting escape and ensuring efficient encapsulation upon shell formation. This is now emphasized in the revised manuscript.

- How the multivalent interactions seen here relate to those in ref 16?

There are interesting differences and similarities between the Rubisco-CsoS2 and the Rubisco-CcmM interactions. The main difference is that in case of CsoS2 the interaction is mediated by 4 disordered

repeat motifs, while the SSUL modules of M58/M35 interact as folded domains. Multivalency is critical in both interactions and is highly efficient in capturing Rubisco, involving similar binding regions on Rubisco. Given the space limitations, a detailed discussion of the paper by Oltrogge et al. would need to be deferred to a future review article. However, we have inserted a statement that α-carboxysomes use intrinsically disordered, linear motifs as phase separation scaffold.

- Results: can you provide the KD for the full protein to see how much the flanking regions contribute to interactions?

The apparent KD values determined by turbidity assay for the interactions of the full proteins, previously in the text, are now shown in Fig. 1f and Fig. 6a. Note that ITC measurements are not possible for condensate forming reactions.
The flanking regions flexibly linking the SSUL modules show no conserved motif or sequence. These sequences are not resolved in the cryo-EM structure of the M35-Rubisco condensate (Wang H et al., Nature 2019). It seems unlikely that they contribute directly to the interaction, although this cannot be completely ruled out. A detailed analysis of their role is beyond the scope of the present study.

- Results: in the structure part the direct link between the observed interactions and multivalency is not clear.

The structural model of the multivalent interaction between the γCAL trimer and three C2 peptide sequences of CcaA, based on the crystal structure and on the binding stoichiometry determined by ITC in solution, is now shown as a main figure (Fig. 3b – Right panel). Since each γCAL trimer binds one CcaA subunit of a CcaA tetramer, this indicates, in principle, that one γCAL trimer has the ability to interact with three CcaA tetramers, resulting in a multivalent network. Whether three tetramers bind simultaneously would depend on steric effects and the dynamics of the interaction. We have explained this more clearly in the revised manuscript.

- What is the role of disordered flanking regions and protein dynamics? Cryo-EM indicates protruding densities, which might be critical for the network assembly .

Please see point above. The protruding densities observed in the cryo-EM map corresponds to SSUL modules. This interaction is indeed critical for M58 homo-demixing as shown by the mutational analysis in Fig. 4f.

- The role of dynamics is not clear in the discussion it is mentioned as more dynamical state, in the results as a not dynamic association. Is it possible that salt concentration also affects the dynamics of protein chains?

Our study focuses on the mechanism of pre-carboxysome formation which occurs in the reducing environment of the cytosol. In the Discussion, we suggested a potential redox regulation, where condensate dynamicity might increase after shell formation within the oxidizing interior of the carboxysome (see Introduction and Fig. 1b).
Yes, indeed, salt concentration does affect condensate formation (see Fig. 1d,e, Supplementary Fig. 4f, Supplementary Fig. 7a-d). Thus, differences in salt concentration between the cytosol and the carboxysome interior could affect condensate dynamicity. There is, however, no information available whether such a difference exists.

References
- cite head-to-tail polymerisation as a mechanism for phase separation (PMID 32822572) or for less dynamic assemblies (PMID 27203110)

Thank you for bringing these references to our attention, they are now included in the revised manuscript.

Reviewer #2 (photosynthesis, carboxysomes)
Remarks to the Author:

Summary of the key results:
The proteinaceous microcompartments known as carboxysomes found in cyanobacteria have been a great interest to the photosynthesis research community because these bacterial organelles are highly effective in concentrating CO2 around Rubisco. In β-carboxysomes, the main scaffolding protein, CcmM, is known to function as two isoforms: M58, which is the full-length version, and M35, which is the shorter version with only the C-terminal part of M58. Previous studies showed that both isoforms are essential components of β-carboxysomes. M35 is made up of three repeats of Rubisco small subunit-like (SSUL) modules, and previous work (Wang et al, Nature, 566, 131-135, 2019) detailed how it forms condensate with Rubisco through structural and biochemical methods. The N-terminal part of M58 contains a γ carbonic anhydrase-like domain (γ-CAL) and has been known to interact with at least two other proteins of β-carboxysomes, CcmN and βCA. However, the detailed mechanism of the interecations between M58 and other proteins are not known. In this manuscript, Zang et al describe their findings from biochemical and structural studies of M58, βCA, Rubisco and M35. They found that M58 is able to form three different types of condensates: with βCA, Rubisco or by itself. The biochemical and structural experiments are well designed and clearly presented in the manuscript. Not only did they

address the critical interaction between M58 and βCA at the atomic levels, they also revealed two unexpected interactions: one between the SSUL modules and γ-CAL domain of M58 and the head-tohead interaction between γ-CAL domains from two M58 trimers. All these interactions lead to intricate network of proteins influenced by redox conditions. Hence, this manuscript is an important step towards understanding the detailed interactions among proteins inside β-carboxysomes and should be of great interest to the researchers studying β-carboxysomes as well as implementing them into plants for bioengineering purposes. Despite the intricate nature of the materials, the manuscript is well organized, and the findings are clearly described.

We thank the reviewer for her/his positive remarks.

Minor suggestions for the authors:
1. In line 202, when homo-demixing of M58 in Fig. 4c,d was described, it would be helpful to point out the difference between the experiments in Fig. 4c,d and those in Fig. 1b,c where M58 alone was shown not to form condensate.

We have clarified this point.

2. In Fig 8a ii, shouldn't the γCAL-CcaA condensate be salt "insensitive" based on Fig 2e?

We apologize for the confusion. In the case of the γCAL-CcaA interaction, increasing salt concentration enhances the interaction, consistent with the contribution of hydrophobic forces to binding. To distinguish between the different salt sensitivities, we introduced arrows down and up in the figure to indicate reduced and enhanced interactions, respectively.

3. The paper from Gonzalez-Esquer et al (Streamlined construction of the cyanobacterial CO2-fixing organelle via protein domain fusions for use in plant synthetic biology, The Plant Cell, 27, 2637-2644, 2015) previously showed that a single chimeric protein was able to structurally and functionally replace four proteins: M58, M35, βCA and CcmN. The chimeric protein was derived by replacing γCAL in M58 with a shorter version of βCA. It was not understood how the chimeric protein was able to replace both M58 and M35. Would the new findings in the current manuscript somehow provide some 'speculative' explanation regarding the unusual behavior of that chimeric protein? For example, the current manuscript suggests there is differential redox-regulation of the interactions between Rubisco and M58 versus M35. More specifically, the interaction between SSUL modules and γCAL in M58, which appeared enhanced under oxidizing conditions, is no longer present with the chimeric protein. Could the redox regulation of the chimeric protein be more similar to M35, while at the same time, it is more structurally and physically similar to M58? Can the authors provide some insights into the chimeric protein in

Gonzalez-Esquer et al paper in the discussion section?

The results of Gonzalez-Esquer et al. are consistent with our findings. Assuming the βCA domain forms a tetramer as does CcaA in our study, the number of SSUL modules in the fusion protein (CcmC) would be higher (12) than in M58 (9). This might compensate for the contribution of γCAL to condensate formation. As the same time, since the βCA domain provides the carbonic anhydrase activity, recruitment of CccA via γCAL is no longer needed. Moreover, by linking the encapsulation peptide of CcmN to βCA, CcmN (which normally interacts with the γCAL domains of M58) is also no longer needed for carboxysome formation. However, with this construct the redox-regulation of carboxysome biogenesis/function is probably disturbed, as evident by the effect on the abundance, size and shape of βcarboxysomes in CcmC expressing cells. While we thank the reviewer for bringing this interesting paper to our attention, we feel that discussing this work would require extensive explanation of the interaction of CcmN with shell proteins. Given the space limitations, we defer a discussion of the Gonzalez-Esquer et al. paper to a future study. However, the paper is now cited in a more general context in the Introduction.

Reviewer #3 (plant structural biology)

Remarks to the Author:

Carboxysome is a special microcompartment formed in cyanobacteria and algae that enclose the enzymes Rubisco and carbonic anhydrase to optimize photosynthetic carbon fixation. Understanding the biogenesis of carboxysome assembly and implementing an optimized carboxysome-like $CO2$ concentrating mechanism (CCM) into plants is a desirable strategy to increase the crop yield. In this study, the authors revealed a central role of the scaffolding protein CcmM M58 via initiating phase separation in the biogenesis of β-carboxysomes in cyanobacteria. Specially, the N-terminal γCAL domain of M58 mediate interactions with CcaA (support the CA activity), intermolecular interactions with the SSUL domain, and also head-to-head association of CcmM trimers. Overall, this study provides new sights into the biogenesis of β-carboxysome. I have a few concerns on the manuscript.

We thank the reviewer for critically reading the manuscript and her/his remarks.

1. In figure 2f and 3, it seems that W257 in the C2 peptide play crucial roles in mediating the interactions with the γCAL domain of M58. Further biochemical assays should be carried out to validate the structural model as well as the key involved residues.

We agree and have generated single point mutations W257A and R265D in both EGFP-C17 and CcaA to validate the structural model. Mutating Trp257 to Ala and Arg265 to Asp in the C2 sequence of CcaA reduced condensate formation with M58 by ~50%, or completely abolished condensate formation,

respectively, as analyzed in the turbidity assay (see new Fig. 3d). Both mutations in EGFP-C17 abolished complex formation with γCAL by gel-shift assay (see new Supplementary Fig. 3i). These results underscore the specific contribution of hydrophobic and charge interactions to binding as indicated by the crystal structure.

2. CcaA exhibits as a tetramer in solution (Fig S1). It seems the preceding C1 peptide mediates the oligmerization. In the complex structure of γCAL-C2, each protomer of γCAL binds a C2 peptide. The conclusion "each protomer of the γCAL trimer interacts with one protomer of a CcaA tetramer" should be biochemically validated using full length proteins.

Deletion of more than 60 residues from the C-terminus of CcaA of Synechocystis PCC6803 has been shown to disrupt oligomer formation and enzymatic acitivity (So AK-C et al., Funct. Plant Biol. 2002). We have not independently validated the role of the C1 sequence in oligomerization. However, we now generated a construct in which the flexible C1 sequence, residues 205 to 219, were deleted and replaced by the sequence GSGGS in the CcaA protein of Se7942. This construct was still oligomeric by SECMALS and formed a condensate with M58 with wild-type efficiency, thus confirming the role of C2 as recognition sequence for binding to the γCAL domains. The role of C1 in oligomer formation would need to be investigated in the future. Note that the C1 sequence is not conserved between Se7942 and Synechocystis PCC6803.

The reviewer is correct in that we formerly do not know whether each γCAL protomer binds one CcaA tetramer. We only know that three monomers of EGFP-C217 can bind simultaneously (Supplementary Fig. 3h). However, for steric reasons, three CcaA tetramers may not be bound simultaneously, keeping in mind that these interactions are fluctuating. We apologize for the misleading statement, which we have changed to: "In summary, assuming the absence of steric hindrance, the γCAL trimer in the context of M58 may interact with 2 or 3 CcaA tetramers via the C2 sequence as the basis for condensate formation".

3. In Supplemental Fig 6, for the 3.6 Å resolution map, the color gradient of the local resolution maps of side and end views from blue to red indicates local resolution from 2.0 to 6.0 Å. I suggest the authors show the values in a shorter range (3.0 to 6.0 Å?).

We have made the requested change.

4. In Supplemental Fig 8, the authors showed the structural model of M58-RbcL8 to further validate the interaction between M58 and Rubisco complex. As the resolution of M58-RbcL8 is only 8 Å, information

provided from the model is very limited and not reliable. I think this part is not necessary in the current manuscript.

We respectfully disagree. For a map with 8 Å resolution, the observation of the presence or absence of the SSUL module (11 KDa) is still reliable. Please note that to validate the map, we performed two rounds of 3D classification focusing on either the SSUL or the RbcS position on the RbcL2 unit (Supplementary Fig. 8f). We consider this finding important evidence that SSUL cannot replace RbcS, and thus would like to maintain the data in the manuscript.

5. In supplementary Table 3, the authors should provide more parameters to show the model validations.

Please note that for all the reported cryo-EM structures no models were generated, and thus no model validations are required. Due to the low resolution of the EM maps, we docked the published PDB crystal structures into the EM density maps. The PDB codes mentioned in Table 3 were used to generate an initial 3D EM map for 3D classification during EM data processing.

6. In figures 1, 4, 7 and supplementary figs 2, 4, 10, the authors show the condensates of different proteins. In some figures, the authors exhibited the FRAP assays. It will be better to additionally show the results of droplet fusion.

As suggested, we analyzed droplet fusion for the various condensates. We find that the M58-CcaA and M58-M58 condensates do not undergo fusion during the 20 min observation period. Droplet fusion of M58 and Rubisco is observed, and the rate is very similar to that previously observed for M35-Rubisco droplets (Wang H et al., Nature 2019) (see new Fig. 6f and Supplementary Movies 3 and 4). The 4-protein condensate (Rubisco, M58, M35 and CcaA), on the other hand, undergoes only slow fusion (see new Fig. 7e and Supplementary Movie 5). We speculate that this feature of slow fusion propensity may be relevant in limiting pre-carboxysome size prior to shell formation. Note that aberrantly large carboxysomes are less efficient in the CO2 concentrating mechanism and high CO2 levels are required for cell survival(Wang H et al, Nature 2019; Sun Y et al, Plant Cell 2019). We thank the reviewer for allowing us to consider this possibility, which is now discussed in the revised manuscript.

7. The authors demonstrated that Rubisco, M58, M35 and CcaA could co-assembly in vitro. How about in vivo? It will be better to confirm the co-assembly in vivo.

Previous in vivo analyses have clearly shown that Rubisco, M58 , M35 and CcaA are colocalized within

carboxysomes (Long BM et al., J. Biol. Chem. 2007; Long BM et al., Plant Physiol. 2010; Niederhuber MJ et al., Mol. Biol. Cell 2017), and all 4 proteins can be pulled-down using N-terminally His-tagged M58. Unfortunately, it has not been possible to date to capture pre-carboxysomes and demonstrate the coassembly of the various proteins in vivo. Demonstration of such co-assembly in intact cyanobacterial cells will require a major effort, which is beyond the scope of this study.

8. In figure 6, the authors demonstrated that trimeric M58 binds rubisco with high affinity in a turbidity assay. Maybe some additional assays such as ITC will be better.

Unfortunately, ITC measurements of condensate forming mixtures are not possible as they result in clogging of the ITC chamber. Moreover, the absence of a defined stoichiometry would make any interpretation of such data difficult. Importantly, we compare the apparent M58-Rubisco affinity measured by the turbidity assay in Fig. 6a with our previous measurements for the M35-Rubisco interaction (Wang H et al., Nature 2019) using the same method.

9. CcmM directs multiprotein phase separation in β-carboxysome biogenesis. How about the effect of this phase separation on carbon fixation? Whether the ability of β-carboxysome carbon fixation depends on this phase separation?

Condensate formation of Rubisco, CcaA and other proteins, involved in carbon fixation, is the first step in the biogenesis of carboxysomes, and as such is required for the formation of a functional CCM. This condensate formation is mediated by the scaffolding protein CcmM (M58 and M35 in Synechococcus elongatus PCC 7942). However, once the carboxysome proteinaneous shell has formed, the material property of the condensate is most likely no longer critical to maintain the compartment. Thus, metabolic compartments, including carboxysomes, would differ from membraneless condensates, such as stress granules or the nucleolus, which do not have a shell and are dissociable. In fact, some of the multivalent interactions in the pre-carboxysome condensate are weakened (Wang H et al., Nature 2019) after shell formation, as the interior of the carboxysome is oxidizing (Price GD et al., Plant Physiol. 1992; Peña KL et al., PNAS 2010; Chen AH et al., PLoS One 2013). These aspects are now discussed more clearly in the revised manuscript.

Decision Letter, first revision:

16th Sep 2021

Dear Dr. Hayer-Hartl,

Thank you for your patience as we've prepared the guidelines for final submission of your Nature

Structural & Molecular Biology manuscript, "Scaffolding Protein CcmM Directs Multiprotein Phase Separation in β-Carboxysome Biogenesis" (NSMB-A44989A). Please carefully follow the step-by-step instructions provided in the attached file, and add a response in each row of the table to indicate the changes that you have made. Please also check and comment on any additional marked-up edits we have proposed within the text. Ensuring that each point is addressed will help to ensure that your revised manuscript can be swiftly handed over to our production team.

We would like to start working on your revised paper, with all of the requested files and forms, as soon as possible (preferably within two weeks). Please get in contact with us if you anticipate delays.

When you upload your final materials, please include a point-by-point response to any remaining reviewer comments.

If you have not done so already, please alert us to any related manuscripts from your group that are under consideration or in press at other journals, or are being written up for submission to other journals (see: https://www.nature.com/nature-research/editorial-policies/plagiarism#policy-on-duplicate-publication for details).

In recognition of the time and expertise our reviewers provide to Nature Structural & Molecular Biology's editorial process, we would like to formally acknowledge their contribution to the external peer review of your manuscript entitled "Scaffolding Protein CcmM Directs Multiprotein Phase Separation in β-Carboxysome Biogenesis". For those reviewers who give their assent, we will be publishing their names alongside the published article.

Nature Structural & Molecular Biology offers a Transparent Peer Review option for new original research manuscripts submitted after December 1st, 2019. As part of this initiative, we encourage our authors to support increased transparency into the peer review process by agreeing to have the reviewer comments, author rebuttal letters, and editorial decision letters published as a Supplementary item. When you submit your final files please clearly state in your cover letter whether or not you would like to participate in this initiative. Please note that failure to state your preference will result in delays in accepting your manuscript for publication.

Cover suggestions

As you prepare your final files we encourage you to consider whether you have any images or illustrations that may be appropriate for use on the cover of Nature Structural & Molecular Biology.

Covers should be both aesthetically appealing and scientifically relevant, and should be supplied at the best quality available. Due to the prominence of these images, we do not generally select images

featuring faces, children, text, graphs, schematic drawings, or collages on our covers.

We accept TIFF, JPEG, PNG or PSD file formats (a layered PSD file would be ideal), and the image should be at least 300ppi resolution (preferably 600-1200 ppi), in CMYK colour mode.

If your image is selected, we may also use it on the journal website as a banner image, and may need to make artistic alterations to fit our journal style.

Please submit your suggestions, clearly labeled, along with your final files. We'll be in touch if more information is needed.

Nature Structural & Molecular Biology has now transitioned to a unified Rights Collection system which will allow our Author Services team to quickly and easily collect the rights and permissions required to publish your work. Approximately 10 days after your paper is formally accepted, you will receive an email in providing you with a link to complete the grant of rights. If your paper is eligible for Open Access, our Author Services team will also be in touch regarding any additional information that may be required to arrange payment for your article.

Please note that <i>Nature Structural & Molecular Biology</i> is a Transformative Journal (TJ). Authors may publish their research with us through the traditional subscription access route or make their paper immediately open access through payment of an article-processing charge (APC). Authors will not be required to make a final decision about access to their article until it has been accepted. Find out more about Transformative Journals

<B>Authors may need to take specific actions to achieve compliance with funder and institutional open access mandates. For submissions from January 2021, if your research is supported by a funder that requires immediate open access (e.g. according to Plan S principles) then you should select the gold OA route, and we will direct you to the compliant route where possible. For authors selecting the subscription publication route our standard licensing terms will need to be accepted, including our self-archiving policies. Those standard licensing terms will supersede any other terms that the author or any third party may assert apply to any version of the manuscript.

Please note that you will not receive your proofs until the publishing agreement has been received through our system.

For information regarding our different publishing models please see our Transformative Journals page. If you have any questions about costs, Open Access requirements, or our legal forms, please contact ASJournals@springernature.com.

Please use the following link for uploading these materials:

[REDACTED]
If you have any further questions, please feel free to contact me.

Best regards,

Nyx Hills
Editorial Assistant
Nature Structural & Molecular Biology
nsmb@us.nature.com

On behalf of

Florian Ullrich, Ph.D.
Associate Editor
Nature Structural & Molecular Biology
ORCID 0000-0002-1153-2040

Reviewer #1:
Remarks to the Author:
The authors have addressed most of my comments , but still fail to provide evidence for the roles of disordered regions, and establish connections with multivalent interactions.
I think they still miss a key point for how condensates are formed, it is a pity that they have refused to perform at least some kind of bioinformatics predictions. Even if the key interaction is structured and specific, high entropy has to be achieved somehow.

Reviewer #2:
Remarks to the Author:
The authors have sufficiently addressed the concerns in the revised manuscript and added some additional experimental data to show the importance of the W257 and R265 residues of the C2 peptide from CcaA on its interaction with γCAL. Through multiple biochemical and biophysical approaches as well as structural studies, the authors have identified and characterized the nature of interactions underpinning the association of CcaA and Rubisco with the central organizing protein, M58. These interactions are critical to understand the synthesis of carboxysomes. I have the following minor comments.

1. P9 line189, it should be R265 instead of R126.
2. Fig. 2f, why did adding such short peptides as C2(15) and C2(17) to E-GFP change its mobility on the Native PAGE so dramatically? Whatever is slowing down its mobility appeared to be gone in R265D mutant of the C2(17) as can be seen in the Supplementary Fig. 3i.
3. Line 758, the name of the chemical, possibly KCl, in front of 100 mM is missing.
4. In Rubisco activity assay, please confirm that RuBP was present in excess in the reactions and was not rate-limiting. Based on my calculations, the reactions included 0.2 nmol of Rubisco active sites and 300 nmol of RuBP. If each Rubisco active site carboxylated 5 RuBP per second in the experiment, which is quite possible for the cyanoabacterial Rubisco at dissolved CO2 around its Km, all RuBP would be carboxylated in about 5 minutes. If the reactions were carried out under aerobic conditions, some RuBP would be oxygenated as well. Based on mixing 250 mM NaHCO3 and 4.5 mM NaH14CO3 with specific activity of 56.6 mCi/mmol), the amount of 14C activity counted after ending each reaction can be converted to mmol using 1 mCi/mmol. That amount should be well below 300 nmol to be able to compare the Rubisco activities across samples.

Reviewer #3:
Remarks to the Author:
The authors have addressed all my concerns. Congratulations to all the authors. This work is excellent.

Author Rebuttal, first revision:

Reviewer #1 (phase separation)
The authors have addressed most of my comments, but still fail to provide evidence for the roles of disordered regions, and establish connections with multivalent interactions. I think they still miss a key

point for how condensates are formed, it is a pity that they have refused to perform at least some kind of bioinformatics predictions. Even if the key interaction is structured and specific, high entropy has to be achieved somehow.

We appreciate the opportunity to again respond to the comments of Reviewer #1. We would like to note that the primary focus of our manuscript is to understand the mechanism of carboxysome biogenesis. The principles of condensate formation have been discussed in several excellent reviews, cited in the manuscript. We agree with the reviewer that the flexible regions linking the SSUL domains play an important role: by joining the SSUL modules, they generate the critical multivalency for the interactions of SSUL with Rubisco and the γCAL domains of M58. Furthermore, their dynamic nature likely helps to balance the entropy penalty that occurs upon SSUL binding to Rubisco or γCAL. We have added the following sentence to that effect in the discussion: "The flexible linkers between the SSUL modules apparently do not contribute directly to the interaction, but may play a role in balancing the entropic penalty of SSUL binding."

Reviewer #2 (photosynthesis, carboxysomes)

The authors have sufficiently addressed the concerns in the revised manuscript and added some additional experimental data to show the importance of the W257 and R265 residues of the C2 peptide from CcaA on its interaction with γCAL. Through multiple biochemical and biophysical approaches as well as structural studies, the authors have identified and characterized the nature of interactions underpinning the association of CcaA and Rubisco with the central organizing protein, M58. These interactions are critical to understand the synthesis of carboxysomes. I have the following minor comments.

We thank the reviewer for critically reading the revised manuscript.

1. P9 line189, it should be R265 instead of R126.

Corrected.

2. Fig. 2f, why did adding such short peptides as C2(15) and C2(17) to E-GFP change its mobility on the Native PAGE so dramatically? Whatever is slowing down its mobility appeared to be gone in R265D mutant of the C2(17) as can be seen in the Supplementary Fig. 3i.

Protein mobility on native-PAGE is determined by the size, shape and charge of a protein. Accordingly,

size markers are no reliable indicator of migration. By attaching the C217 peptide (one negative and two positive charges) to EGFP, we reduced the negative net charge of EGFP from -8 to -7. In addition, we changed the shape of the molecule, assuming that the C2 peptide represents a dynamically attached moiety that increases the hydrodynamic radius. Both changes are expected to slow migration on nativePAGE, consistent with the experimental result. Note that SEC-MALS analysis showed that all the EGFP fusion constructs are monomeric in solution. The single charge mutant R265D in the C2 peptide increased the negative net charge of the protein to -9, i.e. more negative than EGFP alone (-8). Thus, assuming that the additional negative charge partially compensates for the effect of the more expanded shape of the fusion protein compared to EGFP, the mutant would be expected to migrate similar to EGFP.

3. Line 758, the name of the chemical, possibly KCl, in front of 100 mM is missing.?

Corrected.

4. In Rubisco activity assay, please confirm that RuBP was present in excess in the reactions and was not rate-limiting. Based on my calculations, the reactions included 0.2 nmol of Rubisco active sites and 300 nmol of RuBP. If each Rubisco active site carboxylated 5 RuBP per second in the experiment, which is quite possible for the cyanoabacterial Rubisco at dissolved CO2 around its Km, all RuBP would be carboxylated in about 5 minutes. If the reactions were carried out under aerobic conditions, some RuBP would be oxygenated as well. Based on mixing 250 mM NaHCO3 and 4.5 mM NaH14CO3 with specific activity of 56.6 mCi/mmol), the amount of 14C activity counted after ending each reaction can be converted to mmol using 1 mCi/mmol. That amount should be well below 300 nmol to be able to compare the Rubisco activities across samples.

We appreciate the reviewer's concern. As suggested, we have performed the calculation using the 14C activity counts obtained after 5 min for Rubisco (RbcL8S8) on its own under both reducing and oxidizing conditions. In the presence of DTT we find a 14C activity of 152 nmol and of 158 nmol under oxidizing condition - well below 300 nmol. We conclude that the concentration of RuBP is not rate-limiting in our assays and that the Rubisco activities can be compared across the samples. The cpm data of the measurements are included in the source data file.

Reviewer #3 (plant structural biology)

The authors have addressed all my concerns. Congratulations to all the authors. This work is excellent.

We thank the reviewer for his/her kind remarks.

Final Decision Letter:

28th Sep 2021

Dear Manajit,

We are now happy to accept your revised paper "Scaffolding Protein CcmM Directs Multiprotein Phase Separation in β-Carboxysome Biogenesis" for publication as a Article in Nature Structural & Molecular Biology.

Acceptance is conditional on the manuscript's not being published elsewhere and on there being no announcement of this work to the newspapers, magazines, radio or television until the publication date in Nature Structural & Molecular Biology.

Before the manuscript is sent to the printers, we shall make any detailed changes in the text that may be necessary either to make it conform with house style or to make it intelligible to a wider readership. If the changes are extensive, we will ask for your approval before the manuscript is laid out for production. Once your manuscript is typeset you will receive a link to your electronic proof via email within 20 working days, with a request to make any corrections within 48 hours. Please read proofs with great care to make sure that the sense has not been altered. If you have queries at any point during the production process then please contact the production team at rjsproduction@springernature.com. Once your paper has been scheduled for online publication, the Nature press office will be in touch to confirm the details.

Please note that due to tight production schedules, proofs should be returned as quickly as possible to avoid delaying publication. If you anticipate any limitations to your availability over the next 2-4 weeks (such as vacation or traveling to conferences, etc.), please e-mail rjsproduction@springernature.com as soon as possible. Please provide specific dates that you will be unavailable and provide detailed contact information for an alternate corresponding author if necessary.

To assist our authors in disseminating their research to the broader community, our SharedIt initiative provides all co-authors with the ability to generate a unique shareable link that will allow anyone (with or without a subscription) to read the published article. Recipients of the link with a subscription will also be able to download and print the PDF.

As soon as your article is published, you can generate your shareable link by entering the DOI of your article here: <a href="http://authors.springernature.com/share">http://authors.springernature.com/share<a>.

Corresponding authors will also receive an automated email with the shareable link

Note the policy of the journal on data deposition:
http://www.nature.com/authors/policies/availability.html.

Your paper will be published online soon after we receive proof corrections and will appear in print in the next available issue. You can find out your date of online publication by contacting the production team shortly after sending your proof corrections. Content is published online weekly on Mondays and Thursdays, and the embargo is set at 16:00 London time (GMT)/11:00 am US Eastern time (EST) on the day of publication. Now is the time to inform your Public Relations or Press Office about your paper, as they might be interested in promoting its publication. This will allow them time to prepare an accurate and satisfactory press release. Include your manuscript tracking number (NSMB-A44989B) and our journal name, which they will need when they contact our press office.

About one week before your paper is published online, we shall be distributing a press release to news organizations worldwide, which may very well include details of your work. We are happy for your institution or funding agency to prepare its own press release, but it must mention the embargo date and Nature Structural & Molecular Biology. If you or your Press Office have any enquiries in the meantime, please contact press@nature.com.

You can now use a single sign-on for all your accounts, view the status of all your manuscript submissions and reviews, access usage statistics for your published articles and download a record of your refereeing activity for the Nature journals.

If you have not already done so, we strongly recommend that you upload the step-by-step protocols used in this manuscript to the Protocol Exchange. Protocol Exchange is an open online resource that allows researchers to share their detailed experimental know-how. All uploaded protocols are made freely available, assigned DOIs for ease of citation and fully searchable through nature.com. Protocols can be linked to any publications in which they are used and will be linked to from your article. You can also establish a dedicated page to collect all your lab Protocols. By uploading your Protocols to Protocol Exchange, you are enabling researchers to more readily reproduce or adapt the methodology you use, as well as increasing the visibility of your protocols and papers. Upload your Protocols at www.nature.com/protocolexchange/. Further information can be found at www.nature.com/protocolexchange/about.

An online order form for reprints of your paper is available at https://www.nature.com/reprints/author-reprints.html. Please let your coauthors and your institutions' public affairs office know that they are also welcome to order reprints by this

method.

Please note that <i>Nature Structural & Molecular Biology</i> is a Transformative Journal (TJ). Authors may publish their research with us through the traditional subscription access route or make their paper immediately open access through payment of an article-processing charge (APC). Authors will not be required to make a final decision about access to their article until it has been accepted. Find out more about Transformative Journals

<B>Authors may need to take specific actions to achieve compliance with funder and institutional open access mandates. For submissions from January 2021, if your research is supported by a funder that requires immediate open access (e.g. according to Plan S principles) then you should select the gold OA route, and we will direct you to the compliant route where possible. For authors selecting the subscription publication route our standard licensing terms will need to be accepted, including our self-archiving policies. Those standard licensing terms will supersede any other terms that the author or any third party may assert apply to any version of the manuscript.

In approximately 10 business days you will receive an email with a link to choose the appropriate publishing options for your paper and our Author Services team will be in touch regarding any additional information that may be required.

You will not receive your proofs until the publishing agreement has been received through our system.

If you have any questions about our publishing options, costs, Open Access requirements, or our legal forms, please contact ASJournals@springernature.com

Kind regards,
Florian

Florian Ullrich, Ph.D.
Associate Editor
Nature Structural & Molecular Biology
ORCID 0000-0002-1153-2040
